# Adipose-derived mesenchymal stem cells (AdMSC) for the treatment of secondary-progressive multiple sclerosis: A triple blinded, placebo controlled, randomized phase I/II safety and feasibility study

**DOI:** 10.1371/journal.pone.0195891

**Published:** 2018-05-16

**Authors:** Oscar Fernández, Guillermo Izquierdo, Victoria Fernández, Laura Leyva, Virginia Reyes, Miguel Guerrero, Antonio León, Carlos Arnaiz, Guillermo Navarro, Maria Dolores Páramo, Antonio De la Cuesta, Bernat Soria, Abdelkrim Hmadcha, David Pozo, Rafael Fernandez-Montesinos, Maria Leal, Itziar Ochotorena, Patricia Gálvez, Maria Angeles Geniz, Francisco Javier Barón, Rosario Mata, Cristina Medina, Carlos Caparrós-Escudero, Ana Cardesa, Natividad Cuende, on behalf of the Research Group Study EudraCT 2008-004015-35

**Affiliations:** 1 Unidad de Gestión Clínica de Neurociencias Clínicas, Servicio de Neurología y Servicio de Neurofisiología, Hospital Regional Universitario, Instituto de Investigación Biomédica de Málaga (IBIMA), University of Malaga, Malaga, Spain; 2 Servicio de Neurología, Hospital Universitario Virgen Macarena, University of Seville, Seville, Spain; 3 CABIMER (Andalusian Molecular Biology and Regenerative Medicine Centre), Seville, Spain; 4 Public Health Department, University of Malaga, Malaga, SPAIN; 5 Andalusian Initiative for Advanced Therapies, Junta de Andalucía, Seville, Spain; 6 Servicio de Radiodiagnóstico, Hospital Universitario Virgen Macarena, University of Seville, Seville, Spain; University Medical Center Gottingen, GERMANY

## Abstract

**Background:**

Currently available treatments for secondary progressive multiple sclerosis(SPMS) have limited efficacy and/or safety concerns. Adipose-mesenchymal derived stem cells(AdMSCs) represent a promising option and can be readily obtained using minimally invasive procedures.

**Patients and methods:**

In this triple-blind, placebo-controlled study, cell samples were obtained from consenting patients by lipectomy and subsequently expanded. Patients were randomized to a single infusion of placebo, low-dose(1x10^6^cells/kg) or high-dose(4x10^6^cells/kg) autologous AdMSC product and followed for 12 months. Safety was monitored recording adverse events, laboratory parameters, vital signs and spirometry. Expanded disability status score (EDSS), magnetic-resonance-imaging, and other measures of possible treatment effects were also recorded.

**Results:**

Thirty-four patients underwent lipectomy for AdMSCs collection, were randomized and thirty were infused (11 placebo, 10 low-dose and 9 high-dose); 4 randomized patients were not infused because of karyotype abnormalities in the cell product. Only one serious adverse event was observed in the treatment arms (urinary infection, considered not related to study treatment). No other safety parameters showed changes. Measures of treatment effect showed an inconclusive trend of efficacy.

**Conclusion:**

Infusion of autologous AdMSCs is safe and feasible in patients with SPMS. Larger studies and probably treatment at earlier phases would be needed to investigate the potential therapeutic benefit of this technique.

## Introduction

There are two main forms of multiple sclerosis (MS): relapsing remitting MS (RRMS), whose underlying pathophysiology is considered inflammatory, and secondary progressive MS (SPMS), which develops from RRMS [1] A primary progressive form (PPMS) also exists but is rarer 10–15% of cases of MS). In recent years, great progress has been made in the treatment of RRMS, with the availability of a range of new drugs that target the inflammatory component of the disease[2]. In the case of SPMS, whose pathological processes are thought to involve neurodegeneration,[3] little progress has been made and few treatments are authorized. Although interferon β is indicated for SPMS with disease activity (exacerbations or magnetic resonance imaging new, enlarging or enhancing lesions), the evidence for efficacy of this treatment is tenuous.[4] Mitoxantrone, in contrast, appears to have some impact on the disease [5] but has problems with long-term toxicity (cardiotoxicity and leukemia).[6] Ocrelizumab have recently been approved by the FDA for the treatment of PPMS [7]. PPMS is part of a spectrum of overlapping MS phenotypes with different pathogenesis. Thus, despite the highly debilitating nature of SPMS and the clear medical need, there is currently no truly effective treatment available.Recently, stem cells have demonstrated safety and a variable degree of efficacy in a diverse range of indications [8–17], but they are approved just for transplantation of blood stem cells to treat diseases and conditions of the blood and immune system, or to restore the blood system after treatments for specific cancers; skin stem cells to grow skin grafts for patients with severe burns on very large areas of the body and limbal stem-cell-based treatment to repair damage to the cornea. As of now, no treatments using mesenchymal stem cells have been licensed. There are, however, some clinical trials investigating the safety and effectiveness of MSC treatments for repairing bone or cartilage. Other trials are investigating whether mesenchymal stem cells might help repair blood vessel damage linked to heart attacks or diseases such as critical limb, help treat transplant rejection or autoimmune diseases are still under thorough investigation. In the case of MS, paracrine effects on central nervous system and the potential for increased endogenous axonal and myelin repair processes may be of benefit.[18,19] Encouraged by endogenous CNS repair demonstrated by stem cells in animal models of MS,[20] investigators aimed to test the effects of stem cells in humans [21] and a number of studies with intrathecal and/or intravenous administration of bone-marrow derived mesenchymal stem cells (BM-MSCs) in patients with MS have been reported.[22–25] Of particular note was an exploratory not blinded study of patients with SPMS involving visual pathways, in which the treatment was found to be safe and evidence was reported of structural, functional, and physiological improvement in visual endpoints. Particularly, the use of visual evoked potentials as an outcome measure proved to be of a great usefulness detecting subtle improvements, as the treatment demonstrated a decreased visual evoked response latency (-1·33 ms, 95%CI-2·44 to -0·21; p = 0·020).[26] Other studies have investigated both intravenous and intrathecal administration of BM-MSCs in patients with either MS or amyotrophic lateral sclerosis (ALS) [27], and RRMS patients,[28] showing that the procedures are safe and some evidence of immunomodulatory effects.

Adipose-derived stem cells (AdMSCs) are another source of MSC, [29] with the advantage that the samples for stem cell production can be taken with a minimally invasive lipectomy procedure. With the exception of a report of 3 patients treated with stromal vascular fraction (cells from unexpanded adipose samples) [30], and another two small studies [31,32] the potential of adipose tissue as a source of stem cells has not been explored. The aim of the present study was to assess the safety and feasibility of two different doses of AdMSCs administered by intravenous infusion in patients with SPMS.

## Methods

### Design and patients

In this two-center (Hospital Regional Universitario de Málaga and Hospital Virgen Macarena in Seville), phase I/II, placebo-controlled study, we randomized patients 1:1:1 to an intravenous infusion of placebo or one of two dose-groups (1x10^6^ cells/kg or 4x10^6^ cells/kg). The study was triple blinded; treating physician, patient and statisticians were unaware of treatment assignment. The laboratory staff producing the AdMSC drug product or placebo had no direct contact with the clinical staff. Randomization was performed at CABIMER with a random allocation sequence using sequentially numbered containers stratified by treatment center.

To be eligible for the study, adult patients of either sex had to have a diagnosis of SPMS, with an Expanded Disability Status Score (EDSS) between 5.5 and 9 and have failed previous therapies and have activity or progression of the disease (relapse in the previous year or progression of at least 0.5 points on the EDSS despite therapy). Patients were excluded if they had experienced a relapse or had received steroid treatment in the month prior to inclusion. Patients positive for HIV, hepatitis B, or hepatitis C, history of malignant neoplasms, participation in an interventional trial in the previous 3 months, contraindications for magnetic resonance imaging (MRI), history of liver, kidney, cardiac, or psychiatric disease that may have impacted the study procedures were also excluded. Finally, it had to be possible to collect 30 g of adipose tissue for the AdMSC preparation.

After infusion at the baseline visit, patients entered 1 year follow-up (with visits scheduled at 30 days, 6 months and 12 months after the baseline visit). Prior to any study procedures, full informed consent was obtained in writing from all participants. The study was approved by the corresponding ethics committee (Comité Coordinador de Ética de la Investigación Biomédica de Andalucía) and registered with the clinicaltrials.gov clinical registry (ID, NCT01056471) prior to initiation. The sponsor was the Andalusian Initiative for Advanced Therapies, supported by the Andalusian Health and Progress Foundation.

### Treatment protocol

#### AdMSC preparation

Abdominal subcutaneous adipose tissue from MS patients was obtained by lipectomy at the participating centers. The fresh adipose tissue was transported to the Good Manufacturing Practice (GMP) facility (Centro Andaluz de Biología Molecular y Medicina Regenerativa [CABIMER], Seville, Spain) at 2–20°C within 12 hours of collection.

Adipose tissue was washed at least three times with phosphate buffered saline (PBS) supplemented with a *Penicillin-streptomycin* mixture (Sigma-Aldrich, St Louis,MO,USA). The adipose tissue was minced and was enzymatically digested with an equal volume of 0.2% collagenase type I (Sigma-Aldrich) solution for 1 hour at 37°C with shaking. Cells were then separated by centrifugation at 600×g for 10 min after addition of 10% fetal bovine serum (FBS,SAFCBiosciences,Lenexa,Kansas,USA). The semisolid phase and the pellets were passed through a 100 μm cell-strainer to remove debris and cell clumps. Subsequently, the stromal vascular fraction (SVF) cells underwent a second centrifugation, and the isolated cells were re-suspended in a culture medium (Dulbecco’s modified Eagle’s medium[DMEM,Sigma-Aldrich]) supplemented with 10%FBS and 2%L-alanyl-L-glutamine (Sigma-Aldrich) for cell count and assessment of viability.

After adjusting the cell seeding concentration, cells were plated into polystyrene cell culture flasks and incubated at 37°C in humidified 5%CO_2_ atmosphere. After 24–72 hours of incubation, the medium was changed and non-adherent cells were removed. For AdMSC cultures, a complete change of medium every 2–3 days until reaching ≥ 80% confluence was applied. The AdMSCs were then washed with PBS and treated with 0.05% trypsin-ethylenediaminetetraacetic acid(EDTA)(Invitrogen) for 15 minutes to detach them from the surface of the culture flask and harvested with culture medium. The resulting suspension was re-plated in new cell culture flasks. AdMSC expansion proceeded this way until reaching the dose level to which the patient had been assigned (1x10^6^cells/kg or 4x10^6^cells/kg).

The cells were then harvested with basal medium after the treatment with trypsin-EDTA, centrifuged and counted in a Neubauer chamber. After counting, the AdMSCs were washed and resuspended in Ringer’s lactate supplemented with human serum albumin and glucose at the appropriate concentration, and then packaged in 50 mL-syringes with female luer-lock caps.

The finished product was released after meeting the following quality controls: immunophenotype (>90% positivity for CD90,CD73,CD105,CD13 and CD29, and <10%positivity for CD14/CD20/CD34/CD45/CD31 and HLA class II), negativity for mycoplasms, negativity for endotoxins, and no numeric or structural karyotype abnormalities. Results from the sterility test were obtained prior to infusion and 14 days after the release of the finished product. The placebo product consisted of Ringer’s lactate packaged in identical opaque 50 mL-syringes.

#### Administration

The AdMSCs were administered intravenously through a peripheral venous catheter over 2 hours, using an infusion pump, mounted on a laboratory shaker to avoid aggregation of the cells in the syringe. The speed of the infusion, number of cells infused per minute, was calculated to be similar to the habitual rate of transfusion of red cells. During all the procedures the blinding was assured by the appropriate measures (dark, opaque syringes and tubes were used). After infusion, patients remained in the clinic for 24 hours to monitor for possible adverse events (AEs).

Exhaustive data about the adequate dosing of AdMSCs intravenously administered in human treatment is lacking, so taking into account the existing literature [8–32], we estimated that the ideal number of cells to administer intravenously to our MS patients would be between 10^7^–10^10^.

### Outcome measures

Neurological assessments were performed by experienced neurologists at the participating centers. Safety was assessed primarily by monitoring for AEs and standard laboratory measures. Vital signs and spirometric parameters were also monitored. Possible treatment effects were assessed by changes in baseline, 6 and 12 months after infusion: clinical variables (number of exacerbations, EDSS), immunologic assessments, analysis of cerebrospinal fluid (CSF), MRI (T_2_-weighted lesions, T_1_ lesions, T_1_ gadolinium-enhanced lesions, volume, and magnetization transfer ratio-MTR), evoked potentials (EP) (visual-VEP, acoustic-BAEP, somatosensory-SEP and motor-MEP), optical coherence tomography (OCT) of the retina (retinal nerve fiber layer- RNFL), cognition measured with Paced Auditory Serial Addition Test (PASAT) and quality-of-life by questionnaire SF-36, EuroqoL-5D and MusiQoL.

### Statistical analysis

Given that this was an exploratory study and that previous efficacy and safety studies with AdMSCs were lacking, no formal sample size calculations were performed. In order to provide an indication of safety and dose-effect and to support future larger efficacy studies, 30 evaluable patients were considered sufficient.

For the purposes of this study, the safety analysis population was defined as all patients who underwent a lipectomy procedure. The intention-to-treat population comprised all patients who were randomized and received an infusion and the per protocol population comprised patients which accomplished the inclusion criteria with at least 1 post baseline visit.

Descriptive statistics were calculated for all outcome measures (means and standard deviations, standard errors, medians and interquartile ranges, or numbers and percentages, as appropriate). Baseline clinical and paraclinical data were compared to 12 months after treatment data using Student´s t test. The treatment effect of the two doses of treatment vs placebo was analyzed by non-parametric tests (Kruskal-Wallis test) and ANOVA of repeated measures for the clinical and paraclinical test performed at baseline, and 12 months.

## Results

### Study patients

**Fig 1:**
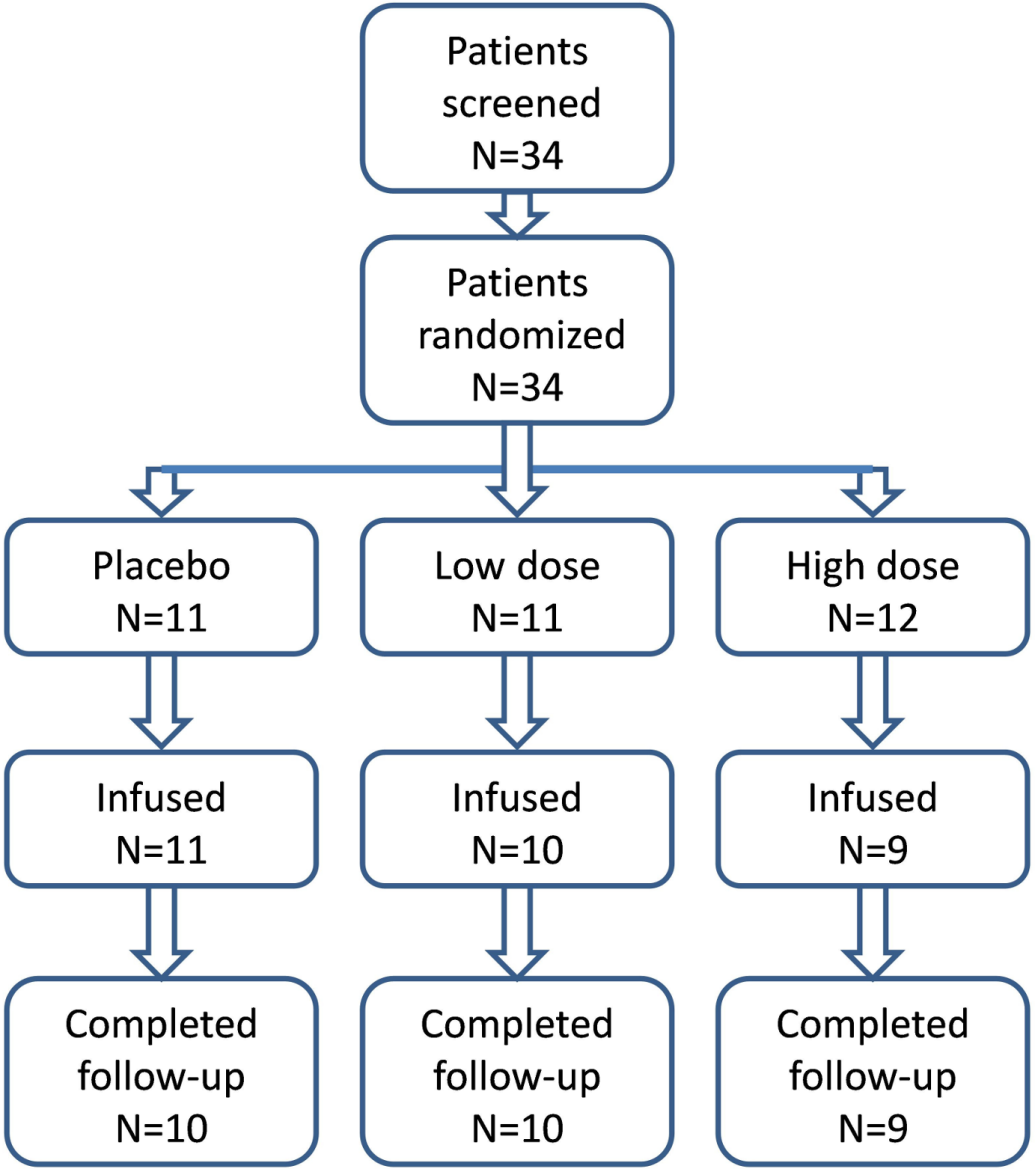
Patient disposition. Low dose = 1x10^6^ AdMSCs/Kg; High dose = 4 x 10^6^ AdMSCs/Kg.

The patient disposition is shown in Fig 1. 34 patients were randomized following lipectomy. Four randomized patients (3 assigned to the high dose group and 1 to the low dose group) were not infused because of karyotype anomalies in the cell product. There were no clinical differences of this group of 4 patients (age, EDSS or disease duration) when compared with the final infused group (data not shown). Thus 30 patients were infused and constitute the intention-to-treat population (ITT, Table 1). Of these 30 patients, 29 had at least 1 post-baseline assessment and were considered the per-protocol population. One patient had choking and bronchial aspiration while being fed 2 days after the infusion and died subsequently. All 29 patients in the per-protocol population completed 12 months follow-up. Analysis reported in this paper have been performed on all patients having had a procedure of lipectomy for tolerability and safety, according to predefined statistical analysis (Table 2) and on the ITT population for the efficacy variables.

**Table 1:** Baseline disease characteristics.

	Placebo (n = 11)	Low Dose (n = 10)	High Dose (n = 9)	Overall (n = 30)
**Demographic Characteristics**				
Sex				
Male	3(27%)	4 (40%)	2 (22%)	9 (30%)
Female	8 (73%)	6 (60%)	7 (78%)	21 (70%)
Age (years)	46.3±8.9	44.8±8.0	47.8±9.7	
**Disease Characteristics**				
EDSS	7.64±0.98	7.50±0.71	7.78±0.44	
Relapses				
1 in past 2 years	3 (27%)	5 (50%)	1 (11%)	9 (30%)
2 in past 2 years	0	0	2 (22%)	2 (7%)
1 in past year	2 (18%)	3 (30%)	3 (30%)	8 (27%)
Disease duration (years)	18.9±7.3	15.4±6.1	18.7±8.7	
MRI				
Gd-enhanced T_1_ lesions	0.82±1.17	0.70±1.57	2.0±2.88	
T_2_ lesions	159.0±78.4	152.4±49.9	140.75±35.3	

EDSS: expanded disability status scale; MRI: magnetic resonance imaging. Data presented as mean±SD or count (%)

**Table 2:** Adverse events during the trial.

	Placebo (n = 11)	Low Dose (n = 11)	High Dose (n = 12)	Active Treatment (n = 23)
AEs				
Patients with AEs	8 (73%)	5 (45%)	9 (75%)	14 (61%)
Number of AEs	27	8	35	43
Most frequent individual AEs				
Patients with urinary infection	3 (27%)	2 (18%)	1 (8%)	3 (13%)
Patients with respiratory infection	3 (27%)	0	1 (8%)	1 (4%)
Patients with anemia	2 (18%)	1 (9%)	2 (17%)	3 (13%)
Causality of AEs				
Unlikely	27	8	32	40
Likely	0	0	2	2
Possible	0	0	1	1
SAEs				
Patients with SAEs	3 (27%)^a^	0	1 (8%)	1 (4%)^b^

^a^ Choking, respiratory infection, urinary infection

^b^ Urinary infection

AE: adverse event; SAE: serious adverse event

Data presented as number (%).

The patients baseline characteristics are summarized in Table 1. 70% were women and ages ranged from 31 to 61 years. The disease duration ranged from 8 to 38 years. Most patients (25/30 [83%]) had monofocal symptoms at presentation and the baseline EDSS ranged from 6.0 to 9.0. Only 2 patients (7%) had experienced more than 1 relapse in the 2 years prior to enrolment. The mean number of Gd-enhanced T_1_ lesions was higher in the high-dose group (2.0±2.88 SD3.56±5.39) compared to the other groups, although this difference was driven by an outlier patient with 16 lesions (compared to maxima of 5 and 3 in the placebo and low-dose groups). Otherwise, the number of T_2_ lesions was not statistically different between groups.

### Safety results

Two patients in the placebo group, died during the study: one patient had choking and bronchial aspiration while being fed 2 days after the infusion and one patient had a respiratory infection nine months after infusion. They were women of 31 and 43 years old, with an EDSS at the beginning of the clinical trial of 9 and 8,5 and with 13 years of evolution of the disease in both cases.

A total of 70 AEs were reported during the trial period in 22 patients (67%). The most frequently reported individual AEs were urinary infection, respiratory infection and anemia (Table 2). Four serious AEs were reported (2 of these resulted in death, as described above). None of these serious AEs were considered related to treatment by the investigators and only 1 (urinary infection in a patient who already had a urinary infection on inclusion) occurred in an active treatment group (high-dose group).

No significant changes from baseline were observed for mean vital signs, spirometry or laboratory values, except for a significant decrease in cholesterol and creatinine in the low-dose group and a decrease in lymphocytes counts in the high-dose group, although the mean values remained within normal reference ranges.

### Treatment effects

Outcomes in treatment effects variables after 12 months of treatment are shown in Table 3. No variable showed statistically significant differences in repetitive measures neither by ANOVA (pFisher—p.F) nor by Kruskal-Wallis (p.KW) tests.

**Table 3:** Changes in treatment effects variables after 12 months of treatment.

		Placebo		Low dose		High dose	
	Variable	Baseline	Outcome (12 months)	Baseline	Outcome (12 months)	Baseline	Outcome (12 months)
	EDSS	7.64±0.31	-0.09±0.17	7.50±0.24	0.25±0.09*	7.78±0.16	0.28±0.52
MRI	MRI T1 number of lesions	62.18±7.18	1.22±0.68	46.40±9.45	0.20±0.81	40.56±6.70	-0.67±0.71
	MRI T1 area (cm^2^)	197.17±71.85	0.34±1.08	111.51±35.26	7.74±5.17	94.78±34.99	-0.93±1.43
	MRI T1GAD number of lesions	0.82±0.37	0.50±0.63	0.70±0.52	-0.10±0.25	2.00±1.09	-0.75±0.72
	MRI T1GAD area (cm^2^)	0.01±0.00	0.00±0.01	0.01±0.01	0.01±0.02	0.02±0.01	-0.01±0.01
	MRI T2 number of lesions	159.0±24.79	-1.30±2.79	152.40±16.62	2.50±2.67	140.75±13.34	2.50±1.82
	MRI T2 area (cm^2^)	440.7±130.1	16.4±9.7	298.9±67.0	8.66±5.16	296.70±57.61	23.78±19.49
	MRI normalized cerebral volume (cm^3^)	1420.6±24.2	-5.1±7.1	1483.7±26.7	-23.5±11.8	1449.2±32.6	-11.4±19.8
	MRI non normalized cerebral volume (cm^3^)	1062.9±37.94	-11.8±7.3	1112.7±28.9	-29.8±11.9*	1036.8±58.5	5.7±24.1
	MTR	18.1±0.51	-1.21±0.62	18.3±0.49	-2.00±0.62*	19.06±0.78	-2.0±0.9
Evoked	VEP p100 Latency (ms)	134.45±11.91	18.00±15.21	164.82±12.11	-0.58±13.63	156.49±9.63	-13.48±14.77
potentials	VEP amplitude (µV)	4.02±0.88	-0.71±0.71	3.26±0.78	-0.44±0.74	4.31±1.30	-2.41±0.78*
	BAEP I-V Interval (ms)	3.86±0.12	0.03±0.17	4.46±0.19	-0.24±0.25	4.26±0.15	0.04±0.15
	BAEP V/I amplitude	37.62±14.54	3.26±11.26	38.36±16.82	-5.69±17.83	25.50±12.49	-16.06±15.70
	Median nerve SEP (N13-N20) (ms)	12.82±1.14	-0.76±1.21	24.71±10.50	-10.49±10.64	17.57±5.85	-6.76±6.88
	Tibial nerve SEP (N22-P39) (ms)	43.62±2.64	-8.42±2.56*	44.38±1.61	-8.76±2.88*	45.74±1.60	-11.24±2.41*
	MEP superior CCT (ms)	48.25±11.26	-20.11±10.02	44.48±12.70	-16.15±10.55	51.76±15.61	-30.90±16.72
	MEP inferior CCT (ms)	72.70±5.73	-28.68±6.96*	75.92±6.30	-35.18±4.82*	75.07±7.25	-27.64±9.76*
	P300 Latency (ms)	380.14±32.04	42.24±46.39	332.20±21.48	22.13±11.44	373.92±21.61	-4.22±12.93
	EPAS	24.27±1.36	-0.10±1.57	23.70±1.99	1.90±1.07	22.78±1.32	1.43±1.00
OCT	Optic coherence tomography RNFL (μm)	70.95±6.11	-1.06±2.97	72.00±5.23	-1.94±3.56	67.56±5.27	-1.79±2.19
Cognition	PASAT	36.50±8.09	4.17±1.86	30.17±4.37	-2.60±2.41	31.00±7.10	0.25±3.24
and QoL	QoL (EQ5D)	45.91±8.27	-4.44±7.78	47.03±9.25	-2.53±6.95	50.00±7.76	1.88±6.62

EDSS: Expanded Disability Status Scale; MRI: magnetic resonance imaging; GAD: gadolinium enhanced; MTR: magnetization transfer ratio; VEP: visual evoked potential; BAEP: brainstem acoustic evoked potential; SEP: somatosensory evoked potential; MEP: motor evoked potential; CCT: central conduction time; EPAS: evoked potential abnormality score; OCT: Optic coherence tomography; RNFL: retinal nerve fiber layer; PASAT: Paced Auditory Serial Addition Test; EQ5D: EuroQol-5D; ms: milliseconds; μV: microvolts; μm: micrometer.

Data presented at baseline and after 12 months (change) as mean±SE

*P≤0,05 significative change (paired Student’s t test for changes within groups). No significant differences between groups were detected by Fisher`s -pF- and Kruskal-Wallis -pKW- tests.

The mean EDSS score did not show statistically significant variations over the course of the study (baseline EDSS for placebo, low-dose and high-dose groups were 7.64±0.314, 7.50±0.24, and 7.78±0.16 SE at baseline, compared to 7.55±0.35, 7.75±0.24, and 8.06±0.41 SE at 12 months p.F = 0,57; p.kW = 0,20). Individual EDSS changes were not significant as well. (See Supplementary Files S2 File—EDSS individual changes and S9 File—Changes in treatment effects variables after 12 months of treatment-complete data)

Baseline MRI data of the three groups were similar, with no significant differences. Non statistically significant post-baseline changes were observed in low and high dose group for number of active lesions in the Gd-enhanced T_1_ scans (although, as mentioned above, at baseline, an outlier patient in the high dose group had 16 active lesions). A decrease in MRI non normalized cerebral volume in low dose group and in MTR was observed, being significant in paired t-test between baseline and the 12 months outcome, although there were no differences between the three groups when compared between them (MRI non normalized cerebral volume: p.F = 0,23; p.kW = 0,28; MTR: p.F = 0,60; p.kW = 0,50).

Evoked potentials showed no significant baseline difference between groups. We found some non-statistically significant differences between the placebo and treatment groups for the evoked potentials parameters after 12 months of treatment. Tibial SEP central conduction time (N22-P39) and the MEP central conduction time for the legs, demonstrated statistically significant diminishing latencies over time in placebo and the two treatment groups, but these differences were not statistically significant comparing placebo and both treatment groups. Visual evoked potential-VEP and median nerve SEP (N13-N20) also showed a trend of stabilization or amelioration of latencies over time in treatment groups, although these differences didn´t reach statistically significance over the time, as well as between groups;(p.F = 0,59; p.kW = 0,89).

No significant changes from baseline were observed for the analysis of cerebral spinal fluid, OCT measurements, cognition or quality of life questionnaires.

The immunological parameters of this study will be published separately.

## Discussion

In this phase I/II proof-of-concept trial, intravenous infusion of AdMSCs was safe over the 12-month follow-up period. The pattern of AEs reported was one that would be expected from the underlying disease. No related serious AEs occurred and laboratory tests, vital signs, and spirometry did not identify any safety issues.

The safety findings from this study are in line with previous reports of stem cells in patients with SPMS, although these studies were performed with BM-MSC.[22–28] In the study reported by Connick et al,[26] 10 patients were intravenously infused with BM-MSCs, with the only AEs of note being 2 cases of infections and 2 skin reactions over up to 10.2 months of follow-up. In another study, Karussis et al [27] treated 15 patients with MS and 19 with ALS either intrathecally (34 patients) or intravenously (14 patients), the overall safety profile was good, with no major events reported during up to 25 months of follow-up, except for a case of meningeal irritation and aseptic meningitis in 1 patient. In the randomized, double-blind, placebo-controlled, crossover phase II clinical trial by Llufriu [28]^28^, 9 RRMS patients were treated and no serious adverse events were identified after 12 months of follow-up.

The most frequently reported individual AEs in this clinical trial were urinary infections, respiratory infections and anemia. None of these serious AEs were considered related to treatment by the investigators and only 1 (urinary infection in a patient who already had a urinary infection on inclusion) occurred in an active treatment group (high-dose group). Two patients in the placebo group, died during the study: one patient had choking and bronchial aspiration while being fed 2 days after the infusion and one patient had a respiratory infection nine months after infusion. Transplantation of stem cells can increase the risk of neoplasms. [33] No neoplasms were reported during our study after one year postinfusion. Animal studies with AdMSCs and MSC in humans have not detected any oncogenic potential.[34–36]

Evidence for a treatment effect was detected in the aforementioned studies with BM-MSCs. Connick et al,[26] reported evidence of structural, functional and physiological improvement after treatment in some visual endpoints. Karussis et al,[27]reported almost immediate immunomodulatory effects and a significant reduction in EDSS score in MS patients (from mean±SD 6.7± 1.0 before the intervention to 5.9±1.6 after the intervention). Llufriu [28], showed a trend but not significant treatment differences in the secondary efficacy endpoints.

Although data on possible markers of treatment effects were collected, our study with AdMSCs was not powered to demonstrate such effects and, unsurprisingly, no clear effects were detected. In the MRI studies, non statistically significant post-baseline changes were observed in the low and high dose group for number of active lesions in the Gd-enhanced T_1_. EP measures showed a statistically significant trend of efficacy by the reduction of latencies of somatosensory central conduction time for the arms, and some of the measured EP parameters: VEP latency, the tibial SEP central conduction time for the legs (N22-P39) and the MEP central conduction time for the arms and legs, demonstrated diminishing latencies over time, but these differences were not statistically significant with placebo. In clinical trials involving patients in advanced phases of MS, when the neurodegenerative phase of the disease is more prominent, dramatic effects are not to be expected, and there are no gold standard measures of neurodegeneration, we should look for measures that are able to detect even minimal but objective positive changes like evoked potentials have shown in other clinical trials [25] and in this clinical trial.

We have to note that the baseline mean EDSS score of our patients was greater than 7.5 whereas the patients included in the study by Connick et al [26,37] had a mean baseline EDSS of 6.1, implying that the patients in our study had more severe disease and that the baseline MRI, evoked potentials and OCT measures showed very much affected baseline parameters (very high number of baseline lesions in MRI, not recordable or very slow EP and very thin RNFL measured with OCT). This “ceiling effect” due to a severe disease is a well know bias in comparing this type of measures. when variance in an independent variable is not well measured or estimated above a certain level, and it impairs the ability of investigators to determine the central tendency of the data and differences between groups. We are afraid that this is the case of our possible markers of treatment.

Although several reports have used BM-MSCs,[22–26] very few studies have used expanded AdMSCs in patients with MS [31,32]. In view of the lack of robust data on treatment effects, we are unable to make affirmations about the relative merits of AdMSCs versus BM-MSCs. In principle, both are pluripotent stem cells that are able to undergo neural differentiation,[38] but their immunologic phenotypes do differ,[39] so their immunomodulatory properties may also vary.

Possible mechanisms of action of MSCs in SPMS include neuroprotection through paracrine modifications of the CNS microenvironment and increased endogenous axonal and myelin repair.[19,40]

From a procedural point of view, AdMSCs offer the potential advantage that the lipectomy or liposuction procedures, which we know now can be used alternatively, to obtain the cell sample are less invasive than bone marrow aspiration [29], and offer an alternative for the obtainment of the MSCs.

In conclusion, the present study demonstrates that infusion of AdMSCs is a safe and feasible procedure in patients with SPMS. Although the study was not powered to determine theefficacy, some hint of efficacy was observed by the use of MRI and evoked potentials. Larger studies would be needed to investigate the potential therapeutic benefit of the technique.

## Supporting information

S1 File Individual data file. (RAR)

S2 File Individual EDSS changes. (DOCX)

S3 File Complete clinical trial protocol. (PDF)

S4 File Complete clinical trial protocol translation. (DOCX)

S5 File Clinicaltrial gov full description. (PDF)

S6 File Clinical trial gov summary. (PDF)

S7 File Eudra protocol summary. (DOCX)

S8 File CONSORT checklist. (DOCX)

S9 File Changes in treatment effects variables after 12 months of treatment-complete data. (PDF)
