# Supplementary material for: Adipose-derived mesenchymal stem cells (AdMSC) for the treatment of secondary-progressive multiple sclerosis: A triple blinded, placebo controlled, randomized phase I/II safety and feasibility study
Source: PLoS One. 2018 May 16;13(5):e0195891. doi: 10.1371/journal.pone.0195891 (PMC5955528; doi:10.1371/journal.pone.0195891)
Supplement: S2 File — (DOCX) [file pone.0195891.s002.docx]

Group A-High Dose group

Group B-Placebo group

Group C- Low dose group

**S2-Individual EDSS changes**
