# Supplementary material for: Adipose-derived mesenchymal stem cells (AdMSC) for the treatment of secondary-progressive multiple sclerosis: A triple blinded, placebo controlled, randomized phase I/II safety and feasibility study
Source: PLoS One. 2018 May 16;13(5):e0195891. doi: 10.1371/journal.pone.0195891 (PMC5955528; doi:10.1371/journal.pone.0195891)
Supplement: S3 File — (PDF) [file pone.0195891.s003.pdf]

PROTOCOLO CÓDIGO: CMM/EM/2008

EudraCT: 2008-004015-35

ENSAYO CLÍNICO MULTICÉNTRICO FASE I/II ALEATORIZADO Y CONTROLADO CON PLACEBO, PARA EVALUACIÓN DE SEGURIDAD Y FACTIBILIDAD DE LA TERAPIA CON DOS DOSIS DISTINTAS DE CÉLULAS TRONCALES MESENQUIMALES AUTÓLOGAS DE TEJIDO ADIPOSO (CETMAD) EN PACIENTES CON ESCLEROSIS MÚLTIPLE SECUNDARIAMENTE PROGRESIVA, QUE NO RESPONDEN ADECUADAMENTE A LOS TRATAMIENTOS REGISTRADOS.

Versión: Final

24 de junio de 2008

Promotor: Fundación Progreso y Salud

Este protocolo se realizará siguiendo ICH-GCP (CPMP/ICH/135/95), principios éticos de la Declaración de Helsinki y la Legislación Española vigente (RD 223/2004).

La información de este documento es confidencial y es propiedad de la Fundación Progreso y Salud. Por tanto, la información no debe ser divulgada, publicada, revelada o transferida de cualquier otro modo a una tercera parte, en ninguna forma, sin consentimiento por escrito de la Fundación Progreso y Salud. Sin embargo este documento puede ser revelado a los investigadores y potenciales investigadores, Autoridades Sanitarias nacionales y correspondientes Comités Éticos bajo la condición de que respeten su naturaleza confidencial.

FIRMA DEL PROMOTOR

He leído el protocolo de ensayo clínico "**Ensayo clínico multicéntrico fase I/II aleatorizado y controlado con placebo, para evaluación de seguridad y factibilidad de la terapia con dos dosis distintas de Células Troncales Mesenquimales Autólogas de Tejido Adiposo (CeTMAd) en pacientes con Esclerosis Múltiple Secundariamente Progresiva, que no responden adecuadamente a los tratamientos registrados."** versión final de 24 de junio de 2008 y acepto cumplir todo lo establecido en el mismo y todas las leyes y normas que sean aplicables, incluidas, pero no limitadas a, las directrices de Buena Práctica Clínica (GCP) de la Conferencia Internacional de Armonización (ICH) y los principios éticos de la Declaración de Helsinki.

________________________________ Firma: ____________________

Por parte del Promotor:

Fundación Progreso y Salud

Cargo:_________________________ Fecha: ____ /____ /______

FIRMA DEL INVESTIGADOR PRINCIPAL

He leído el protocolo de ensayo clínico "**Ensayo clínico multicéntrico fase I/II controlado y aleatorizado, para evaluación de seguridad y factibilidad de la terapia con dos dosis distintas de Células Troncales Mesenquimales Autólogas de Tejido Adiposo (CeTMAd) en pacientes con Esclerosis Múltiple Secundariamente Progresiva, que no responden adecuadamente a los tratamientos registrados"** versión final de 24 de junio de 2008 y acepto cumplir todo lo establecido en el mismo y todas las leyes y normas que sean aplicables, incluidas, pero no limitadas a, las directrices de Buena Práctica Clínica (GCP) de la Conferencia Internacional de Armonización (ICH) y los principios éticos de la Declaración de Helsinki.

________________________________ Firma: ___________________

Investigador Principal

Centro:_________________________ Fecha: ____ ____ ____

1. INDICE

1. INDICE 4
2. ABREVIATURAS 6
3. RESUMEN 7
3.1. Tipo de estudio 7
3.2. Identificación del promotor 7
3.3. Título del ensayo clínico 7
3.4. Código del protocolo 7
3.5. Investigadores principales 7
3.6. Centros en los que se prevé realizar el ensayo 7
3.7. Comité de Seguridad 7
3.8. Comités Éticos Inv. Clínica 7
3.9. Nombre y calificación de las personas responsables de la monitorización 7
3.10. Terapia Celular 7
3.11. Fase del ensayo clínico 7
3.12. Objetivo del estudio 7
3.13. Diseño 8
3.14. Enfermedad en estudio 8
3.15. Población en estudio 8
3.16. Duración del ensayo 8
4. INFORMACIÓN GENERAL 9
4.1. Identificación del ensayo (título) 9
4.2. Tipo de ensayo clínico 9
4.3. Descripción de los productos en estudio 9
4.4. Centros en los que se prevé realizar el ensayo 9
4.5. Datos relativos al promotor 9
4.6. Director técnico responsable de la elaboración/control del producto en investigación 9
4.7. Datos de los investigadores Principales del ensayo 10
4.8. Datos de Investigadores del ensayo 10
4.9. Centros en los que se realizará el ensayo 11
4.10. Duración prevista del ensayo 11
5. BASE RACIONAL DEL ENSAYO, JUSTIFICACIÓN Y OBJETIVOS 12
5.1. INTRODUCCIÓN. 12
5.2. JUSTIFICACIÓN 16
5.3. OBJETIVOS 16
5.4. REFERENCIAS BIBLIOGRÁFICAS 18
6. TIPO DE ENSAYO CLÍNICO Y DISEÑO DEL MISMO 20
6.1. FASE DE DESARROLLO 20
6.2. DISEÑO GLOBAL DEL ESTUDIO 20
6.3. MÉTODOS DE EVALUACIÓN 21
6.4. ASIGNACIÓN DEL TRATAMIENTO 24
6.5. TÉCNICAS DE ENMASCARAMIENTO 24
7. SELECCIÓN DE SUJETOS 26
7.1. POBLACIÓN A SER ESTUDIADA 26
7.2. CRITERIOS DE INCLUSIÓN 26
7.3. CRITERIOS DE EXCLUSIÓN 26
7.4. CRITERIOS DE RETIRADA Y ANÁLISIS PREVISTO DE LAS RETIRADAS Y ABANDONOS 27
7.5. INTERRUPCIÓN DEL ENSAYO 28
7.6. NÚMERO PREVISTO DE PACIENTES 29
7.7. IDENTIFICACIÓN DE LOS SUJETOS 29
8. DESCRIPCIÓN DEL TRATAMIENTO 30
8.1. EXTRACCIÓN DE TEJIDO APIDOSO 30
8.2. PREPARACIÓN DEL PRODUCTO EN INVESTIGACIÓN 30
8.3. PRESENTACIÓN DEL PRODUCTO EN INVESTIGACIÓN 31
8.4. IDENTIFICACIÓN DEL PRODUCTO EN INVESTIGACIÓN 32
8.5. ADMINISTRACIÓN DE LAS CÉLULAS MESENQUIMALES DE TEJIDO ADIPOSO 32

8.6. TRATAMIENTOS CONCOMITANTES PERMITIDOS Y PROHIBIDOS 32
9. DESARROLLO DEL ENSAYO Y EVALUACIÓN DE LA RESPUESTA............................ 33
9.1. EVALUACIÓN DE LA RESPUESTA... 33
9.2. DESARROLLO DEL ENSAYO 35
9.3. DISTRIBUCIÓN DE TAREAS... 44
10. ACONTECIMIENTOS ADVERSOS 45
10.1. COMITÉ DE SEGURIDAD.. 45
10.2. DEFINICIONES.. 45
10.3. REGISTRO Y COMUNICACIÓN DE LOS ACONTECIMIENTOS ADVERSOS.................. 47
10.4. VALORACIÓN DE LA CAUSALIDAD Y SEVERIDAD DE LOS AA.................................... 48
11. ASPECTOS ÉTICOS 50
11.1. CONSIDERACIONES GENERALES... 50
11.2. INFORMACIÓN QUE SE PROPORCIONARÁ A LOS SUJETOS Y TIPO DE CONSENTIMIENTO QUE SERÁ SOLICITADO EN EL ENSAYO.. 50
11.3. ACCESO A LOS DATOS DEL ESTUDIO... 51
11.4. PROTECCIÓN DE LOS DATOS OBTENIDOS EN EL ESTUDIO....................................... 51
11.5. SEGURO DEL ENSAYO.. 51
12. CONSIDERACIONES PRÁCTICAS 52
12.1. RESPONSABILIDADES DE LOS PARTICIPANTES DEL ENSAYO CLÍNICO. 52
12.2. MONITORIZACIÓN... 54
12.3. ENMIENDAS O MODIFICACIONES DEL PROTOCOLO 54
13. ANÁLISIS ESTADÍSTICO.. 55
13.1. POBLACIONES 55
13.2. TAMAÑO MUESTRAL 55
13.3. ANÁLISIS ESTADÍSTICOS... 55
13.4. DIFICULTADES Y LIMITACIONES DEL ESTUDIO 56
ANEXO I: RESUMEN DEL SEGUIMIENTO 57
ANEXO II: ESCALA DEL ESTADO DE DISCAPICAD EXPANDIDA... 58
ANEXO III: PROTOCOLO DE RESONANCIA MAGNÉTICA 60
ANEXO IV: PROTOCOLO NEUROFISIOLÓGICO.. 63
ANEXO V: PROTOCOLO NEUROPSICOLÓGICO.. 65
ANEXO VI: PROTOCOLO DE CALIDAD DE VIDA 67
ANEXO VII: TABLA DE LA OMS PARA GRADACIÓN DE LA TOXICIDAD 68
ANEXO VIII: IDENTIFICACIÓN DEL PRODUCTO EN INVESTIGACIÓN.................................... 72
ANEXO IX: PERFIL DE CITOCINAS y ANALISIS FUNCIONAL DE LA RESPUESTA INMUNE .. 74
ANEXO X: PROTOCOLO DE PROCESAMIENTO Y ANÁLISIS DE LÍQUIDO CEFALORRAQUÍDEO 76
ANEXO XI: CUADERNO DE RECOGIDA DE DATOS.. 83
ANEXO XII: DECLARACIÓN DE HELSINKI DE LA ASOCIACIÓN MÉDICA MUNDIAL (WMA)... 84
ANEXO XIII: HOJA DE INFORMACIÓN Y CONSENTIMIENTO INFORMADO PARA EL PACIENTE.. 88
ANEXO XIV: CERTIFICADO DE LA PÓLIZA DE SEGURO 89

2. ABREVIATURAS

CeTMAd	Células Troncales Mesenquimales Autólogas de Tejido Adiposo
MSC	Células Troncales Mesenquimales (*del inglés mesenchymal stem cells)*
CABIMER	Centro Andaluz de Biología Molecular y Medicina Regenerativa
AA	Acontecimientos adversos
AAG	Acontecimientos adversos graves
AEMPS	Agencia Española del Medicamento y Productos Sanitarios
CEIC	Comité Ético de Investigación Clínica
CRD	Cuaderno de recogida de datos
PNT	Procedimiento Normalizado de Trabajo
BPC	Buenas Prácticas Clínicas
LPDP	Ley de Protección de Datos Personales
CMM	Células Madre Mesenquimales
EM	Esclerosis Múltiple
EMRR	Esclerosis Múltiple Remitente-Recurrente
EMPP	Esclerosis Múltiple Primariamente Progresiva
OPCs	Precursores de Oligodendrocitos Endógenos
SNC	Sistema Nervioso Central
CT	Células Troncales
EMSP	Esclerosis Múltiple Secundaria Progresiva
ELA	Esclerosis Lateral Amiotrófica
BHE	Barrera Hemato-Encefálica
EAE	Encefalomielitis Alérgica Experimental
PS	Progresivo Secundario
EDSS	Puntuación en la escala ampliada de Kurtzke (en inglés; Expanded Disability Status Scale)
MSFC	Multiple Sclerosis Functional Composite
RM	Resonancia Magnética
TOC	Tomografía Öptica de Coherencia
BRB	Brief Repeatable Battery of Neuropsychological Test in Multiple Sclerosis de Rao
TM	Transferencia de Magnetización
ERM	Espectroscopía por Resonancia Magnética
OMS	Organización Mundial de la Salud
PFR	Espirometría Básica con Test de Difusión
GMP	Good Manufacturer Practice
PE	Potenciales Evocados
LCR	Líquido Cefalorraquideo

3. RESUMEN

**3.1. Tipo de estudio**	Aplicación de la terapia de regeneración celular con Células Troncales Mesenquimales de tejido Adiposo (CeTMAd) en pacientes con Esclerosis Múltiple secundariamente progresiva que no responden adecuadamente a los tratamientos registrados.
**3.2. Identificación del promotor**	Fundación Progreso y Salud<br>Avda. Américo Vespucio 5, bloque 2, 2º planta.<br>41092 – Isla de La Cartuja (Sevilla)<br>Tlf: 955 04 04 50; FAX: 955 04 04 57
**3.3. Título del ensayo clínico**	Ensayo clínico multicéntrico fase I/II aleatorizado y controlado con placebo, para evaluación de seguridad y factibilidad de la terapia con dos dosis distintas de Células Troncales Mesenquimales Autólogas de tejido Adiposo (CeTMAd) en pacientes con esclerosis múltiple secundariamente progresiva, que no responden adecuadamente a los tratamientos registrados.
**3.4. Código del protocolo**	CMM/EM/2008
**3.5. Investigadores principales**	**Dr. Óscar Fernández Fernández.**<br>Hospital Regional Universitario Carlos Haya. Málaga<br>**Dr. Guillermo Izquierdo Ayuso.**<br>Hospital Universitario Virgen Macarena. Sevilla
**3.6. Centros en los que se prevé realizar el ensayo**	Hospital Regional Universitario Carlos Haya. Málaga<br>Hospital Universitario Virgen Macarena. Sevilla
**3.7. Comité de Seguridad**	El Comité revisará los datos de factibilidad y seguridad y un análisis de riesgo/beneficio.
**3.8. Comités Éticos Inv. Clínica**	CEIC AUTONÓMICO DE ANDALUCÍA
**3.9. Nombre y calificación de las personas responsables de la monitorización**	Fundación Progreso y Salud ó una empresa designada a tal efecto
**3.10. Terapia Celular**	Células Mesenquimales Troncales procedentes de tejido adiposo.<br>Forma producto en investigación: suspensión celular<br>Vía de administración: vía intravenosa
**3.11. Fase del ensayo clínico**	Fase I/II
**3.12. Objetivo del estudio**	Evaluar la seguridad y factibilidad del tratamiento regenerativo con Células Mesenquimales Troncales de Tejido Adiposo administradas por vía intravenosa en pacientes con Esclerosis Múltiple Secundariamente Progresiva no respondedores a tratamientos registrados. Se analizarán las

		complicaciones derivadas de la terapia regenerativa y/o procedimientos del estudio.
**3.13.**	**Diseño**	Prospectivo, multicéntrico, triple ciego, aleatorizado, controlado con placebo.
**3.14.**	**Enfermedad en estudio**	Pacientes con Esclerosis Múltiple Secundariamente Progresiva.
**3.15.**	**Población en estudio**	Se incluirán un total de 30 pacientes (10 pacientes por cada brazo del estudio)
**3.16.**	**Duración del ensayo**	Desde la infusión de las CeTMAd al paciente hasta el fin del seguimiento serán 12 meses.<br><br>Periodo de reclutamiento: el periodo estimado es de un mínimo de 12 hasta 24 meses desde la inclusión del primer paciente en el estudio.<br><br>Fecha prevista de inclusión del primer paciente: primer semestre de 2009.<br><br>Fecha prevista de inclusión del último paciente: primer semestre de 2011.<br><br>Fecha fin de seguimiento del último paciente incluido: primer semestre de 2012.

4. INFORMACIÓN GENERAL

4.1. Identificación del ensayo (título)

1. Código del protocolo: CMM/EM/2008
2. Título: Ensayo clínico multicéntrico fase I/II controlado y aleatorizado, para evaluación de seguridad y factibilidad de la terapia con dos dosis distintas de Células Troncales Mesenquimales Autólogas de Tejido Adiposo (CeTMAd) en pacientes con esclerosis múltiple secundariamente progresiva, que no responden adecuadamente a los tratamientos registrados.

4.2. Tipo de ensayo clínico

Ensayo clínico fase I/II, prospectivo, multicéntrico, aleatorizado, triple ciego, controlado con placebo.

4.3. Descripción de los productos en estudio

- Denominación: Células Troncales Mesenquimales de Tejido Adiposo (CeTMAd).
- Naturaleza del producto en investigación: suspensión celular.
- Composición cualitativa: volumen final óptimo entre 10-30 ml en función de la dosis.
- Dosis de producto: se emplearán dos niveles de dosis.

  1) $1x10^{6}$ células/ kg peso paciente

  2) $4x10^{6}$ células/ kg peso paciente

4.4. Centros en los que se prevé realizar el ensayo

Hospital Regional Universitario Carlos Haya. Málaga
Hospital Universitario Virgen Macarena. Sevilla

4.5. Datos relativos al promotor

Fundación Progreso y Salud
Avda. Américo Vespucio 5, bloque 2, 2º planta.
41092 – Isla de La Cartuja (Sevilla)
Tlf: 955 04 04 50; FAX: 955 04 04 57

4.6. Director técnico responsable de la elaboración/control del producto en investigación

Dr. Abdelkrim Hmadcha
Centro Andaluz de Biología Molecular y Medicina Regenerativa
Edif. CABIMER - Avda. Américo Vespucio s/n
Parque científico y Tecnológico Cartuja 93
41092- Sevilla
Tlf: 954 468 207; FAX: 954 461 664

4.7. Datos de los investigadores Principales del ensayo

Dr. Óscar Fernández Fernández.
Servicio de Neurología
Hospital Regional Universitario Carlos Haya.
Málaga

Dr. Guillermo Izquierdo Ayuso.
Servicio de Neurología.
Hospital Universitario Virgen Macarena. Sevilla

4.8. Datos de Investigadores del ensayo

- Guillermo Navarro Mascarell. Hospital Universitario Virgen Macarena. Sevilla.
- Miguel Ángel Gamero García. Hospital Universitario Virgen Macarena. Sevilla.
- Juan Luis Ruiz Peña. Hospital Universitario Virgen Macarena. Sevilla.
- Mª Dolores Páramo Camino. Hospital Universitario Virgen Macarena. Sevilla.
- Luminita Dinca. Hospital Universitario Virgen Macarena. Sevilla.
- Mónica Borges Guerra. Hospital Universitario Virgen Macarena. Sevilla.
- Carlos Caparrós Escudero. Hospital Universitario Virgen Macarena. Sevilla.
- Leticia Lledó Villar. Hospital Universitario Virgen Macarena. Sevilla.
- Noelia Becerril Ríos. Hospital Universitario Virgen Macarena. Sevilla.
- José Antonio García Alonso. Hospital Universitario Virgen Macarena. Sevilla.
- Miguel Lucas Lucas. Hospital Universitario Virgen Macarena. Sevilla.
- Mª Isabel García Sánchez. Hospital Universitario Virgen Macarena. Sevilla.
- Ana Isabel Venegas Venegas. Hospital Universitario Virgen Macarena. Sevilla.
- Enrique de Ramón Garrido. Hospital Regional Universitario Carlos Haya de Málaga
- Miguel Guerreo Fernández. Hospital Regional Universitario Carlos Haya de Málaga
- Ana Alonso Torres. Hospital Regional Universitario Carlos Haya de Málaga
- Victoria E. Fernández Sánchez. Hospital Regional Universitario Carlos Haya de Málaga
- Beatriz Asenjo García. Hospital Regional Universitario Carlos Haya de Málaga
- Luis Muñoz Becerra. Hospital Regional Universitario Carlos Haya de Málaga
- Jose Carlos Arrabal Gómez. Hospital Regional Universitario Carlos Haya de Málaga
- Manuela España Contreras. Hospital Regional Universitario Carlos Haya de Málaga
- Gustavo Fernández Baca. Hospital Regional Universitario Carlos Haya de Málaga
- Ildefonso Fernández Baca. Hospital Regional Universitario Carlos Haya de Málaga
- Mª Jesús Pinto Medel. Fernández Baca. Hospital Regional Universitario Carlos Haya de Málaga
- Andrea Gosis Kiperman. Fernández Baca.

Hospital Regional Universitario Carlos Haya de Málaga
- Gloria Luque Fernández. Fernández Baca. Hospital Regional Universitario Carlos Haya de Málaga
- Fátima Díez de Baldeón Fernández. Fernández Baca. Hospital Regional Universitario Carlos Haya de Málaga
- Luisa Vergara Carrasco. Fernández Baca. Hospital Regional Universitario Carlos Haya de Málaga
- Jaime Cordero Morales. Fernández Baca. Hospital Regional Universitario Carlos Haya de Málaga
- Susana García Gómez. CABIMER.
- Abdelkrim Hmadcha. CABIMER.
- Magdalena Martínez. CABIMER
- Patricia Gálvez. CABIMER
- Miriam Punzano Teruel. CABIMER.
- María Bermejo González. CABIMER.
- Gloria Carmona Sánchez. Programa Andaluz de Terapia Celular y Medicina Regenerativa.
- Natividad Cuende Melero. Programa Andaluz de Terapia Celular y Medicina Regenerativa.
- Ana Cardesa Gil. Programa Andaluz de Terapia Celular y Medicina Regenerativa
- David Pozo. Universidad de Sevilla.

4.9. Centros en los que se realizará el ensayo

Hospital Universitario Carlos Haya

Hospital Universitario Virgen Macarena

4.10. Duración prevista del ensayo

Desde la infusión de las CeTMAd al paciente hasta el fin del seguimiento serán 12 meses

Periodo de reclutamiento: el periodo estimado es de un mínimo de 12 hasta 24 meses desde la inclusión del primer paciente en el estudio.

Fecha prevista de inclusión del primer paciente: primer semestre de 2009.

Fecha prevista de inclusión del último paciente: primer semestre de 2011.

Fecha fin de seguimiento del último paciente incluido: primer semestre de 2012.

El estudio se realizará en el marco de colaboración de los siguientes entes públicos: Centro Andaluz de Biología Molecular y Medicina Regenerativa (CABIMER), el Hospital Universitario Carlos Haya de Málaga y el Hospital Universitario Virgen Macarena de Sevilla.

5. BASE RACIONAL DEL ENSAYO, JUSTIFICACIÓN Y OBJETIVOS

5.1. INTRODUCCIÓN.

La esclerosis múltiple (EM) se origina en personas susceptibles genéticamente, sobre las que actuaría un factor ambiental desconocido, originando una respuesta inmune alterada, dirigida contra antígenos de la mielina del sistema nervioso central, dando lugar a una enfermedad inflamatoria, desmielinizante y neurodegenerativa. Con una incidencia anual de 3-5/100.000 y una prevalencia de 70/100.000 habitantes, unas 30.000/40.000 personas sufren la enfermedad en España, siendo la enfermedad neurológica crónica más frecuente e invalidante en adultos jóvenes. El 85% de los casos debuta con brotes, forma remitente-recurrente (EMRR). La mayoría de los casos evolucionan a una forma con acumulación progresiva de la discapacidad o secundariamente progresiva (EMSP). Un 15% de pacientes tienen un curso progresivo desde el comienzo o forma primariamente progresiva (EMPP). Se trata inicialmente con inmunomoduladores, que reducen la enfermedad en un promedio de un 30%. Los casos que no responden se tratan con inmunosupresores que tienen una mayor eficacia, alrededor del 60%, pero son menos seguros (neoplasias, infecciones). Los casos especialmente agresivos, pueden tratarse con autotrasplante de células madre de estirpe hematopoyética, terapia excepcional, al conllevar un 4-8% de mortalidad. Pero, a pesar de todos los intentos terapéuticos mencionados, existen pacientes en los que las terapias fracasan y la actividad clínica y de Resonancia Magnética (RM) se mantiene, en particular porque la enfermedad es fundamentalmente inflamatoria (EMRR) en las fases iniciales, y más tarde esencialmente neurodegenerativa (EMSP), en la que las terapias disponibles no son eficaces, al ser todas ellas de perfil antiinflamatorio (Fernandez et al, 2005).

Necesidad de terapias neuro-reparadoras.

La remielinización espontánea tiene lugar en la EM, pero es muy limitada y disminuye con la progresión de la enfermedad. El fracaso de la remielinización puede deberse a una falta de migración o reclutamiento de los precursores de los oligodendrocitos endógenos (OPCs), fracaso de los OPC en la diferenciación a oligodendrocitos remielinizantes o a la existencia de un ambiente de señalización disrregulado. Las estrategias de reemplazo celular para promover la reparación en las enfermedades neurodegenerativas son áreas muy prometedoras de investigación. El trasplante de células madre ha sido efectivo en numerosos modelos experimentales, sin embargo, los mecanismos reparadores subyacentes permanecen desconocidos y puede que no predigan los resultados en humanos. Necesitamos terapias para revertir la discapacidad ya existente. Para establecer si la terapia con células madre es segura y eficaz en la EM, la investigación debe pasar a la clínica.

Funciones de las Células Troncales (CT) Mesenquimales Autólogas.

Las CT Mesenquimales Autólogas pueden derivar de la línea germinal. Se encuentran en múltiples órganos y tienen capacidades reparadoras, de crecimiento y de diferenciación. También pueden tener funciones reguladoras de los diferentes órganos, y pueden migrar a otros lugares para ejercer sus actividades reguladoras y regenerativas. Su diferenciación local viene guiada por el micro y macro-ambiente del órgano donde se encuentran (Strasser et al. 2007). En humanos, a nivel del Sistema Nervioso Central (SNC), las CT se encuentran en la zona subventricular y en el

área del hipocampo del cerebro. Su número va disminuyendo con la edad (Manganas, et al. 2007).

Argumentos para la aplicación clínica de las CT en la Esclerosis Múltiple (EM).

Estudios en animales de experimentación:
En el modelo de encefalitis alérgica experimental (EAE) en ratas se ha podido demostrar la presencia de CT neurales locales en un periodo de 10 días tras producir una lesión (Brundin et al. 2003). Los estudios experimentales aportan evidencias importantes a favor de la remielinización (Pluchino et al. 2004).

Estudios en humanos:
Recientemente se ha realizado trasplante de CT autólogas en pacientes con Esclerosis Lateral Amiotrófica (ELA) (Mazzini et al. 2003), lo que se ha considerado de bastante interés (Silani et al. 2004). También ha sido valorada positivamente la opción de tratamiento con CT en las enfermedades neurológicas en general y, más específicamente en la EM (Rice and Scolding 2004; Rice, et al. 2003; Barker, et al. 2003). Por otra parte, se ha destacado favorablemente el papel del tratamiento con CT mesenquimales en la enfermedad de injerto frente a huésped, en especial por su efecto inmunomodulador (Le Blanc, et al. 2004, 2008, Frank and Sayegh 2004). En ratones y en humanos se ha podido demostrar la transdiferenciación de CT mesenquimales y hematopoyéticas en neuronas (Mezey et al. 2000, Cogle et al. 2004). La experiencia acumulada a nivel internacional en el uso de células troncales mesenquimales en múltiples ensayos clínicos, varios de ellos en fase III, dirigidos a diversas patologías (www.clinicaltrials.gov) parece indicar que su uso es seguro, al menos a corto-medio plazo. No obstante, la experiencia de su aplicación en patologías del SNC es escasa. En un estudio de 9 pacientes con ELA no se presentaron efectos adversos importantes tras su inyección intraespinal (Mazzini, et al. 2008).
En consecuencia, el tratamiento con CT autólogas en la EM debería plantearse en términos de sus propiedades inmunomoduladoras (Nauta and Fibbe 2007), su hipotética capacidad de regeneración de neuronas, células gliales u oligodendrocitos y, por último, la posibilidad de conseguir remielinización, todo ello por mecanismos no bien conocidos entre los que se apuntan la transdiferenciación, la fusión celular (Ying et al. 2002) o la posible activación de células madre residentes por mecanismos paracrinos (Caplan and Dennis 2006).

Requerimientos para la aplicación clínica del tratamiento con CT autólogas en la EM.

Elección del donante:
Parece claro que son preferibles las CT mesenquimales autólogas (Rice et al. 2003; Rice and Scolding 2004). Las CT pueden obtenerse de la médula ósea del propio paciente que va a ser tratado. También pueden obtenerse del tejido celular subcutáneo (Técnica desarrollada en el Centro Andaluz de Biología Molecular y Medicina Regenerativa (CABIMER) donde se han desarrollado las células troncales mesenquimales de tejido adiposo CeTMAd).

Preparación de las células:
La recogida, almacenamiento, manipulación, chequeo sobre su estabilidad y presencia de anomalías cromosómicas o de otros tipos celulares y la prevención de las infecciones son técnicas estandarizadas en los institutos de investigación a nivel mundial, como por ejemplo el Centro Andaluz de Biología Molecular y Medicina Regenerativa (CABIMER). También están disponibles comercialmente en la actualidad.

Recientemente se ha podido demostrar (Shahdadfar et al. 2005) que las CT mesenquimales humanas pueden expandirse con rapidez y con una expresión genética estable en suero autólogo, en ausencia de factores de crecimiento.

Selección de pacientes:
El estadio de la enfermedad es muy importante para determinar los resultados que se pueden obtener con el tratamiento. En los pacientes con EM las lesiones son muy diferentes, tal y como se ha demostrado en recientes estudios patológicos y de imagen con RM. No obstante, el papel que las CT juega en los diferentes tipos patológicos no está aclarado. En principio, no habría razones para excluir ningún tipo de curso clínico de la EM para esta nueva forma de tratamiento. Sin embargo, por razones éticas, los mejores candidatos serían pacientes con EM del tipo progresivo secundario (PS), dada la ausencia de alternativas terapéuticas en este tipo de pacientes. Por su parte, los pacientes con formas Recurrentes Remitentes seguirían siendo candidatos a los estudios clínicos que ya están en desarrollo con otros tratamientos.

Necesidad de un protocolo del estudio:
Como siempre que se evalúa una intervención, es esencial disponer de un protocolo de actuación para obtener información cuantificada y válida. En este momento, el diseño del estudio más adecuado sería un ensayo clínico en sus fases iniciales (Fases I/II).

Aspectos éticos:
El tratamiento con CT autólogas es un procedimiento nuevo, y por lo tanto se seguirán escrupulosamente todos los aspectos éticos formulados en la declaración de Helsinki sobre investigación clínica en humanos, las directrices sobre Ensayos Clínicos en España del RD 223/2004 y de las Buenas Prácticas Clínicas, ICH-GCP (CPMP/ICH/135/95).

Forma de administración de las CT y dosis:
Los pacientes con EM presentan habitualmente una permeabilidad anormal de la barrera hemato-encefálica (BHE). Esta situación es más llamativa en los lugares en los que hay lesiones inflamatorias manifiestas, pero también está presente en el resto del cerebro y la médula espinal. Así mismo, existe evidencia de que los cambios en la BHE preceden al desarrollo de las lesiones inflamatorias en la EM (Jordan et al. 1999; Kermode et al. 1990).
Se ha investigado experimentalmente sobre la ruta de administración de CT de médula ósea autóloga en el tratamiento de patología cerebral y de médula espinal en ratas en las que se producen lesiones desmielinizantes con bromuro de etidio en la médula espinal (Inoue et al. 2003). Estas células estimulan la remielinización tras su infusión intravenosa (iv), así como tras su administración local. En este experimento con ratas con lesiones desmielinizantes en médula espinal se ha visto que con la administración iv se requiere inyectar 100 veces más células que con la administración local para conseguir la misma densidad de remielinización. Los resultados de la administración local son dosis-dependiente. Los experimentos de localización de células precursoras neurales multipotenciales en el modelo de EAE en ratas, ha demostrado la migración exclusiva de las células hacia las áreas lesionadas tras la inyección intraventricular e intratecal local (Ben-Hur et al. 2003). Además, en la EAE en ratas, se ha observado una correlación positiva entre el grado de inflamación en la médula espinal de la rata y el número de células precursoras multipotenciales que llegan al lugar inflamado. La administración iv de células de médula ósea pluripotenciales a ratas con lesión desmielinizante de la médula espinal, producida por bromuro de etidio, muestra una localización inmediata de estas células en el área lesionada (Akiyama et al. 2002).

De las tres formas posibles de administración, iv, intratecal y focal, la iv es la más sencilla y, en consecuencia, la preferible en la EM. El número de células que deberían utilizarse puede ser estimado sobre los datos de Inoue entre 10^7 y 10^{10} por vía iv. Incluso sin lesiones conocidas en el SNC, las CT atraviesan la BHE y forman neuronas en el cerebro (Mezey et al. 2000; Cogle et al. 2004). En los estudios realizados en pacientes con infarto de miocardio indican la necesidad de niveles entre 10^6–10^8 (Ng 2004). En los estudios llevados a cabo por Mazzini y colaboradores en la ELA se han utilizado dosis celulares en un rango entre 7 y $152 \cdot 10^6$ células (Manzini et al. 2008).

Destino de las células inyectadas:
Uno de los problemas más importantes en los ensayos clínicos de tratamiento con CT en EM es el de la localización de estas células tras su administración, así como su supervivencia. Recientemente, la RM por espectroscopía ha sido capaz de identificar y rastrear las células progenitoras neurales en el cerebro humano, así como monitorizar el destino de las CT autólogas. Mediante esta técnica es posible monitorizar la neurogénesis y la reparación en la EM, evaluando así las intervenciones terapéuticas (Magnanas et al. 2007).

Atrofia cerebral:
La Atrofia cerebral comienza en la EM es fases muy precoces de la enfermedad (Filippi et al. 2004). Por otra parte, no se explica completamente por el nivel de inflamación presente. Además, la atrofia cerebral muestra una lenta y continua progresión que no se ve influida por los tratamientos y se relaciona muy directamente con la situación de discapacidad del paciente. Patológicamente se observa la pérdida de las neuronas, axones y mielina. Es posible que el tratamiento con CT influya en la atrofia cerebral. En consecuencia, debe medirse la atrofia cerebral en los pacientes tratados.

Efectos adversos:
Los efectos adversos (EA) registrados en el tratamiento con CT son escasos. Aunque siempre existe la posibilidad de desarrollo de tumores, la transformación tumoral parece requerir periodos de cultivo in vitro extremadamente largos, superiores a 4 meses (Rubio et al. 2005). Hasta ahora no se han descrito EA en pacientes con ELA seguidos durante 2-4 años. Los efectos adversos precoces detectados, tal como dolor intercostal (resuelto en unos días) y disestesias sensitivas en las piernas (resueltas en unas semanas), se relacionaron con el procedimiento quirúrgico. No se observaron modificaciones del volumen de la médula espinal u otros signos indicativos de proliferación celular anómala (Mazzini et al. 2003 y Mazzini et al. 2008).

Justificación para pasar de la fase experimental a la clínica.
Aunque no existe unanimidad, la opinión actual de muchos investigadores es que se debe pasar ya de la fase de experimentación básica a la investigación clínica (Comi, 2008). En una revisión de las 32 presentaciones y discusiones comunicadas en una reciente reunión (Symposium on stem cell treatment in Multiple Sclerosis), organizada por The European Charcot Foundation, se ha hecho una evaluación crítica de esta cuestión (Hommes, 2008). El autor opina que el conocimiento del que disponemos debería llevarnos a iniciar los estudios experimentales en humanos. Actualmente existen diferentes centros clínicos europeos que tienen los recursos de infraestructura necesarios para desarrollar la fase clínica de investigación sobre el tratamiento con CT mesenquimales en EM, y de hecho, algunos de ellos los han iniciado en este momento (Ensayo Clínico fase I/IIA de la Universidad de Cambridge-www.clinicaltrials.gov). Los recursos económicos deben provenir de las instituciones locales o nacionales responsables de esos centros, ya que todavía no hay posibilidad de organizar un gran estudio multicéntrico de ámbito

supranacional, en el que The European Charcot Foundation podría jugar un papel fundamental como promotor a nivel europeo (European Clinical Trial of Stem Cell Treatment in MS-STEMS) y de la cual el investigador principal de este estudio es patrono desde hace muchos años y con la que trabaja habitualmente.

En este estudio se quiere iniciar la fase clínica de investigación de los resultados (seguridad y factibilidad) que pueden obtenerse con el tratamiento con CT mesenquimales autólogas en la EM de curso clínico secundariamente progresivo. Se trata de un ensayo clínico fase I/II, controlado y aleatorizado, triple ciego, en 30 pacientes, con 2 dosis de células mesenquimales obtenidas de tejido graso, monitorizado con una escala de efectos adversos, variables clínicas, analítica general e inmunológica, resonancia magnética, potenciales evocados, tomografía óptica de coherencia, pruebas neuropsicológicas y escalas de calidad de vida.

5.2. JUSTIFICACIÓN

La EM es una enfermedad inflamatoria crónica del SNC, que produce destrucción de la mielina. Los oligodendrocitos, así como las fibras nerviosas, también están afectados.

La localización multifocal de las lesiones en la EM hace que sea necesario plantear una implantación también múltiple en la EM, pero según estudios recientes en modelos animales, las células madre inyectadas por vía sistémica migran a las lesiones inflamatorias desmielinizantes, convirtiéndose algunas de ellas en precursores de oligodendrocitos y remielinizando axones. La mayoría de las células sin embargo permanecen indiferenciadas y actúan como supresoras de mecanismos inflamatorios (Pluchino et al. 2004).

5.3. OBJETIVOS

Objetivo principal

<u>Evaluar la seguridad de dos dosis de células troncales mesenquimales</u> autólogas administradas por vía intravenosa en pacientes con EM de curso clínico secundariamente progresivo. Se analizarán las complicaciones derivadas de la terapia regenerativa y/o procedimientos del estudio.

Objetivos secundarios

- <u>Seguridad</u>: Evaluar la seguridad de dos dosis de células troncales mesenquimales autólogas administradas por vía i.v. en pacientes con EM de curso clínico secundariamente progresivo, medida por escala de efectos adversos y análisis hematológicos y bioquímicos generales en las primeras 24h de la administración de las CeTMAd, 1 semana, 1 mes, 2 meses, 3 meses, 6 meses, 9 meses y 12 meses.
- <u>Factibilidad</u>: Evaluar la factibilidad de dos dosis de células troncales mesenquimales autólogas administradas por vía i.v. en pacientes con EM de curso clínico secundariamente progresivo, medida por variables clínicas (brotes, progresión medida con las escalas EDSS y MSFC) y paraclínicas (resonancia magnética, potenciales evocados, tomografía óptica de coherencia, pruebas neuropsicológicas y escalas de calidad de vida).
- Evaluar el efecto inmunomodulador de dos dosis de células troncales mesenquimales autólogas administradas por vía i.v. en pacientes con EM de curso clínico secundariamente progresivo. Para ello se procede a realizar mediciones de

subpoblaciones celulares y citocinas de perfil pro y antiinflamatorio en suero y líquido cefalorraquídeo. Asimismo, se procede al análisis funcional de la respuesta inmune en PBMCs, células B, células dendríticas y la actividad funcional de células $CD4^+CD25^+CD62L^{high}$ y $CD4^+CD25^-CD62L^{low}$.

- Evaluar el perfil de expresión génica en células PBMCs y células de LCR mediante "*arrays*", así como el estudio en paneles de *"antigen arrays"* y la evaluación de perfiles metabolómicos mediante NMR en suero y LCR con el objetivo de identificar nuevos biomarcadores con interés diagnóstico, pronóstico o de seguimiento.
- Se estudiará la factibilidad en pacientes de EM que no hayan respondido a otros tratamientos. Estará controlado con placebo.
- Aunque el período de seguimiento para la obtención de resultados de seguridad y factibilidad es de 12 meses, dado que estos pacientes se ven de forma rutinaria en consulta de los centros participantes, en el estudio se recopilarán datos a los 24 meses para obtener información adicional de seguridad, factibilidad y eficacia a medio plazo con un protocolo determinado y previa autorización de las autoridades sanitarias pertinentes.

5.4. REFERENCIAS BIBLIOGRÁFICAS

1. Akiyama Y. et al. Remyelination of the spinal cord following intravenous delivery of bone marrow cells. Glia 2003;39:229-236.
2. Barker R.A. et al. Stem cells and neurological disease. The therapeutic implications and application of stem cells for the nervous system. J. Neurol. Neurosurg Psychiatry 2003;74:553-557.
3. Ben-Hur T. et al. Transplanted multipotential neural precursor cells migrate into the inflamed white matter in response to experimental autoimmune encephalomyelitis. Glia 2003;41:73-80.
4. Le Blanc K. et al. Treatment of severe graft-versus-host disease with third party haploidentical mesenchymal stem cells. Lancet 2004: 363:1439-1441.
5. Le Blanc K. et al. Mesenchymal stem cells for treatment of steroidresistant severe, acute graft-versus-host disease: a phase II study. Lancet 2008:371:1579-86.
6. Brundin L. et al. Neural stem cells: a potential source for remyelination in neuro inflammatory disease Brain Pathol 2003;13:322-328.
7. Cogle C.R. et al. Bone marrow transdifferentiation in brain after transplantations: a retrospective study. Lancet 2004;363:1432-37.
8. Comi G. It is clinically relevant to repair focal multiple sclerosis lesions?. J Neurol Sci 2008;265:17-20.
9. Caplan AI, Dennis JE. Mesenchymal stem cells as trophic mediators. J Cell Biochem 2006;98(5):1076-84.
10. Fernández O, Fernández V, Guerrero M (eds). Epidemiologia. Patogenia. Clínica. Tratamiento. Esclerosis múltiple 2ed. McGraw-Hill/Interamericana. Madrid 2005.
11. Filippi M. et al. Interferon beta-1a for brain tissue loss in patients at presentation with syndromes suggestive of multiple sclerosis: a randomised, double-blind, placebo-controlled trial. Lancet 2004;364:1463-1464.
12. Frank M.H. and Sayegh M.H. Immunomodulatory functions of mesenchymal stem cells. Lancet 2004;363:1411-1412.
13. Hommes O.R.. Can we pass from the experimental to the clinical phase in MS stem cell research? J Neurol Sci 2008;265:136-139.
14. Inoue M. et al. Comparative analysis of remyelinating potential of focal and intravenous administration of autologous bone marrow cells into the rat demyelinated spinal cord. Glia 2003;44:111-118.
15. Jordan E. et al. Serial MR imaging of experimental autoimmune encephalomyelitis induced by human white matter or by chimeric myelin-basic protein and proteolipid protein in the common marmosed. Am. J. Neuroradiol. 1999;20:965-976.
16. Kermode A. et al. Breakdown of the blood-brain barrier precedes symptoms and other MRI signs of new lesions in Multiple Sclerosis. Pathogenisis and clinical implications. Brain 1990;113:1477-1489.
17. Manganas L, et al. Magnetic Resonance Spectroscopy Identifies Neural Progenitor Cells in the Live Human Brain. Science 2007;318:980-985.

18. Mazzini L. et al. Stem cell therapy in amyotrophic lateral sclerosis: a methodological approach in humans. Amyotroph Lateral Scler Other Motor Neuron Disor 2003;4:158-161.
19. Mazzini L. et al. Stem cell treatment in Amyotrophic Lateral Sclerosis. J Neurol Sci 2008;265:78-83.
20. Mezey E. et al. Turning blood into brain: cells bearing neuronal antigens generated in vivo from bone marrow. Science 2000;290:1779-82.
21. Nauta AJ, Fibbe E. Inmunomodulatory properties of mesenchymal stromal cells. Blood 2007; 110:3499-3506.
22. Ng Th. Stem cell therapy: what dose should we use? Lancet 2004; 364:1935-1936.
23. Pluchino S. et al. Cell-based remyelinating therapies in multiple sclerosis: evidence from experimental studies. Curr Opin Neurol 2004;17:247-255.
24. Pluchino S, Zanotti L, Rossi B, Brambilla E, Ottoboni L, Salani G, et al. Neurosphere-derived multipotent precursors promote neuroprotection by an immunomodulatory mechanism. Nature 2005;436:266–71
25. Rice C.M. et al. Stem cells for the treatment of neurological disease. Transfusion medicine 2003;13:351-361.
26. Rice C.M., Scolding N.J. Adult stem cells-reprogramming neurological repair. Lancet 2004; 364:193-199.
27. Rubio D el al. Spontaneous human adult stem cell transformation. Cancer Res 2005;65:3035-3039.
28. Shahdadfar A. et al. In vitro expansion of human stem cells: choice of serum is a determinant of cell proliferation, differentiation, gene expression and transcription stability. Stem cells 2005;230:1357-1366.
29. Silani V. et al. Stem cell therapy for amyotrophic lateral sclerosis. Lancet 2004;364:200-202.
30. Strasser H, et al. Autologous myoblasts and fibroblasts versus collagen for treatment of stress urinary incontinence in women: a randomised controlled trial. Lancet 2007;369:2179-86.
31. Ying QL, et al. Changing potency by spontaneous fusion. Nature 2002;416:545-548.

6. TIPO DE ENSAYO CLÍNICO Y DISEÑO DEL MISMO

6.1. FASE DE DESARROLLO

Se trata de un ensayo clínico multicéntrico fase I/II, de valoración de la seguridad y factibilidad de la terapia con células troncales mesenquimales autólogas de tejido adiposo (CeTMAd), en dos dosis distintas, en pacientes de esclerosis múltiple secundariamente progresiva, que no responden adecuadamente a los tratamientos registrados. Se trata de un estudio controlado, aleatorizado y triple ciego.

6.2. DISEÑO GLOBAL DEL ESTUDIO

6.2.1. DISEÑO

Estudio prospectivo, multicéntrico, triple ciego, aleatorizado, controlado con placebo y con dos grupos experimentales de tratamiento.

6.2.2. Tratamiento del ensayo. Dosis y pauta de tratamiento:

La población estará constituida por 30 pacientes con EM secundariamente progresiva y fracaso terapéutico. La distribución de los pacientes se hará en tres brazos:

- **Brazo 1 - Grupo Control:** 10 pacientes que recibirán como medicación de estudio sustancia placebo en las mismas condiciones que los grupos experimentales. El placebo se administrará en la visita basal y su composición será la misma que la de los grupos experimentales a excepción de la sustancia activa. La composición del placebo será:
    - Solución de Ringer lactada:
        - Cloruro cálcico dihidratado (2,7 mg/100ml)
        - Lactato sódico (320 mg/100ml)
        - Cloruro potásico (40 mg/100ml)
        - Cloruro sódico (600 mg/100ml)
    - 2,5% de glucosa
    - 1% albúmina.
- **Brazo 2 - Grupo Experimental 1:** 10 pacientes que recibirán como medicación de estudio una suspensión de células troncales mesenquimales (en la solución descrita para el grupo control) en una única dosis de $1 \cdot 10^6$ células/kg de paciente por vía i.v en bolo, tras haber cogido una vía periférica que se mantendrá con suero salino fisiológico, en la visita basal del estudio.

- **Brazo 3 - Grupo Experimental 2:** 10 pacientes que recibirán como medicación de estudio una suspensión de células troncales mesenquimales (en la solución descrita para el grupo control) en una única dosis de $4 \cdot 10^6$ células/kg de

paciente por vía i.v en bolo, tras haber cogido una vía periférica que se mantendrá con suero salino fisiológico, en la visita basal del estudio.

La inclusión en cada uno de los 3 brazos del estudio será aleatoria hasta alcanzar el tamaño de la muestra (30 pacientes), manteniendo una distribución equilibrada de pacientes en cada grupo.

Por otra parte, el estudio será detenido si se presentan algunas de las circunstancias contempladas como motivo de interrupción del ensayo.

6.2.3. Duración esperada de la participación de los sujetos. Secuencia y periodos del ensayo.

El periodo de inclusión de pacientes comprende un periodo de 12 hasta 24 meses. El seguimiento de los pacientes será de 12 meses y se llevará a cabo en 8 visitas según el esquema propuesto (véase Anexo I), por tanto los 30 pacientes serán evaluados de forma longitudinal para todos los parámetros clínicos y paraclínicos.

El estudio finaliza a los 12 meses de seguimiento del último paciente tratado, pero dado que estos pacientes se ven de forma rutinaria en consulta de los centros participantes en el estudio se recopilarán datos a los 24 meses para obtener información adicional de seguridad y factibilidad a medio plazo con un protocolo determinado.

En la visita de los 12 (ó en la visita rutinaria a los 24 meses) y, siempre que los resultados de factibilidad y seguridad lo permitan, se procederá a la infusión de células troncales mesenquimales del tejido adiposo a aquellos pacientes que hayan sido aleatorizados en el grupo control solicitando una extensión del estudio para tal procedimiento.

6.3. MÉTODOS DE EVALUACIÓN

6.3.1. Evaluación clínica

Un neurólogo efectuará la visita de preinclusión de cada paciente que comprobará la idoneidad del candidato que debe cumplir los criterios de inclusión y de exclusión en el proyecto. El paciente idóneo será convenientemente informado y debe firmar el consentimiento por escrito. A cada paciente incluido se le realizará una historia clínica, así como una exploración física y neurológica general, además de una evaluación específica de EM, con las escalas de Puntuación en la escala ampliada de Krutzke (EDSS) y Multiple Sclerosis Functional Composite (MSFC) (véase Anexo II). Se evaluará el historial de brotes y la progresión de la enfermedad en los años previos.

6.3.2. Obtención de tejido adiposo

Si el paciente es incluido, en el periodo de preinclusión se obtendrá, mediante técnicas de cirugía plástica un mínimo de 30 gramos de tejido graso limpio, de la pared abdominal, que se remitirán en frío (entre 2-8ºC) en un kit de transporte con un registrador de temperatura continuo, al laboratorio GMP para su elaboración ulterior.

6.3.3. Elaboración de las células CeTMAd

Laboratorio GMP CABIMER.
Director técnico responsable de la elaboración/control del producto en investigación:
Dr. Abdelkrim Hmadcha
Centro Andaluz de Biología Molecular y Medicina Regenerativa
CABIMER
Avda, Américo Vespucio s/n – Edif. CABIMER
Parque Científico y Tecnológico Cartuja 93
41092 Sevilla
España
Tel/Phone:+34 954 468 207
Fax:+34 954 461 664
www.cabimer.es

6.3.4. Análisis funcional de la respuesta inmune

El análisis funcional de la respuesta inmune se realizará siguiendo el procedimiento descrito en el Anexo IX b en:

Dr. David Pozo Perez
Departamento de Bioquímica Médica
y Biología Molecular. Facultad de Medicina
Universidad de Sevilla
Avda. Sánchez Pizjuan, 4
41009.-Sevilla
Tlf: 95 4559851; FAX: 95 4907048

6.3.5. Parámetros de factibilidad

- Variables clínicas
  - **Número de Brotes** (definidos de acuerdo con McDonald)
  - **Progresión** (escalas EDSS y MSFC)

- Variables paraclínicas
  - **Resonancia magnética:** El estudio de RM se realizará en un equipo de alto campo 1.5T, utilizando las siguientes secuencias diferentes para el análisis de las lesiones (véase Anexo III):

    - Secuencias de localización
    - Secuencia transversal en densidad de protones/T2 (DP/T2)
    - Secuencia transversal FLAIR
    - Secuencia spin-eco transversal potenciada en T1
    - Secuencia spin-eco transversal potenciada en T1 con Gadolinio
    - Secuencia difusión isotrópica
    - Transferencia de magnetización
    - Espectroscopia de protón Hidrógeno

- **Potenciales evocados** (visuales, acústicos, somatosensitivos, cognitivos, motores, escala EPAS). Los potenciales evocados se obtendrán con un electromiógrafo con software para potenciales evocados con amplificador de cuatro canales. Se utilizarán electrodos de superficie para la recogida de los potenciales. La metodología aplicada será la de las guías publicadas (Deuschl G, Eisen A. Recommendations for the practice of clinical neurophysiology: Guidelines of the International Federation of Clinical Neurophysiology. Electroencephalogr. Clin. Neurophysiol. 1999; 52 (Suppl):192-211) y se compararán con los valores de normalidad obtenidos en nuestros laboratorios (véase Anexo IV).

- **Tomografía óptica de coherencia**. La prueba se realizará utilizando un aparato de TCO I, realizando dos cortes en cada paciente, uno en el eje vertical y otro en el horizontal, de 5 mm. Centrados en la fóvea. Se indicará al paciente que fije la vista en la luz interna en caso de baja agudeza visual. El "scan" se localiza sobre la zona foveal visualizada a través del monitor ayudado por la fijación externa auxiliar del ojo contralateral. Las imágenes serán normalizadas y alineadas utilizando el software del aparato. Se realizará medición de la presión intraocular y dilatación pupilar farmacológica previa realización de la prueba.

- **Pruebas neuropsicológicas,** (Batería BRB y escala de inteligencia de Wechsler para adultos). Las pruebas neuropsicológicas se administran por escrito a los pacientes que las realizarán en presencia de un neuropsicólogo que explicará la prueba y utilizará las herramientas de apoyo imprescindibles, como un magnetófono en el PASAT (Véase Anexo V).

- **Escalas de calidad de vida** (escalas SF-36, Euroqol-5D, MusiQol). Serán completadas por los pacientes, bajo supervisión de un neuropsicólogo (véase Anexo VI), o por un familiar o una persona afín al paciente que transmita en el cuestionario de forma literal las impresiones del paciente

  - Los brotes se evaluarán en todas las visitas que realicen los pacientes a excepción de en la visita basal.
  - La escala EDSS se evaluará en la visita de preinclusión, en la visita del primer mes y en todas las sucesivas.
  - La escala MSFC se evaluará en la visita de preinclusión, en la visita de los 90 días y en todas las sucesivas.
  - La resonancia magnética se llevará a cabo en la visita de preinclusión, a los 6 meses y a los 12 meses.
  - Los potenciales evocados se evaluarán en la visita de preinclusión, a los 6 meses y a los 12 meses.
  - La tomografía se realizará en la visita de preinclusión, a los 6 meses y a los 12 meses.
  - Las pruebas neuropsicológicas se evaluarán en la visita de preinclusión, a los 6 meses y a los 12 meses.
  - Los test de calidad de vida se completarán en la visita de preinclusión, a los 6 meses y a los 12 meses.

6.3.6. Parámetros de seguridad:

Se evaluará la aparición, intensidad y relación con el medicamento en estudio de acontecimientos adversos y acontecimientos adversos graves en todas las visitas del estudio. Además de la farmacovigilancia se tendrán en cuenta los siguientes parámetros como medidas de seguridad:

- Analítica general
- Analítica inmunológica
- ECG
- Radiografía de tórax
- PFR
- LCR

Todas estas variables se medirán en la visita de preinclusión, la analítica general se repetirá en todas las visitas del estudio a excepción de la visita del día 1 y la visita de la primera semana. La analítica inmunológica se repetirá en la visita de los 90 días y al primer año de seguimiento.

El ECG, la radiografía de tórax y el PFR se realizarán además de en la visita de preinclusión, a los 90 días y a los 12 meses. El LCR se llevará a cabo en la visita de preinclusión, y a los 12 meses de seguimiento.

6.4. ASIGNACIÓN DEL TRATAMIENTO

La asignación a cada uno de los tres brazos de tratamiento del estudio se realizará de forma aleatoria y antes de la visita basal del estudio. La aleatorización se realizará en bloques de tres pacientes atendiendo a los brazos del estudio. Los brazos son:

- **Brazo 1 - Grupo Control:** 10 pacientes que recibirán como medicación de estudio placebo en las mismas condiciones que los grupos experimentales.
- **Brazo 2 - Grupo Experimental 1:** 10 pacientes que recibirán como medicación de estudio una suspensión de células troncales mesenquimales en una única dosis de $1 \cdot 10^6$ células/kg de paciente por vía i.v en bolo, tras haber cogido una vía periférica que se mantendrá con suero salino fisiológico, en la visita basal del estudio.
- **Brazo 3 - Grupo Experimental 2:** 10 pacientes que recibirán como medicación de estudio una suspensión de células troncales mesenquimales en una única dosis de $4 \cdot 10^6$ células/kg de paciente por vía i.v en bolo, tras haber cogido una vía periférica que se mantendrá con suero salino fisiológico, en la visita basal del estudio.

6.5. TÉCNICAS DE ENMASCARAMIENTO

Se trata de un estudio triple ciego, con respecto al tratamiento administrado a los pacientes; donde ni el paciente ni los investigadores conocen si el paciente recibe placebo o tratamiento, ni la dosis administrada. El responsable de analizar los datos estadísticos también desconoce la dosis de cada resultado.

Para permitir la evaluación sin sesgos de la medicación se ha puesto en práctica un estudio evaluado a través de un grupo control, de tres ramas, triple ciego y aleatorizado.

Se usarán los siguientes métodos para mantener el ciego para pacientes, investigadores y para las personas encargadas del análisis estadístico de resultados:

- Extracción de tejido adiposo tanto al grupo control como a los grupos experimentales.

- Codificación oculta de células autólogas trasplantadas o placebo tanto para investigadores como para pacientes y personas encargadas del análisis estadístico de resultados

- La aleatorización de los pacientes se hará de forma centralizada en el CABIMER atendiendo al tratamiento que haya que administrar a cada paciente (control, grupo experimental 1 y grupo experimental 2). La asignación de cada paciente a cada brazo del estudio se guardará en un sobre perfectamente cerrado e identificado para cada paciente en donde se incluirá, aparte de la codificación, las instrucciones con el procedimiento a seguir en el caso de que sea necesario desvelar el ciego. Estos sobres se adjuntarán con la medicación del estudio y se guardarán en el archivo del investigador en el apartado correspondiente a medicación del estudio.

- Acceso restringido al conocimiento respecto a la medicación del estudio (células mesenquimales autólogas o placebo) prescrita a un paciente específico. Los investigadores tendrán acceso, al nombre de las medicaciones enmascaradas administradas solamente a los pacientes bajo su responsabilidad. Los investigadores y otras personas autorizadas no abrirán el código de tratamiento a no ser que sea absolutamente necesario distinguir el tipo de tratamiento recibido por un paciente dado con el fin de elegir entre opciones terapéuticas cruciales.

- Ninguno de los comités supervisores tendrá acceso a la lista de códigos de tratamientos asignados a los pacientes, excepto las personas autorizadas, que serán responsables de supervisar todos los aspectos de seguridad del estudio. La documentación no enmascarada permanecerá de forma confidencial y no estará disponible para nadie fuera del ensayo.

Los tratamientos del estudio se asignarán usando una aleatorización central equilibrada, secuencial, con un factor de estratificación: centro. Para alcanzar un equilibrio aproximado de tratamiento dentro de cada estrato se usará una pauta de aleatorización en bloques de 3 pacientes con asignación 2:1. A cada paciente que entre en el estudio le corresponderá un número de paciente y según dicho listado pertenecerá al grupo control o a cada uno de los dos grupos experimentales del estudio.

7. SELECCIÓN DE SUJETOS

7.1. POBLACIÓN A SER ESTUDIADA

Pacientes con EM secundariamente progresiva que no responden adecuadamente a los tratamientos registrados.

7.2. CRITERIOS DE INCLUSIÓN

1. Pacientes de ambos sexos de edad ≥ 18 años.
2. Pacientes diagnosticados de Esclerosis Múltiple (EM) clínicamente definida (criterios de Poser y McDonald)
3. Pacientes con EM secundariamente progresiva, con EDSS ≥ 5,5 y ≤ 9
4. Pacientes con fracaso terapéutico definido por:
   - 4.1. No respondedor a las terapias empleadas (inmunomoduladores-inmunosupresores) y
   - 4.2. que presenten actividad en forma de 1 brote en el último año o progresión de 0,5 puntos en la escala EDSS
5. Pacientes que no hayan presentado brotes en el mes previo a la inclusión, ni recibido tratamiento esteroideo en el mes previo a la inclusión.
6. Pacientes que otorguen su consentimiento por escrito para la participación en el estudio.

Los pacientes incluidos en el estudio deberán cumplir todos los criterios de inclusión.

7.3. CRITERIOS DE EXCLUSIÓN

1. Existencia de procesos intercurrentes que puedan previsiblemente alterar las medidas de las variables previstas: Patología hepática, renal, cardíaca, etc y patología psiquiátrica u ocular (glaucoma-se realizará medición de presión intraocular previa a la realización de la TCO, cataratas).

2. Portador de marcapasos o implantes metálicos (p.ej. prótesis) que impida realización de RM.

3. Negativa a firmar el consentimiento informado por escrito.

4. Imposibilidad prevista para obtener una biopsia de al menos 30 g de tejido adiposo limpio.

5. Infección por VIH, Hepatitis B o Hepatitis C.

6. Haber padecido o sufrir actualmente una neoplasia maligna.

7. Haber estado en tratamiento con cualquier fármaco en investigación o haberse sometido a cualquier procedimiento experimental en los 3 meses previos al inicio del estudio.

8. Índice de masa corporal > 40 Kg/m^2.

9. Paciente que hayan sido tratados con medicación concomitante prohibida durante el mes anterior a la inclusión en el estudio.

10. Embarazo o lactancia

11. Paciente que haya participado en los últimos 3 meses en otro ensayo clínico.

Los pacientes incluidos en el estudio no deberán cumplir ninguno de los criterios de exclusión.

7.4. CRITERIOS DE RETIRADA Y ANÁLISIS PREVISTO DE LAS RETIRADAS Y ABANDONOS

Los pacientes interrumpirán el ensayo clínico si ocurre al menos una de las siguientes situaciones:

1. Se obtiene una cantidad insuficiente (<30 g) de tejido adiposo.

2. En caso de no alcanzar la concentración final óptima de la suspensión celular el investigador decidirá sobre su administración, aunque el paciente quedará excluido del análisis de factibilidad/seguridad, previa puesta en conocimiento del paciente y autorización del mismo para dicho procedimiento.

3. Presencia de acontecimiento adverso grave anterior a la infusión de las células mesenquimales.

4. Condiciones clínicas del paciente que impidan su continuidad.

5. Toxicidad grado IV en la escala de efectos adversos de la OMS (Véase Anexo VII)

6. Cualquier criterio del Comité de Seguridad que indique la finalización del estudio por motivos de seguridad.

Además, los sujetos pueden ser retirados del estudio por las siguientes razones:

- Cuando el paciente no coopere o no cumpla los requerimientos del estudio.
- Cuando el investigador considere que la salud del paciente está comprometida debido a reacciones adversas, enfermedades concomitantes o cualquier otra circunstancia que se presente durante el estudio*.
- Acontecimiento(s) adverso(s)*.
- Valor(es) anómalo(s) de laboratorio*.
- Resultado(s) anómalo(s) de los procedimientos de las pruebas*.
- Violación del protocolo.
- Retirada del consentimiento por parte del paciente.
- Pérdida de seguimiento del paciente.

*Una vez que el paciente ha recibido la administración del producto en investigación (se administra una única dosis), la retirada del estudio no le aportará ningún beneficio en cuanto a su seguridad, por lo que se recomendará su continuidad en el mismo.

Cuando el investigador deje de tener noticias de un paciente, debe hacer todos los intentos de ponerse en contacto con él/ella (a no ser que el paciente haya expresado claramente su deseo de que no se pongan en contacto con él) para obtener la fecha en que se interrumpió el seguimiento del paciente, para establecer el motivo de la interrupción, para pedir al paciente que reanude los procedimientos del estudio o para que acuda al menos a una última visita y para sugerirle que facilite los detalles de contacto del médico que seguirá su caso.

Si fallasen todos los intentos de ponerse en contacto con el paciente se pedirá que todas las acciones puestas en práctica se documenten en la historia clínica, y si no pueden obtenerse los datos claves del estudio antes de la visita final, el investigador declarará entonces al paciente como “perdido para seguimiento”.

En caso de interrupción prematura del estudio, el investigador debe anotar en el CRD el motivo(s) de la interrupción. Si se da más de un motivo, el investigador debe dar el motivo principal.

Se pedirá al paciente que acuda a las visitas programadas hasta que se declare el fin del estudio incluso si se interrumpe permanentemente el tratamiento del estudio, con el fin de permitir al investigador recoger el mayor número de datos, incluyendo parámetros de seguridad.

El paciente tiene derecho de discontinuar el estudio en el momento en que lo desee y cualquier paciente puede ser retirado del estudio por cualquier motivo beneficioso para su bienestar.

Según la buena práctica clínica, a todos los pacientes que abandonen prematuramente el estudio se les tendrá que recomendar un tratamiento alternativo. Si la retirada es debida a un acontecimiento adverso significativo, los pacientes serán controlados por el investigador hasta la finalización adecuada, es decir, hasta que desaparezca el acontecimiento adverso o hasta que se determine que es permanente.

7.5. INTERRUPCIÓN DEL ENSAYO

El estudio podrá interrumpirse ante cualquier acontecimiento adverso grave relacionado con el tratamiento o si se cumplen cualquiera de las siguientes circunstancias:

1. Toxicidad grave relacionada con la infusión en 1/3 ó 2/30 pacientes.
2. Infecciones graves > 2/30 pacientes.
3. Mortalidad relacionada con el procedimiento ≥ 1 paciente.
4. Falta de inclusión de un mínimo de 3 pacientes en los primeros 12 meses desde su aprobación y distribución.

7.6. NÚMERO PREVISTO DE PACIENTES

Se incluirán un total de 30 pacientes (10 pacientes por cada brazo del estudio) evaluables para seguridad y factibilidad.

7.7. IDENTIFICACIÓN DE LOS SUJETOS

Los pacientes serán identificados con un código numérico de identificación secuencial, el cual será asignado a cada paciente según orden correlativo de inclusión cuando otorguen su consentimiento informado. En el caso de que un paciente sea incluido en el estudio, es decir firme el consentimiento informado, y una vez realizados los procedimientos de la visita de selección se considere no apto para continuar en el estudio o bien el paciente retire su consentimiento, el número que tenía asignado ese paciente no podrá ser reutilizado para otro paciente.

El promotor solo podrá identificar a los sujetos por el número que les ha sido asignado, su fecha de nacimiento y su sexo. El investigador deberá mantener un registro con los nombres de los pacientes y el número de identificación asignado.

8. DESCRIPCIÓN DEL TRATAMIENTO

8.1. EXTRACCIÓN DE TEJIDO APIDOSO

El procedimiento a seguir para la extracción de la muestra será el utilizado en la práctica habitual para este tipo de cirugía, aún así se recomienda seguir el siguiente procedimiento:

1.- Antes de proceder a la extracción de la muestra, se deberá comprobar que se dispone del consentimiento informado para realizar la toma de la muestra, previamente firmado por el paciente en el período de preinclusión

2.- En quirófano reglado se procederá con los siguientes pasos:

A) Lavado de la zona abdominal, región periumbilical.
B) Pintar con líquido antiséptico (povidona yodada).
C) Infiltrar la zona con anestésico local, tipo mepivacaina al 3%, sin vasoconstrictor, utilizando aguja gold, nº 27g biselada
D) Resección de tejido adiposo, incluyendo piel, en zonas arriba mencionadas, ya sea en una única o varias piezas, de modo que la muestra total sea de al menos 30 g.
E) Introducción de las muestras en recipientes estériles, con suero fisiológico.
F) Cierre del panículo adiposo con puntos de Dexon y piel con puntos de seda con aguja TB 15.
G) Envío de muestras al laboratorio de CABIMER en condiciones de refrigeración (entre 2-8ºC) en un kit de transporte con un registrador de temperatura continuo.

Una vez remitidas las muestras al CABIMER, se procesarán las muestras para conseguir el producto final, células troncales mesenquimales de tejido adiposo autólogo tanto en los grupos experimentales como en el grupo control, si bien a este último grupo no se le administrarán hasta la finalización del estudio y si los resultados de factibilidad y seguridad lo permitieran, previa solicitud de extensión del estudio.

8.2. PREPARACIÓN DEL PRODUCTO EN INVESTIGACIÓN

Desde la extracción del tejido adiposo hasta su entrega en CABIMER no podrán transcurrir más de 24h desde la fecha y hora de la toma de la muestra. La muestra de tejido graso irá contenida en un frasco cerrado estéril de 100 ml con suero salino, será transportada a CABIMER por personal debidamente formado y autorizado y deberá ir correctamente identificada con el número de paciente, código de protocolo, promotor, tipo de muestra y datos que solicite CABIMER. La muestra de tejido graso hasta su entrega a CABIMER deberá mantenerse entre 2-8ºC. La cantidad de tejido graso necesaria para la expansión de las células mesenquimales será ≥ 30g.

Tras la preparación de la muestra de tejido adiposo, las células obtenidas, se expandirán en condiciones GMP, en un laboratorio destinado a tal efecto. Durante su expansión las células no sufrirán más que un determinado número de pases (7 pases o expansiones como máximo). Una vez que se obtenga el número de células indicado para el paciente que se está tratando, se procederá al envasado de las células en una jeringa precargada. Además se les realizará un fenotipaje por citometría de flujo, para conocer la expresión de los marcadores de las células, durante su procesamiento y una vez se vayan a envasar. También se tomará una muestra para posterior realización de cariotipo y estudio de estabilidad cromosómica.

Una vez finalice la expansión de las células y se tengan las jeringas con las células cargadas en CABIMER, serán enviadas a los centros participantes, por personal debidamente formado y autorizado y debidamente etiquetadas, tal y como figura en el anexo VIII del protocolo. Las jeringas irán en bolsas térmicas de transporte con 2 acumuladores de frío (previamente congelados durante al menos 24h a –20ºC). El contenedor para el transporte de las muestras biológicas deberá mantenerse refrigerado (2-8ºC) hasta el momento de la implantación al paciente. Una vez las células lleguen al hospital, deberá ser recepcionado en el servicio de farmacia. El tiempo máximo transcurrido desde la preparación a la entrega no deberá ser superior a 24h.

8.3. PRESENTACIÓN DEL PRODUCTO EN INVESTIGACIÓN

Producto en investigación: células mesenquimales de tejido adiposo autólogas.

Forma farmacéutica: jeringas que contienen una suspensión de células mesenquimales de tejido adiposo autólogas o placebo.

Composición de la suspensión de células mesenquimales autólogas de tejido adiposo

- Sustancia activa: células troncales mesenquimales de tejido adiposo
- Excipientes:
    - Solución de Ringer lactada:
        - Cloruro cálcico dihidratado (2,7 mg/100ml)
        - Lactato sódico (320 mg/100ml)
        - Cloruro potásico (40 mg/100ml)
        - Cloruro sódico (600 mg/100ml)
    - 2,5% de glucosa
    - 1% albúmina.

Composición de placebo

- Solución de Ringer lactada:
    - Cloruro cálcico dihidratado (2,7 mg/100ml)
    - Lactato sódico (320 mg/100ml)
    - Cloruro potásico (40 mg/100ml)
    - Cloruro sódico (600 mg/100ml)
- 2,5% de glucosa
- 1% albúmina.

Vía de administración: intravenosa

Pauta: una única administración.

Cada paciente recibirá una de las siguientes dosis de células mesenquimales de tejido adiposo:

1) $1x10^6$ células / kg de peso paciente

2) $4x10^6$ células / kg de peso paciente
3) placebo

Fabricante del producto en investigación: CABIMER

8.4. IDENTIFICACIÓN DEL PRODUCTO EN INVESTIGACIÓN

La identificación y etiqueta del producto en investigación se detalla en el Anexo VIII.

8.5. ADMINISTRACIÓN DE LAS CÉLULAS MESENQUIMALES DE TEJIDO ADIPOSO

Procedimiento canalización venosa para la administración de células mesenquimales de tejido adiposo: Idealmente se canulará la vena radial con un catéter Abocath de 14-16G". Se conectará a una llave de 3 pasos, sistema de suero y suero fisiológico (SF) de 500cc. Con el SF se realizará un lavado de la vía para comprobar la permeabilidad de la misma y se procederá a la administración de la suspensión de las células mesenquimales en bolo de infusión lenta a través de la llave de 3 pasos. Posteriormente se procederá a lavar la vía con 250cc de SF.

8.6. TRATAMIENTOS CONCOMITANTES PERMITIDOS Y PROHIBIDOS

8.6.1. TRATAMIENTOS CONCOMITANTES PERMITIDOS

Se permitirá la administración de corticosteroides para tratar las recidivas a discreción del médico encargado del tratamiento. Los centros participantes aplicarán sus prácticas habituales.

A discreción del investigador, puede administrarse cualquier medicamento, salvo los excluidos por el protocolo, que se considere necesario para el bienestar del paciente y que no interfiera con el producto en investigación.

8.6.2. TRATAMIENTOS CONCOMITANTES PROHIBIDOS

Administración de antibióticos, fármacos citostáticos, citotóxicos, inmunomoduladores y/o inmunosupresores

Estará prohibido el tratamiento con medicamentos esteroides un mes antes de la inclusión del paciente en el estudio

8.6.3. TRATAMIENTOS DE LAS COMPLICACIONES

Las complicaciones derivadas del procedimiento serán tratadas de forma estándar, y siempre a criterio del investigador. Deberán ser registradas en el cuaderno de recogida de datos como acontecimientos adversos y los tratamientos que requieran como medicación concomitante.

9. DESARROLLO DEL ENSAYO Y EVALUACIÓN DE LA RESPUESTA

9.1. EVALUACIÓN DE LA RESPUESTA

9.1.1. Variables clínicas

- Sexo: variable cualitativa (varón, mujer)
- Edad: variable cuantitativa (años)
- Edad de comienzo de la enfermedad: variable cuantitativa (años)
- Síntomas de comienzo de la EM: variable cualitativa (piramidal, cerebeloso, sensitivo, tronco cerebral, visual, esfinteriano, mental).
- Tiempo de evolución de la enfermedad: variable cuantitativa (años)
- Disfunción neurológica: variable cuantitativa medida:

  - Puntuación en la escala ampliada de Kurtzke (EDSS). (Kurtzke JF. Rating neurological impairment in multiple sclerosis: an expanded disability scale status. Neurology 1983;33:1444-1452).

  - Puntuación de la escala Multiple Sclerosis Functional Composite (MSFC) (Fisher JS, Rudick RA, Cutter GR, Reingold SC. For the National MS Society Clinical Outcomes Assessment Task Force. The multiple sclerosis functional composite measure (MSFC): an integrated approach to MS clinical outcome assessment. Mult Scler 1999;5:244-250).

- **Brotes:** variable cuantitativa (número de brotes)

- **Progresión de la discapacidad medida por las escalas EDSS y MSFC**: variable cuantitativa

- **Analítica general**: variables cuantitativas

- **Variables de analítica inmunológicas (Véase Anexo IX)**

  - Subpoblaciones celulares (linfocitos T CD4+, Linfocitos T CD8+, Linfocitos NK y monocitos). Variable cuantitativa (nº, %).
  - IFN-γ, TNF-ß, IL-2, IL-12, IL-4, IL-5, IL-10, IL-17A, IL-23 e IL-6: Variable cuantitativa.

- **LCR** (Véase Anexo X)

  - Variable cuantitativa
  - Variable cualitativa (presencia/ausencia de bandas oligoclonales) y secreción intratecal de IgG.

9.1.2. Acontecimientos adversos, escala de la OMS:

- Variable cuantitativa (número de casos)
- Variable cualitativa (gravedad)

9.1.3. Variables neurofisiológicas (potenciales evocados)

- Potenciales evocados visuales: latencia P100: variable cuantitativa (ms); amplitud N75-P100: Variable cuantitativa (μV)
- Potenciales evocados acústicos: intervalo I-V: variable cuantitativa (ms); amplitud I/V variable cuantitativa (μV).
- Potenciales evocados somatosensitivos n. mediano: latencia N20 : variable cuantitativa (ms); amplitud p14-N20 variable cuantitativa (μV); tiempo de conducción central N13-N20, variable cuantitativa (ms).
- Potenciales evocados somatosensitivos n. tibial: latencia P39, variable cuantitativa (ms); amplitud P30-P39 variable cuantitativa (μV); tiempo de conducción central N22-P39, variable cuantitativa (ms).
- Potenciales evocados motores de miembros superiores e inferiores: tiempo de conducción central al músculo abductor del primer dedo y al tibial anterior, variable cuantitativa (ms).
- Potencial evocado cognitivo P-300: latencia, variable cuantitativa (ms) y amplitud, variable cuantitativa (μV).
- Escala global paramétrica de puntuación generada de la combinación de varios potenciales evocados: EPAS (del inglés "Evoked Potentials Abnormality Store"): para cada modalidad se generará una variable cuantitativa ordinal (0 = normal, 1 = latencia incrementada, 2 = latencia incrementada con cambios morfológicos/disminución de amplitud, 3 = ausencia de algún componente principal del potencial evocado) generada con la suma de ambos lados para los PEV y los PEAT (0-6), y para cada lado para los PES y PEM (0-12). La escala general combinada va de 0 a 36, donde los valores más elevados representan mayor afectación de los potenciales evocados. (Leocani L et al. 2006).

9.1.4. Variables de tomografía óptica de coherencia (TCO)

Estrategia de análisis a realizar con midriasis farmacológica:

I. "Fast macular thickness map": fijación foveal.
II. "10 mm post pole": fijación foveal.
III. "6 mm cross hair scan": fijación foveal.
IV. "Fast RNFL Thickness": fijación nasal al NO.
V. "Fast optic disc": fijación nasal al NO.

i. Variables neuropsicológicas (test neuropsicológicos):

- Batería BRB (Brief Repeatable Battery of Neuropsychological Test in Multiple Sclerosis de Rao), variable cuantitativa medida por la puntuación compuesta en cada uno de los siguientes subtest:
  - Selective reminding test (SRT)

- 10/36 Spatial Recall Test
- Symbol Digit Modalities Test
- Prueba de Evocación Categorial
- Paced Auditory S [PASAT]

- Escala de inteligencia de Wechsler para adultos.WAIS-III,variables cuantitativas.

ii. Variables de resonancia magnética nuclear

- Nº/volumen lesiones en T2, variable cuantitativa (nº, mm^3)
- Nº/volumen lesiones en T1 (agujeros negros), variable cuantitativa
- Nº/volumen lesiones en T1 con Gadolinio (lesiones activas), variable cuantitativa (nº, mm^3)
- Volumen cerebral, variable cuantitativa (mm^3)
- Coeficiente de difusión aparente (DCA), variable cuantitativa
- Transferencia de magnetización, variable cuantitativa
- Espectroscopia por RM, variable cuantitativa

iii. Variables de calidad de vida

- SF-36
- Euroqol-5D
- MusiQol

9.2. DESARROLLO DEL ENSAYO

El procedimiento de administración de las CeTMAd (día 0 de estudio) y el resto de visitas y procedimientos del estudio tendrán lugar en los centros participantes en el presente estudio.

- Vista 1: Selección: La selección de los pacientes candidatos se realizará desde la consulta de neurología de los centros participantes. Esta visita deberá tener lugar como máximo hasta un mes antes de la extracción de CeTMAd, pudiendo tener lugar el mismo día de la obtención de tejido adiposo.

En esta visita, el paciente potencialmente apto para el estudio será informado de los objetivos del estudio y de los requisitos globales. Se obtendrá el consentimiento informado antes de cualquier evaluación específica del estudio. A continuación, se realizará al paciente una evaluación clínica para asegurar el cumplimiento de los criterios de inclusión y exclusión.

En la historia clínica del paciente se deberá registrar como el paciente acepta participar en el ensayo clínico CeTMAd/ICPD/2008, fecha de la firma del consentimiento, número de paciente asignado y promotor del estudio.

Se registrará la fecha de la visita y se recogerán:

Historia Clínica: sobre otras enfermedades concomitantes que puedan presentar los pacientes en el momento de su inclusión en el estudio y tratamiento que esté realizando en ese momento.

Anamnesis. Historia de la enfermedad, incluida la fecha del diagnóstico, la clasificación de la enfermedad, el historial de recidivas, la historia de los tratamientos de la EM y fecha de inicio y retirada/abandono y motivo del mismo.

Exploración general: los datos demográficos (fecha de nacimiento, sexo), signos vitales (tensión arterial, frecuencia cardiaca, frecuencia respiratoria), peso, talla, historia médica y datos sobre los antecedentes personales de los pacientes.

Exploración neurológica incluido el examen de sistemas funcionales, EDSS y MSFC.

Analítica general

Hemograma: Hemograma completo, VS, Tiempo de Protrombina-INR, TTPA.

Bioquímica: glucosa, urea, creatinina, ácido úrico, colesterol total, HDL-colesterol, triglicéridos, proteinas totales, albúmina, Coeficiente albúmina/globulina, bilirrubina total, bilirrubina directa, haptoglobina, ferritina, IgA, IgG, IgM, C3, C4, calcio, Fosforo, sodio, potasio, cloro, hierro, GOT, GPT, Fosfatasa Alcalina, LDH, CPK y PCR de rutina.

Analítica inmunológica (véase Anexo IX a y b) para analisis de citoquinas y análisis funcional de la respuesta inmune

Punción Lumbar para LCR

ECG

RX Tórax

Espirometría Básica con test de difusión (PFR)

Historial de Brotes en los 2 años previos y Potencial Evocados

Espectroscopia por Resonancia Magnética, Transferencia de Magnetización, Tomografía Óptica de Coherencia, Resonancia Magnética convencional

Tests Neuropsicológicos

Calidad de Vida

Una vez obtenidos los resultados de todos los procedimientos arriba mencionados y comprobado que se cumplen todos los criterios de inclusión y ninguno de exclusión, el paciente podrá continuar en el estudio. Si por lo contrario, el paciente no cumple con los criterios de inclusión no podrá continuar en el estudio y deberá registrase en el CRD el motivo de la no selección del paciente (véase Anexo XI)

- **Visita para extracción de tejido adiposo** Esta visita podrá tener lugar hasta aproximadamente un mes de la inclusión del paciente en el estudio o coincidir con la visita de selección. El procedimiento de extracción de tejido adiposo se describe en el punto 8.1

En la historia clínica y en el CRD se recogerán los siguientes datos:

- Fecha de la visita: es la fecha de la extracción.
- Signos vitales (tensión arterial, frecuencia cardiaca), peso.
- Cantidad de tejido adiposo extraído (deberá ser > 30g).
- Hora de la extracción.
- Posibles complicaciones durante la extracción de tejido adiposo. En el caso de que hayan tenido lugar, se deberán registrar en la página de acontecimientos adversos del CRD.

En la historia clínica y en el CRD del paciente se recogerán todos los acontecimientos adversos (AA) y medicaciones concomitantes desde la inclusión del paciente hasta esta visita.

- **Expansión de las células mesenquimales de tejido adiposo**: se realizará en CABIMER. Este proceso tiene una duración aproximada de 2-4 semanas y se describe en el punto 8.2.

En la historia clínica del paciente y en el CRD deberán constar los siguientes datos:

- Fecha de inicio y fecha de finalización de la expansión de las CeTMAd.
- Fecha y hora de caducidad de las células.
- Nº de células obtenidas.
- Nº de jeringas.
- Fenotipo celular.
- Viabilidad (%).
- Control de calidad: ensayo de esterilidad, ensayo de pirógenos y endotoxinas bacterianas y ensayo de micoplasma.
- Incidencias que hayan podido tener lugar durante el proceso de expansión.

Una vez conseguida la expansión de las células mesenquimales se realizará la administración del placebo o de las células mesenquimales al paciente en los centros participantes en el presente ensayo.

-**Visita 2: Basal Día 0,** previa a la administración del placebo o células mesenquimales del tejido adiposo al paciente. Se realizarán los siguientes procedimientos para valorar la situación del paciente y confirmar el cumplimiento de los criterios de inclusión y exclusión. Se realizarán los siguientes procedimientos:

-Signos vitales (tensión arterial, frecuencia cardiaca, frecuencia respiratoria)

-Evaluación de los acontecimientos adversos, medicaciones y procedimientos concomitantes.

-Datos de laboratorio:

1.- Se tomarán muestras de sangre para:

Hemograma: Hemograma completo, VS, Tiempo de Protrombina-INR, TTPA.

Bioquímica: glucosa, urea, creatinina, ácido úrico, colesterol total, HDL-colesterol, triglicéridos, proteinas totales, albúmina, Coeficiente albúmina/globulina, bilirrubina total, bilirrubina directa, haptoglobina, ferritina, IgA, IgG, IgM, C3, C4, calcio, Fosforo, sodio, potasio, cloro, hierro, GOT, GPT, Fosfatasa Alcalina, LDH, CPK y PCR de rutina.

2.- Se realizará un análisis de orina elemental y test de embarazo en orina en mujeres en edad fértil.

- Exploración neurológica

- Administración del placebo o de las células mesenquimales del tejido adiposo al paciente y puede coincidir con la visita del día 0 ó visita Basal. Tendrá lugar aproximadamente entre 2-4 semanas después de la extracción de tejido adiposo, una vez haya finalizado el proceso de expansión de las células.

El procedimiento de administración de las CeTMAd se describe en el punto 8.5.

En la historia clínica del paciente y en el CRD deberán constar los siguientes datos:

- Fecha de la visita.
- Hora de la administración de las CeTMAd.
- Signos vitales (tensión arterial, frecuencia cardiaca, frecuencia respiratoria), peso.
- Extremidad en la que se administran las CeTMAd.
- Nº de CeTMAd prefundidas y nº de jeringas.
- Acceso venoso.
- Nivel de administración de las CeMTAd.
- Complicaciones durante el procedimiento: en caso de producirse se deberán registrar como acontecimientos adversos en la página correspondiente del CRD.

En la historia clínica y en el cuaderno de recogida de datos del paciente se recogerán los AA y medicaciones concomitantes durante el procedimiento de administración de las células mesenquimales y desde la visita previa.

En el caso de que la hospitalización del paciente por la infusión de las CeTMAd se prolongue más de lo esperado, debido a alguna complicación surgida desde su ingreso, se deberá registrar en el CRD como acontecimiento adverso grave (AAG), y notificarlo al monitor del estudio en el plazo de 24h en que haya tenido lugar, por TLF y por Fax mediante el formulario de notificación de AAG (ver punto 10 del protocolo).

- Vista 3: Día +1. Con el fin de analizar la seguridad de la administración, **a las 24horas de la administración del placebo o de las células mesenquimales de tejido adiposo,** Se valorará el estado general del paciente.

En la historia clínica y en el CRD del paciente se recogerán los AA y medicaciones concomitantes desde la visita previa.

-Signos vitales (tensión arterial, frecuencia cardiaca, frecuencia respiratoria).

- Exploración Neurológica.

- Ausencia/presencia de Brotes desde la visita anterior.

- Vista 4: Día +8 (±2 días) después de la administración del placebo o de las células mesenquimales de tejido adiposo se valorará el estado general del paciente.

En la historia clínica y en el CRD del paciente se recogerán los AA y medicaciones concomitantes desde la visita previa.

- Fecha de la visita

- Signos vitales (tensión arterial, frecuencia cardiaca, frecuencia respiratoria)

- Exploración Neurológica

- Ausencia/presencia de Brotes desde la visita anterior

- **Visita 5**: **1 mes (± 3 días) después de la administración del placebo o de las células mesenquimales de tejido adiposo**.

Se evaluará la situación clínica del paciente y se recogerán los siguientes datos y procedimientos:

- Fecha de la visita.
- Signos vitales (tensión arterial, frecuencia cardiaca, frecuencia respiratoria)
- Ausencia/presencia de Brotes desde la visita anterior
- Punción Lumbar para LCR
- Exploración Neurológica incluyendo la escala EDSS

1.- Se tomarán muestras de sangre para:

Hemograma: Hemograma completo, VS, Tiempo de Protrombina-INR, TTPA.

Bioquímica: glucosa, urea, creatinina, ácido úrico, colesterol total, HDL-colesterol, triglicéridos, proteinas totales, albúmina, Coeficiente albúmina/globulina, bilirrubina total, bilirrubina directa, haptoglobina, ferritina, IgA, IgG, IgM, C3, C4, calcio, Fosforo, sodio, potasio, cloro, hierro, GOT, GPT, Fosfatasa Alcalina, LDH, CPK y PCR de rutina.

En la historia clínica y en el CRD del paciente se recogerán los AA y medicaciones concomitantes desde la visita previa.

- **Visita 6**: **2 meses (± 3 días) después de la administración del placebo o de las células mesenquimales de tejido adiposo**.

Se evaluará la situación clínica del paciente y se recogerán los siguientes datos y procedimientos:

- Fecha de la visita.
- Signos vitales (tensión arterial, frecuencia cardiaca, frecuencia respiratoria)
- Ausencia/presencia de Brotes desde la visita anterior
- Análisis inmunológico para análisis funcional de la respuesta inmune (véase Anexo IXb)
- Exploración Neurológica incluyendo la escala EDSS

1.- Se tomarán muestras de sangre para:

Hemograma: Hemograma completo, VS, Tiempo de Protrombina-INR, TTPA.

Bioquímica: glucosa, urea, creatinina, ácido úrico, colesterol total, HDL-colesterol, triglicéridos, proteinas totales, albúmina, Coeficiente albúmina/globulina, bilirrubina total, bilirrubina directa, haptoglobina, ferritina, IgA, IgG, IgM, C3, C4, calcio, Fosforo, sodio, potasio, cloro, hierro, GOT, GPT, Fosfatasa Alcalina, LDH, CPK y PCR de rutina.

En la historia clínica y en el CRD del paciente se recogerán los AA y medicaciones concomitantes desde la visita previa.

Visita 7: tendrá lugar a los 3 meses (± 7 días) de la administración del placebo o de las células mesenquimales de tejido adiposo.

Se evaluará la situación clínica del paciente y se recogerán los siguientes datos y procedimientos:

- Fecha de la visita.
- Signos vitales (tensión arterial, frecuencia cardiaca, frecuencia respiratoria), peso
- Evaluación de los acontecimientos adversos, mediaciones y procedimientos concomitantes.
- Examen y evaluación neurológica incluyendo el examen de sistemas funcionales EDSS y MSFC

- Presencia/ausencia de brotes

- ECG

- Rx Torax

- Espirometría básica con test de difusión.

- Análisis inmunológico para análisis de citocinas (véase Anexo IX a y b)

- Datos de laboratorio:

1.- Se tomarán muestras de sangre para:

Hemograma: Hemograma completo, VS, Tiempo de Protrombina-INR, TTPA.

Bioquímica: glucosa, urea, creatinina, ácido úrico, colesterol total, HDL-colesterol, triglicéridos, proteinas totales, albúmina, Coeficiente albúmina/globulina, bilirrubina total, bilirrubina directa, haptoglobina, ferritina, IgA, IgG, IgM, C3, C4, calcio, Fosforo, sodio, potasio, cloro, hierro, GOT, GPT, Fosfatasa Alcalina, LDH, CPK y PCR de rutina.

2.- Se realizará una analítica inmunológica

En la historia clínica y en el CRD del paciente se recogerán los AA y medicaciones concomitantes desde la visita previa.

- **Visita 8: tendrá lugar a los 6 meses (± 7 días) de la administración del placebo o de las células mesenquimales de tejido adiposo.**

Se evaluará la situación clínica del paciente y se recogerán los siguientes datos y procedimientos:

- Fecha de la visita.

- Signos vitales (tensión arterial, frecuencia cardiaca), peso.

- Evaluación de los acontecimientos adversos, mediaciones y procedimientos concomitantes.

-Examen y evaluación neurológica, incluido el examen de sistemas funcionales de EDSS y MSFC.

- Presencia/ausencia de brotes

-Datos de laboratorio:

1.- Se tomarán muestras de sangre para:

Hemograma: Hemograma completo, VS, Tiempo de Protrombina-INR, TTPA.

Bioquímica: glucosa, urea, creatinina, ácido úrico, colesterol total, HDL-colesterol, triglicéridos, proteinas totales, albúmina, Coeficiente albúmina/globulina, bilirrubina total, bilirrubina directa, haptoglobina, ferritina, IgA, IgG, IgM, C3, C4, calcio, Fosforo, sodio, potasio, cloro, hierro, GOT, GPT, Fosfatasa Alcalina, LDH, CPK y PCR de rutina.

- Tomografía Óptica de Coherencia (TOC)
- Análisis inmunológico para análisis de citocinas y funcional de la respuesta inmune (véase Anexo IX a y b)
- RMN, TM y ERM
- Potenciales evocados
- Cuestionarios de calidad de vida
- Test neuropsicológicos

En la historia clínica y en el CRD del paciente se recogerán los AA y medicaciones concomitantes desde la visita previa.

- **Visita 9: tendrá lugar a los 9 meses (± 7 días) de la administración del placebo o de las células mesenquimales de tejido adiposo.**

Se evaluará la situación clínica del paciente y se recogerán los siguientes datos y procedimientos:

- Fecha de la visita.
- Signos vitales (tensión arterial, frecuencia cardiaca), peso.
- Evaluación de los acontecimientos adversos, mediaciones y procedimientos concomitantes.
- Examen y evaluación neurológica, incluido el examen de sistemas funcionales de EDSS y MSFC
- Presencia/ausencia de Brotes desde la visita anterior.
- Datos de laboratorio:

1.- Se tomarán muestras de sangre para:

Hemograma: Hemograma completo, VS, Tiempo de Protrombina-INR, TTPA.

Bioquímica: glucosa, urea, creatinina, ácido úrico, colesterol total, HDL-colesterol, triglicéridos, proteinas totales, albúmina, Coeficiente albúmina/globulina, bilirrubina total, bilirrubina directa, haptoglobina, ferritina, IgA, IgG, IgM, C3, C4, calcio, Fosforo, sodio, potasio, cloro, hierro, GOT, GPT, Fosfatasa Alcalina, LDH, CPK y PCR de rutina.

En la historia clínica y en el CRD se recogerán los AA y medicaciones concomitantes desde la visita previa.

- **Visita 10: tendrá lugar a los 12 meses (± 7 días) de la administración del placebo o de las células mesenquimales de tejido adiposo.**

Se evaluará la situación clínica del paciente y se recogerán los siguientes datos y procedimientos:

-Signos vitales (tensión arterial, frecuencia cardiaca, frecuencia respiratoria),

-Evaluación de los acontecimientos adversos, mediaciones y procedimientos concomitantes.

-Examen y evaluación neurológica, incluido el examen de sistemas funcionales de EDSS y MSFC.

-Presencia/Ausencia de Brotes desde la visita anterior

-Datos de laboratorio:

1.- Se tomarán muestras de sangre para:

Hemograma: Hemograma completo, VS, Tiempo de Protrombina-INR, TTPA.

Bioquímica: glucosa, urea, creatinina, ácido úrico, colesterol total, HDL-colesterol, triglicéridos, proteinas totales, albúmina, Coeficiente albúmina/globulina, bilirrubina total, bilirrubina directa, haptoglobina, ferritina, IgA, IgG, IgM, C3, C4, calcio, Fosforo, sodio, potasio, cloro, hierro, GOT, GPT, Fosfatasa Alcalina, LDH, CPK y PCR de rutina.

2.- Análisis inmunológico para análisis de citocinas y funcional de la respuesta inmune (véase Anexo IX a y b)

-Test neuropsicológicos

-Test de calidad de vida

-Tomografía Óptica de Coherencia

-Potenciales evocados

-RMN, ERM, TM

-ECG

-Rx Tórax

-Espirometría básica con Test de difusión

En la historia clínica y en el cuaderno de recogida de datos del paciente se recogerán los AA y medicaciones concomitantes desde la visita previa.

9.3. DISTRIBUCIÓN DE TAREAS

- Estudios clínicos: Los neurólogos de las Unidades de Esclerosis Múltiple de los Hospitales participantes serán los encargados de evaluar clínicamente a los pacientes a incluir en el estudio.

- Analítica general: se realizará en los laboratorios centrales de los Hospitales participantes.

- Analítica inmunológica: se realizará estudio de las subpoblaciones celulares y un perfil de citoquinas pro y antiinflamatorias, así como la caracterización de los perfiles de expresión génica, metabolómica y de matriz de antígenos en los laboratorios clínicos y de investigación de los Hospitales y centros de investigación participantes.

- Analítica del LCR: se realizará estudio de la respuesta inmune, perfil metabolómico y de expresión en células y líquido procedentes de la punciones lumbares. Se realizará en los laboratorios clínicos y de investigación de los Hospitales y centros de investigación participantes. Este procedimiento se realizará en las visitas de selección y en la del mes 1. Dependiendo de los resultados obtenidos en la analítica del LCR realizada en la visita del mes 1 se podrán realizar hasta un máximo de dos analíticas de LCR adicionales a lo largo del estudio.

- Estudios de neurofisiología clínica-potenciales evocados: Se realizarán en los Servicios de Neurofisiología de los Hospitales participantes.

- Estudios de RM: Se realizarán en los Servicios de Radiología de los Hospitales participantes.

- Estudios de TOC: Se realizarán en los Servicios de Oftalmología de los Hospitales participantes.

- Estudios neuropsicológicos: Los neuropsicólogos de las Unidades de Esclerosis Múltiple de los Hospitales participantes serán los encargados de realizar los tests neuropsicológicos a los sujetos a incluir en el estudio.

- Estudios de calidad de vida: serán realizados por los neuropsicólogos de las Unidades de Esclerosis Múltiple de los Hospitales participantes.

10. ACONTECIMIENTOS ADVERSOS

Se registrarán todos los acontecimientos adversos (AA) en los cuadernos de recogida de datos desde el momento que el paciente firme su consentimiento informado. El investigador decidirá si estos acontecimientos están relacionados con la terapia celular o tratamiento recibido (no relacionado, improbable, posible, probable, seguro, no valorable) y su decisión será registrada en las hojas para todos los acontecimientos adversos. No se considerarán como efectos secundarios o toxicidad los acontecimientos adversos no relacionados con la terapia celular (es decir, comunicados como no relacionados o de relación improbable); estos serán informados por separado en el análisis.

10.1. COMITÉ DE SEGURIDAD

Para minimizar la posibilidad de exponer a los pacientes del estudio a un riesgo inhabitual se instaurará un Comité de Seguridad el cual revisará de forma continuada la información de seguridad y factibilidad que se genere en el estudio.

El Comité revisará los datos de factibilidad y seguridad y un análisis de riesgo/beneficio. El propósito de este Comité es asesorar sobre asuntos graves de seguridad y pérdidas de factibilidad. El Comité puede solicitar reuniones adicionales para discutir con el Promotor asuntos relativos a seguridad y/o factibilidad. El Comité puede parar el estudio en base a la revisión de los resultados en análisis intermedios. Igualmente puede solicitar análisis intermedios adicionales cuando los resultados obtenidos hasta ese momento de seguridad y factibilidad así lo consideren.

10.2. DEFINICIONES

Un acontecimiento adverso se define como cualquier episodio o experiencia médica desfavorable en un paciente o sujeto de ensayo clínico que recibe un tratamiento experimental, en este estudio la administración de las células mesenquimales de tejido adiposo, sin importar la dosis o relación causal. Esto puede incluir cualquier signo (como una erupción cutánea o la hepatomegalia) o síntoma (como las náuseas o el dolor torácico) desfavorable o no intencional, un hallazgo anormal de laboratorio (incluyendo análisis de sangre, radiografías o escáneres), una enfermedad temporalmente asociada al uso de la terapia celular del estudio o un empeoramiento de un trastorno preexistente.

Además, todo acontecimiento asociado a una sobredosis del producto, también será considerado como acontecimiento adverso.

Un trastorno persistente es una afección clínica (incluido un trastorno que está siendo tratado) que se diagnosticó antes de que el sujeto firmara el consentimiento informado y que está documentado en la historia médica del sujeto.

Un Acontecimiento Adverso Grave (AAG) se define como cualquier experiencia no deseada que afecta a un paciente, se considere o no relacionada con el tratamiento del protocolo. Los acontecimientos adversos graves son aquellos que dan lugar a:

- La muerte.
- Un acontecimiento con riesgo para la vida (es decir, hubo un riesgo de muerte inmediata del paciente en el momento en que se observó la reacción).
- Hospitalización o prolongación de una hospitalización.
- Discapacidad/incapacidad persistente o significativa.
- Una anomalía congénita o un defecto de nacimiento.
- Cualquier otra condición médica importante (es decir, reacciones adversas importantes que no suponen un riesgo inmediato para la vida y no dan lugar a la muerte o a la hospitalización pero que pueden poner en peligro al paciente o pueden necesitar intervención para prevenir otro de los resultados listados arriba) según el criterio médico apropiado.

Se define la muerte tóxica como la muerte secundaria a la toxicidad. Esto debe especificarse en el formulario del informe de muerte: la causa de la muerte debe figurar como "toxicidad". La valoración de las muertes tóxicas es independiente de la valoración de la respuesta (los pacientes pueden morir por toxicidad después de una valoración completa de la respuesta al tratamiento).

La hospitalización se define como el ingreso oficial en un hospital. La hospitalización y la prolongación de una hospitalización constituyen criterios de gravedad de un AA; sin embargo, no se consideran por si mismas un AAG. Si no existe un AA, el investigador no debe notificar como AAG la hospitalización o la prolongación de una hospitalización. Éste es el caso en las siguientes situaciones:

- Se necesita la hospitalización o la prolongación de una hospitalización para realizar un procedimiento que exige el protocolo.
- La hospitalización o la prolongación de una hospitalización que forma parte de un procedimiento de rutina del centro
- Hospitalización debida a un trastorno preexistente que no ha empeorado.

Un acontecimiento adverso relacionado con el protocolo es un AA que aparece durante un estudio clínico y que no está relacionado con el producto en investigación, pero que, en opinión del investigador, está relacionado con los requisitos objeto de investigación. Por ej., un AA relacionado con el protocolo puede ser un episodio perjudicial que ocurra durante un periodo de lavado o que esté relacionado con un procedimiento médico exigido por el protocolo.

Deberán registrarse y notificarse los AA y los AAG sufridos por los sujetos desde la firma del consentimiento informado hasta el final de su participación en el estudio.

El investigador o su colaborador interrogará y/o examinará al paciente en busca de indicios de acontecimientos adversos. El interrogatorio de los pacientes en relación con la posible aparición de acontecimientos adversos se hará en forma general (por ej. ¿"Cómo se ha sentido desde la última visita"?). No debe interrogarse al paciente sobre la presencia o ausencia de acontecimientos adversos concretos.

LOS ACONTECIMIENTOS ADVERSOS GRAVES DEBEN SER COMUNICADOS DE FORMA INMEDIATA, EN 24 HORAS AL MONITOR DEL ESTUDIO SEGÚN EL PROCEDIMIENTO DETALLADO EN ESTE PROTOCOLO.

10.3. REGISTRO Y COMUNICACIÓN DE LOS ACONTECIMIENTOS ADVERSOS

10.3.1. ACONTECIMIENTOS ADVERSOS NO GRAVES

Todos los acontecimientos adversos deberán ser registrados en la historia clínica del paciente y en el cuaderno de recogida de datos usando una terminología médica estándar, evitando el uso de expresiones ambiguas o coloquiales. Se anotarán las fechas de comienzo y resolución y la gravedad de todo acontecimiento adverso, así como su relación con el fármaco del estudio.

La gravedad del acontecimiento adverso (leve, moderado, grave) y la relación con el procedimiento (no relacionado, improbable, posible, probable, seguro, o no valorable), se valorará con arreglo a unas directrices que a continuación se especifican. También se anotarán las medidas adoptadas y los resultados obtenidos (por ej. hospitalización, retirada de tratamiento, etc) de todo acontecimiento adverso**.** Los AA que seguramente no estén relacionados con el procedimiento (es decir, comunicados como no relacionados) no serán considerados como reacciones adversas a la terapia celular en los análisis de toxicidad y serán informados de forma independiente.

10.3.2. ACONTECIMIENTOS ADVERSOS GRAVES

Cualquier AAG, tanto si se considera o no en relación con la terapia celular y tanto si se ha administrado o no el producto objeto de investigación, debe ser comunicado inmediatamente al monitor del estudio por teléfono ó fax (en un plazo de 24 horas desde que se tiene noticia de la existencia del acontecimiento. El monitor deberá informar periódicamente, de todos los acontecimientos adversos graves y/o inesperados al Comité de Seguridad. Con esta información el Comité de Seguridad bajo su criterio podrá interrumpir o modificar el tratamiento e incluso producir la salida del estudio del paciente.

La comunicación deberá hacerse a través del formulario de notificación de acontecimientos adversos graves que se incluye en el cuaderno de recogida de datos. Si se realiza por teléfono la notificación, el informe telefónico deberá incluir una descripción detallada del acontecimiento y sus secuelas, además de la relación de causalidad del fármaco en estudio que establezca el investigador.

Deberán notificarse los acontecimientos adversos graves que se produzcan en cualquier momento tras la inclusión del paciente en el estudio, es decir, desde la firma del consentimiento informado por el sujeto y hasta 30 días después de que el paciente haya completado o abandonado el estudio. Se considera que un sujeto ha finalizado el estudio una vez realizada la última visita o el último contacto con el investigador o colaborador. La fecha de retirada o abandono será aquella en la que el paciente y/o el investigador determinen que el sujeto ya no puede cumplir con el protocolo

Se comunicará lo antes posible al CEIC del centro participante en el estudio los informes de los acontecimientos adversos graves, de acuerdo a la legislación actualmente vigente.

Notificación de embarazos: aunque no se consideren acontecimientos adversos graves, si una mujer se queda embarazada en el transcurso de un ensayo clínico, el investigador principal o un colaborador deberá comunicarlo al monitor.

La AEMPS será informada de todos aquellos AAG e Inesperados que guarden relación causal con la terapia celular en estudio, o esta sea posible, probable o desconocida, de la manera prevista en la legislación española sobre ensayos clínicos, empleando el formulario oficial.

Se debe tener en cuenta que los AAG que no han sido documentados previamente en el manual del Investigador, o que ocurren en una forma más grave de lo esperado (es decir, que son “inesperados”), están sujetos a una comunicación rápida a las Autoridades Reguladoras por parte del Promotor. Esto se aplica también a los informes procedentes de fuentes espontáneas y de cualquier tipo de investigación clínica o epidemiológica, independientemente de su diseño o propósito. Siempre debe especificarse la fuente del informe (investigación, espontánea, otra).

LA FECHA Y LA FIRMA DEL INVESTIGADOR RESPONSABLE O DE UNO DE LOS MIEMBROS AUTORIZADOS DE SU PERSONAL DEBEN FIGURAR EN TODOS LOS INFORMES.

10.4. VALORACIÓN DE LA CAUSALIDAD Y SEVERIDAD DE LOS AA

El investigador hará la valoración de causalidad usando las siguientes definiciones:

NO RELACIONADO: No hay evidencia de ninguna relación causal.

IMPROBABLE: Existe poca evidencia que sugiera una relación causal (p.ej. el acontecimiento no se presentó en un periodo de tiempo razonable después de la administración de la terapia celular del estudio). Hay otra explicación razonable para el acontecimiento (p.ej. la condición clínica del paciente, otros tratamientos concomitantes).

POSIBLE: Existen evidencias que sugieren una posible relación causal (p.ej. porque el acontecimiento ocurrió en un tiempo razonable después de la administración de la terapia celular del estudio). Sin embargo, la influencia de otros factores puede haber contribuido al acontecimiento (p.ej. la condición clínica del paciente, otros tratamientos concomitantes).

PROBABLE: Existen evidencias que sugieren una relación causal y la influencia de otros factores es poco probable.

SEGURO: Existen evidencias claras que sugieren una relación causal y se puede descartar una posible contribución de otros factores.

NO VALORABLE: No hay evidencia suficiente o es incompleta para poder hacer un juicio clínico sobre una relación causal

El Investigador, igualmente, valorará la intensidad del acontecimiento atendiendo a la siguiente clasificación:

LEVE: El AA es apreciado por el paciente pero no interfiere con su vida.

MODERADO: El AA produce molestia e interfiere con la vida habitual.

GRAVE: El AA limita severamente la capacidad del paciente de realizar tareas habituales y requiere tratamiento sintomático.

El AA pone en peligro directo la vida del paciente

11. ASPECTOS ÉTICOS

11.1. CONSIDERACIONES GENERALES

Este estudio debe realizarse en conformidad con el protocolo siguiendo los procedimientos normalizados de trabajo del promotor o de la empresa designada.

El ensayo se llevará a cabo de acuerdo a las recomendaciones para Ensayos Clínicos y evaluación de producto en fase de investigación en el hombre, que figuran en la Declaración de Helsinki, revisada en las sucesivas asambleas mundiales (WMA, 2004) (véase Anexo XII), y la actual Legislación Española en materia de Ensayos Clínicos (RD 223/2004). Se seguirán las normas ICH-GCP (CPMP/ICH/135/95).

El producto en investigación se ajusta a la definición de "Medicamento de Terapia Celular" según lo descrito en la Orden SCO/3461/2003 de 26 de Noviembre.

EL CEIC deberá revisar y aprobar el protocolo y el consentimiento informado antes de proceder a la inclusión de sujetos. Antes de realizar cualquiera de los procedimientos especificados en el protocolo, el sujeto participante deberá firmar y fechar el documento de consentimiento informado aprobado por el CEIC.

11.2. INFORMACIÓN QUE SE PROPORCIONARÁ A LOS SUJETOS Y TIPO DE CONSENTIMIENTO QUE SERÁ SOLICITADO EN EL ENSAYO

El consentimiento del sujeto participante en el ensayo se obtendrá siempre por escrito (véase Anexo XIII). El sujeto firmará y fechará el formulario del consentimiento informado antes de ser incluido en el estudio, es decir, antes de emprender cualquier procedimiento del estudio. El investigador explicará la naturaleza del estudio, su propósito, los procedimientos implicados, la duración prevista, los riesgos y los beneficios posibles implicados y cualquier malestar que pueda conllevar, y entregará al sujeto una copia de la hoja de información al paciente y consentimiento firmado. El investigador, informará de que la participación del sujeto en el estudio es voluntaria y que tiene derecho a retirarse del estudio en cualquier momento sin tener que dar explicaciones y sin que su retirada derive en responsabilidad ni perjuicio alguno, afecte a su tratamiento médico posterior o a la relación con el médico que le está tratando. Se dará tiempo suficiente para considerar el estudio antes de decidir si el sujeto va a participar en el ensayo o de retirarse en cualquier momento.

En el caso de que se introduzcan enmiendas al protocolo final que puedan afectar directamente a la participación del sujeto en el estudio (por ejemplo, un cambio en cualquier procedimiento), la hoja de información y el formulario de consentimiento informado deberán cambiarse para incorporar esta modificación y el sujeto deberá firmar el formulario enmendado para indicar que mantiene su consentimiento a participar en el estudio.

El investigador será el encargado de facilitar a cada sujeto o su representante legal una hoja de información al paciente y un formulario de consentimiento informado, que serán los aprobados por el CEIC correspondiente.

El sujeto expresará su consentimiento por escrito. El sujeto deberá cumplimentar él mismo el formulario de consentimiento informado. Una vez firmado y fechado por el sujeto participante (o su representante) y por el investigador, se le entregará una copia. El original de dicho documento se guardará en el centro junto al resto de la documentación del estudio. Ningún paciente podrá participar en el ensayo hasta que haya firmado el consentimiento informado.

11.3. ACCESO A LOS DATOS DEL ESTUDIO

Con el fin de garantizar la confidencialidad de los datos del ensayo, los datos originales serán conservados en el hospital y sólo tendrán acceso a los mismos, el investigador y su equipo de colaboradores, el monitor del ensayo y el Comité Ético de Investigación Clínica del correspondiente centro o el que tutela el ensayo. El investigador permitirá las auditorias y las inspecciones de las Autoridades Sanitarias Españolas o Europeas.

11.4. PROTECCIÓN DE LOS DATOS OBTENIDOS EN EL ESTUDIO

El contenido de los cuadernos de recogida de datos (CRD), así como la confidencialidad de los datos de cada paciente será respetada en todo momento. Se seguirán los procedimientos adecuados para asegurar el cumplimiento de lo recogido en la Ley Orgánica 15/99 de 13 de Diciembre de Protección de Datos de Carácter Personal.

Los documentos generados durante el estudio, serán protegidos del uso no permitido por personas ajenas a la investigación y, por tanto, serán considerados estrictamente confidenciales y no serán revelados a terceros excepto a los especificados en el apartado anterior.

El investigador informará a los sujetos de estudio que los datos obtenidos en el presente ensayo serán guardados y analizados por ordenador y que se seguirán las regulaciones españolas sobre el manejo de datos computerizados.

El investigador acepta que el promotor tenga el derecho a usar los resultados del ensayo clínico incluyendo hojas del CRD o copias de éste. Para permitir el uso de la información obtenida en el ensayo clínico, el investigador comprende que está obligado a suministrar los resultados completos de las pruebas y toda la información desarrollada durante el estudio al promotor.

Se mantendrá en todo momento el anonimato de los sujetos participantes en el ensayo. Los resultados o conclusiones del ensayo clínico se comunicarán prioritariamente en publicaciones científicas antes de ser divulgado al público no sanitario. No se darán a conocer de modo prematuro o sensacionalista, procedimientos de factibilidad aún no determinada.

11.5. SEGURO DEL ENSAYO

La Fundación Progreso y Salud como promotor del estudio, dispone, de acuerdo con la legislación española, de un seguro de responsabilidad civil. Esta póliza cubre todos los posibles daños y perjuicios que el sujeto pueda sufrir a consecuencia de la administración del producto en estudio, de acuerdo a la legislación vigente (RD 223/2004, artículo 8).

Se adjunta la póliza del seguro o indemnización civil suscrita y características de la misma (Anexo XIV).

12. CONSIDERACIONES PRÁCTICAS

12.1. RESPONSABILIDADES DE LOS PARTICIPANTES DEL ENSAYO CLÍNICO.

El promotor, monitor, e investigadores del ensayo cumplirán las responsabilidades establecidas, respectivamente en los Artículos 35, 36 y 37 del RD 223/2004.

Las responsabilidades del investigador principal serán:

1. Estar de acuerdo y firmar junto con el promotor el protocolo del ensayo.
2. Conocer a fondo las propiedades de la terapia celular.
3. Obtener el consentimiento informado de los sujetos antes de su inclusión en el ensayo.
4. Recoger, registrar y notificar los datos de forma correcta y garantizar su veracidad.
5. Notificar inmediatamente los acontecimientos adversos graves o inesperados al promotor.
6. Garantizar que todas las personas implicadas respetarán la confidencialidad de cualquier información acerca de los sujetos del ensayo, así como la protección de sus datos de carácter personal.
7. Informar regularmente al Comité Ético de Investigación Clínica de la marcha del ensayo.
8. Responsabilizarse junto con el promotor de la elaboración del informe final del ensayo, dando su acuerdo al mismo con su firma.

Las responsabilidades del promotor serán:

1. Establecer y mantener un sistema de garantías y control de calidad, con procedimientos normalizados de trabajo escritos, de forma que los ensayos sean realizados y los datos generados, documentados y comunicados de acuerdo con el protocolo, las normas de buena práctica clínica y lo dispuesto en el RD 223/2004.

2. Firmar, junto con el investigador que corresponda, el protocolo y cualquiera de sus modificaciones.

3. Seleccionar al investigador más adecuado según su cualificación y medios disponibles, y asegurarse de que éste llevará a cabo el estudio tal y como está especificado en el protocolo.

4. Proporcionar la información básica y clínica disponible del producto en investigación y actualizarla a lo largo del ensayo.

5. Solicitar el dictamen del CEIC y la autorización de la AEMPS, así como suministrarles la información y recabar las autorizaciones que procedan, sin prejuicio de la comunicación a las comunidades autónomas, en caso de modificación o violación del protocolo o interrupción del ensayo, y las razones para ello.

6. Suministrar de forma gratuita el producto en investigación, terapia celular, y garantizar que se han cumplido las normas de correcta elaboración y que las muestras están adecuadamente envasadas y etiquetadas. También es responsable de la conservación de muestras y sus protocolos de fabricación y control, del registro de las muestras entregadas y de asegurarse de que en el centro donde se realiza el ensayo existirá un procedimiento correcto de manejo, conservación y uso de dichas muestras.

7. Designar el monitor que vigilará la marcha del ensayo.

8. Comunicar a las autoridades sanitarias, a los investigadores y a los CEIC involucrados en el ensayo las sospechas de reacciones adversas graves e inesperadas.

9. Proporcionar al investigador y al CEIC, de forma inmediata, cualquier información de importancia a la que tenga acceso durante el ensayo.

10. Proporcionar compensación económica a los sujetos en caso de lesión o muerte relacionadas con el ensayo. Proporcionar al investigador cobertura legal y económica en estos casos excepto cuando la lesión sea consecuencia de negligencia o mala práctica del investigador.

11. Acordar con el investigador las obligaciones en cuanto al tratamiento de datos, elaboración de informes finales o parciales del ensayo y comunicarlos a quien corresponda.

12. El promotor dispondrá de un punto de contacto, donde los sujetos del ensayo pueden obtener mayor información sobre éste, que podrá delegar en el investigador.

Las responsabilidades del monitor serán:

1. Trabajar de acuerdo con los procedimientos normalizados de trabajo del promotor (en ausencia de los mismos, en este estudio se seguirán los PNTs de la empresa designada para tal efecto), visitar al investigador antes, durante y después del ensayo para comprobar el cumplimiento del protocolo, garantizar que los datos son registrados de forma correcta y completa, así como asegurarse de que se ha obtenido el consentimiento informado de todos los sujetos antes de su inclusión en el ensayo.

2. Cerciorarse de que los investigadores y el centro donde se realizará la investigación son adecuados para este propósito durante el periodo de realización del ensayo.

3. Asegurarse de que tanto el investigador principal como sus colaboradores han sido informados adecuadamente y garantizar en todo momento una comunicación rápida entre investigador y promotor.

4. Verificar que el investigador cumple el protocolo y todas sus modificaciones aprobadas.

5. Comprobar que el almacenamiento, distribución, devolución y documentación del producto en investigación es seguro y adecuado.

6. Remitir al promotor informes de las visitas de monitorización y de todos los contactos relevantes con el investigador.

12.2. MONITORIZACIÓN

Los monitores del ensayo monitorizarán el estudio de forma periódica para asegurar que se salvaguardan los derechos y el bienestar de los pacientes, que se cumple el protocolo, la normativa aplicable, los requisitos éticos, que la documentación necesaria está disponible y que los datos recogidos reflejan exactamente los datos del CRD.

12.3. ENMIENDAS O MODIFICACIONES DEL PROTOCOLO

Una vez aprobado un protocolo por el CEIC y autorizado por la Agencia Española del Medicamento y Productos Sanitarios, ni el investigador ni el promotor pueden hacer modificaciones o alteraciones sin el consentimiento escrito de ambos. Si fuese necesario realizar una modificación o alteración del protocolo, esta modificación deberá ser comentada y acordada entre el investigador principal y el promotor y firmada por ambas partes. Las enmiendas al protocolo formarán parte integral del protocolo original. Cualquier modificación en las condiciones autorizadas para el ensayo que se considere relevante, por afectar a la seguridad de los sujetos, no podrá llevarse a efecto sin el previo dictamen favorable del CEIC y la autorización de la AEMPS.

13. ANÁLISIS ESTADÍSTICO

Los cuadernos de recogida de datos serán procesados por un Sistema Informático de Datos Clínicos prefijado y validado. Tras la doble grabación de datos, resolución de todas las inconsistencias y codificación mediante los diccionarios médicos, se aplicará un proceso de control de calidad final y en caso de cumplirlo la base de datos será considerada libre de errores y se procederá a su congelamiento y a la realización de la explotación estadística de los datos. Los resultados del ensayo clínico se analizarán en la población por intención de tratar.

13.1. POBLACIONES

El análisis de factibilidad se realizará por intención de tratar, se incluirá a todos los pacientes que se les administre terapia celular y se disponga de la evaluación de la RMN (variable principal) tanto en el día 0 (día de administración) como al menos a los 3 meses de seguimiento.

La población de seguridad incluirá a todos los pacientes que se les administre terapia celular.

13.2. TAMAÑO MUESTRAL

Dado que no existen estudios previos para determinar la seguridad ni la factibilidad del tratamiento de la EM con células troncales autólogas obtenidas de tejido adiposo y tratarse por tanto de un estudio exploratorio, se tratará un número de pacientes bajo, de acuerdo con las disponibilidades de producción de células troncales y se evaluarán dos dosis diferentes, para obtener datos orientativos de dosis-efecto que permitan el cálculo de tamaño muestral para un futuro estudio de eficacia si los resultados del presente estudio lo permiten.

13.3. ANÁLISIS ESTADÍSTICOS

13.3.1. DATOS DEMOGRÁFICOS Y BASALES

Se realizará un análisis descriptivo de todas las variables demográficas recogidas así como de los datos clínicos previos al inicio del tratamiento. Las variables cualitativas se expresarán mediante frecuencias absolutas y porcentajes, mientras que las variables cuantitativas se presentarán a través de la media, mediana, desviación típica, máximo, mínimo y número de observaciones.

13.3.2. ANÁLISIS DE LA FACTIBILIDAD

Todos los p-valores e intervalos de confianza serán calculados y evaluados usando un nivel de confianza bilateral del 95%. El principal análisis se realizará con: VARIABLES DEPENDIENTES: Variables clínicas: brotes y disfunción neurológica: EDSS, MSFC. VARIABLES INDEPENDIENTES: Variables analíticas general e inmunológica, LCR, potenciales evocados, TOC, resonancia magnética, calidad de vida. Para evaluar si existe correlación entre los brotes y la progresión clínica de la enfermedad (escalas EDSS y MSFC) se calculará el coeficiente de correlación. Se realizará un análisis exploratorio para comprobar la normalidad de cada una de las variables (PE y progresión clínica de la enfermedad) mediante el test de Shapiro - Wilk. En el caso de que ambas variables se distribuyan según una normal usaremos el coeficiente de correlación de Pearson, en caso contrario haremos uso del coeficiente de correlación de Spearman. Se determinará si los coeficientes de correlación obtenidos son estadísticamente significativos a un nivel de confianza del 95%. Para evaluar la concordancia entre las dos variables cuantitativas

correspondientes a la medición de las alteraciones funcionales detectadas con los PE y las alteraciones observadas con otras posibles técnicas (análisis general e inmunológico, LCR, técnicas de imagen, TCO , pruebas neuropsicológicas y escalas de calidad de vida), se utilizará el coeficiente de correlación intraclase, que se calcula a partir de los estadísticos que produce el análisis de la varianza. Para que la fuerza de la concordancia entre la medida de los mismos atributos sea considerada como buena, es de esperar que el valor del coeficiente se sitúe por encima de 0.71.(Clasificación usada por Fleiss JL. The design and analysis of clinical experiments. New York: Wiley;1986). Un valor inferior, sugeriría que la concordancia entre una y otra medición resultaría inaceptable. También usaremos un procedimiento gráfico alternativo propuesto por Bland JM y Altman DG (Bland J.M., Altman D.G. (1986) Statistical methods for assessing agreement between two methods of clinical measurement. Lancet i: 307-310.), el cual consiste en representar la diferencia entre cada pareja de valores frente a la media de cada pareja de valores. Este método nos aportará unos límites de concordancia a partir del cálculo del intervalo de confianza para la diferencia de dos mediciones, lo que nos ayudará a concluir si las diferencias observadas son o no clínicamente relevantes. Para determinar los factores modificadores de la asociación se aplicarán técnicas de análisis multivariante. La comparación de los resultados entre grupos se hará entre grupo experimental y grupo control y entre grupos según dosis recibida. Los test estadísticos empleados dependerán del tipo de variables, cualitativas o cuantitativas, y de los resultados de los test de normalidad.

13.3.3. ANÁLISIS DE SEGURIDAD

Para el análisis de seguridad, se calcularán el número y porcentaje de pacientes que abandonan el estudio debido a acontecimientos adversos, los pacientes que han experimentado al menos un acontecimiento adverso, los acontecimientos adversos más frecuentes y los pacientes que han experimentado al menos un acontecimiento adverso grave. Se calculará además el intervalo de confianza al 95%.

13.4. DIFICULTADES Y LIMITACIONES DEL ESTUDIO

La característica clínica más llamativa de la Esclerosis Múltiple es su gran variabilidad, estando los síntomas y signos determinados por la localización de las lesiones desmielinizantes que pueden ocurrir a todo lo largo del neuroeje. La principal limitación radica en la dificultad para determinar la presencia/ausencia de brotes y la variabilidad en la medición clínica de la progresión con las escalas EDSS y MSFC. Para minimizar este problema, se establece la existencia de dos neurólogos evaluadores con amplia experiencia en el diagnóstico y seguimiento de pacientes con EM.

Una limitación que puede presentar este proyecto es la escasa duración del mismo, ya que en la EMSP son previsibles pocos brotes y la progresión de la discapacidad es muy lenta medida por las escalas clínicas actuales (EDSS,MSFC), por ello utilizaremos métodos paraclínicos en la evaluación de la factibilidad (RMN, potenciales evocados,…). Otra dificultad estriba en el bajo número de pacientes susceptibles de ser tratados debido a la lentitud de obtención de las células en el laboratorio disponible.

ANEXO I: RESUMEN DEL SEGUIMIENTO

	Visita 1<br>Pre-inclusión	Visita 2<br>+4 sem.	Visita 3<br>+ 1 día	Visita 4<br>+ 8 días	Visita 5<br>+ 30 días	Visita 6<br>+ 60 días	Visita 7<br>+ 90 días	Visita 8<br>+ 6º mes	Visita 9<br>9º mes	Visita 10<br>12º mes
Historia	X	X	X	X	X	X	X	X	X	X
Exploración general	X	X	X	X	X	X	X	X	X	X
Exploración neurológica	X	X	X	X	X	X	X	X	X	X
Brotes	X		X	X	X	X	X	X	X	X
EDSS	X				X	X	X	X	X	X
MSFC	X						X	X	X	X
Analítica general	X	X			X	X	X	X	X	X
Analítica inmunológica	X						X	X		X
Análisis Funcional de respuesta inmune	X					X		X		X
LCR[(1)]	X	X[(1)]								
ECG	X						X			X
RX Tórax	X						X			X
PFR	X						X			X
Criterios inclusión	X	X								
Criterios exclusión	X	X								
Obtención tejido adiposo	X									
Autotrasplante CeTMAd		X								
EA (Escala OMS)			X	X	X	X	X	X	X	X
PE	X							X		X
TOC	X							X		X
RM convencional	X							X		X
TM	X							X		X
ERM	X							X		X
BRB-N	X							X		X
WAIS-III	X							X		X
SF-36	X							X		X
MusiQoL	X							X		X
EQ-5D	X							X		X
Test de embarazo		X								

PFR: Espirometría básica con test de difusión. Criterios de inclusión: Addendum. Criterios de exclusión: Addendum. Obtención tejido adiposo: Técnica desarrolla en el Centro Andaluz de Biología Molecular y Medicina Regenerativa (CABIMER) donde se han desarrollado las células troncales mesenquimales de tejido adiposo CeTMAd. EA: Efectos Adversos según la escala de la Organización Mundial de la Salud. LCR: Líquido Cefalorraquídeo. EDSS: Expanded Disability Statatus Scale. MSFC: Multiple Sclerosis Functional Composite. PE: Potenciales evocados (visuales, auditivos, somatosensitivos, motores, cognitivos). TOC: Tomografía Óptica de Coherencia. RM: Resonancia Magnética. TM: Transferencia de Magnetización. ERM: Espectroscopía por Resonancia Magnética. BRB: Brief Repeatable Battery of Neuropsychologial Test for MS. WAIS-III: Weschler Adult Intelligence Scale.SF-36: MOS-SF-36: MusiQoL: Multiple Sclerosis Quality of Life Scale. EQ-5D: EuroQoL-5.

*El estudio finaliza a los 12 meses de seguimiento pero dado que estos pacientes se ven de forma rutinaria en consulta de los centros participantes en el estudio, se recopilarán datos a los 24 meses para obtener información adicional de seguridad y factibilidad a medio plazo con un protocolo predeterminado.

[(1)]Dependiendo de los resultados obtenidos en la LCR de la visita del mes 1, se podrá repetir dicho procedimiento hasta dos veces más a lo largo del estudio.

ANEXO II: ESCALA DEL ESTADO DE DISCAPICAD EXPANDIDA
SISTEMAS FUNCIONALES DE KURTZKE

0 Exploración neurológica normal (grado 0 en todos los sistemas funcionales [SF]; se acepta función cerebral de grado 1).

1,0 Sin discapacidad, signos mínimos en un SF (es decir, grado 1 en cualquier función que no sea la cerebral).

1,5 Sin discapacidad, signos mínimos en más de un SF (más de un grado 1 en cualquier función que no sea la cerebral).

2,0 Discapacidad mínima en un SF (grado 2 en uno de ellos y 0 ó 1 en el resto).

2,5 Discapacidad mínima en dos SF (grado 2 en 2 SF y 0 ó 1 en el resto).

3,0 Discapacidad moderada en un SF (grado 3 en un SF y 0 ó 1 en el resto), o discapacidad leve en 3 ó 4 SF (grado 2 en ¾ SF y 0 ó 1 en el resto), aunque con plena capacidad de deambulación.

3,5 Plena capacidad de deambulación con discapacidad moderada en un SF (un grado 3) y grado 2 en 1 ó 2 SF, grado 3 en dos SF o grado 2 en 5 SF (0 ó 1 en el resto).

4,0 Plena capacidad de deambulación sin ayuda, autosuficiencia durante un período máximo de 12 horas diarias pese a una discapacidad relativamente grave de grado 4 en un SF (0 ó 1 en el resto) o bien una combinación de grados menores que excedan los límites establecidos en los puntos anteriores. Capacidad de andar unos 500 metros sin ayuda ni descanso.

4,5 Plena capacidad de deambulación sin ayuda durante gran parte del día; capacidad de trabajar la jornada completa, no obstante presentar ciertas limitaciones para realizar una actividad plena o necesitar ayuda mínima; caracterizado por una discapacidad relativamente grave consistente habitualmente en grado 4 en un SF (0 ó 1 en el resto) o una combinación de grados menores que excedan los límites de los puntos anteriores. Capaz de andar unos 300 metros sin ayuda ni descanso.

5,0 Capacidad de andar unos 200 metros sin ayuda ni descanso. Discapacidad lo suficientemente grave como para afectar a la actividad diaria habitual. Equivalente a un 5 en un sólo SF y 0 ó 1 en el resto, o una combinación que supere las especificaciones del punto 4.0.

5,5 Capacidad de andar unos 100 metros sin ayuda ni descanso; discapacidad lo suficientemente grave como para impedir la actividad diaria habitual. (Equivalente a un grado 5 en un solo SF y 0 ó 1 en el resto o una combinación de grados menores que suelen superar a los del punto 4.0).

6,0 Necesidad de ayuda intermitente o constante unilateral (bastón, muleta o corsé) para andar unos 100 metros con o sin descanso. (Equivalente a combinaciones de más de dos grados 3+ en los SF).

6,5 Necesidad de ayuda bilateral constante (bastones, muletas o corsé) para andar unos 20 metros sin descansar. (Equivalente a combinaciones de más de dos grados 3+ en los SF).

7,0 Incapaz de andar más de 5 metros incluso con ayuda y limitado esencialmente a permanecer en silla de ruedas; capaz de desplazarse solo en la silla de ruedas y de levantarse de ella; permanece en la silla de ruedas unas 12 horas diarias. (Equivale a combinaciones de más de un grado 4+ en los SF y, muy raramente, a un grado 5 de la función piramidal únicamente).

7,5 Incapaz de dar unos cuantos pasos; limitado a permanecer en silla de ruedas; puede necesitar ayuda para levantarse de la silla; capaz de desplazarse solo en la silla, aunque no todo el día; puede necesitar una silla de ruedas con motor. (Equivale a combinaciones de más de un grado 4+ de los SF).

8,0 Limitado esencialmente a estar en cama o sentado o a ser trasladado en silla de ruedas, aunque puede permanecer fuera de la cama gran parte del día; capaz de realizar gran parte del aseo personal; puede utilizar las manos eficazmente. (Equivale a combinaciones de grado 4+ en varios sistemas).

8,5 Limitado a permanecer en cama gran parte del día; puede utilizar parcialmente las manos y realizar algunas labores de aseo personal. (Equivale a combinaciones de grado 4+ en varios sistemas).

9,0 Paciente encamado, inválido; puede comunicarse y comer. (Equivale a combinaciones de grado 4+ en la mayoría de los sistemas).

9,5 Paciente encamado y totalmente inválido, incapaz de comunicarse eficazmente y de comer o deglutir. (Equivale a combinaciones de grado 4+ en casi todos los sistemas).

10. Muerte por EM.

ANEXO III: PROTOCOLO DE RESONANCIA MAGNÉTICA

Introducción

El estudio de RM constará de cuatro exploraciones seriadas, con un examen basal y seguimientos a los, seis, y doce meses. Se propone un protocolo de RM que permita cuantificar los parámetros de interés. Concretamente, se pretende cuantificar el volumen total de las lesiones detectadas en una secuencia de densidad de protones/T2, en la secuencia FLAIR y el volumen total de lesiones captantes de Gadolinio en una secuencia spín-eco T1. La secuencia T1 sin contraste evaluará los llamados “agujeros negros” crónicos, lesiones que representan daño tisular irreversible y son consideradas un marcador de progresión de discapacidad. Además se cuantificará el volumen cerebral total y el volumen de sustancia blanca, sustancia gris y LCR utilizando la adquisición volumétrica eco de gradiente para objetivar la pérdida de parénquima cerebral en la progresión de la enfermedad.

Nuestro protocolo también incluye secuencias de RM no convencionales como las de difusión, trasferencia de magnetización y espectroscopia para valorar otros aspectos de la enfermedad como la cuantificación de la extensión del daño en la sustancia blanca aparentemente normal en las secuencias convencionales y el daño de la sustancia gris.

El análisis de las imágenes se realizará de forma centralizada en la Unidad de Resonancia Magnética de cada hospital participante. Todas las imágenes serán grabadas en CD con formato DICOM.

Todas las exploraciones de RM deben realizarse utilizando las mismas secuencias y con el mismo reposicionamiento. Es necesario que las secuencias del estudio estén grabadas en el directorio de protocolos de los equipos de RM.

Protocolo de adquisición

Secuencias de localización

a. Localizador axial.
b. Localizador (oblicuo) coronal, perpendicular a la cisura interhemisférica.
c. Localizador (oblicuo) sagital en la orientación de la cisura interhemisférica.

Con este último localizador, se prescriben las secuencias D/T2 y T1, orientadas al margen inferior de la rodilla y esplenio del cuerpo calloso.

Los localizadores corresponden a secuencias potenciadas en T1 con calidad suficiente para poder identificar fácilmente las marcas anatómicas que se precisen. Parámetros:

- Secuencia potenciada en T1 (spin-eco o fast/turbo spín-eco) ; TR: 200-500; TE: 10-20; FOV: 250; 3-5 mm de grosor de corte; Número de cortes: 1-3; Matriz: 128 x 256; 1 adquisición

Secuencia transversal en densidad de protones/T2 (DP/T2)

Secuencia transversal (oblicuo-axial) potenciada en DP y T2 (fast/turbo spín-eco, opcionalmente spin-echo convencional). El objetivo es utilizar cortes de 3 mm. de grosor, intercalados, que cubran todo el parénquima cerebral desde el agujero magno hasta el vértex. Las líneas de posición de esta secuencia sobre el localizador sagital quedarán grabadas en la máquina. Parámetros:

44 - 46 cortes
3 mm. grosor intercalados ("interleaved"). Supone dos adquisiciones.
Orientación transversal siguiendo el margen inferior de la rodilla y esplenio del cuerpo calloso.

- FOV: 250 mm.; Matriz: 256x256.; Codificación de fase: izquierda → derecha; Número de excitaciones: 1; Banda de saturación inferior; TR= entre 3000 y 4000 mseg.; TE= 15-20; 80-90; Eco tren: 6 - 8

Secuencia 3D

Se pretende adquirir una secuencia volumétrica con buena relación señal /ruido que permita obtener medidas fiables del volumen del parénquima cerebral. Para conseguir este objetivo y uniformar al máximo la adquisición los parámetros de esta secuencia deberán ser:

- 3D sagital (en SPGR o FLASH) prescrita sobre una imagen axial.; FOV 250 mm; Angulo (flip angle) 30 grados; TR 40 mseg; TE mínimo; Matriz 256 x 192; Frecuencia S/I (fase A/P); 1nex (no emplear adquisición fraccionada); Mínimo número de cortes: 60; Grosor de corte entre 2,3 mm y 2,7 mm, en función del tamaño del cerebro. Es importante incluir todo el parénquima cerebral.

Secuencia transversal FLAIR

Secuencia transversal (oblicuo-axial) FLAIR (Fluid-Attenuated Inversion Recovery). El objetivo es utilizar cortes de 5 mm. de grosor, intercalados, que cubran todo el parénquima cerebral desde el agujero magno hasta el vértex. Parámetros:

- Flair, 2D; 30 cortes; 5 mm. grosor intercalados ("interleaved"). Supone 3 adquisiciones.

Orientación transversal siguiendo el margen inferior de la rodilla y esplenio del cuerpo calloso.

- FOV: 250 mm; Matriz: 256x192;Codificación de fase: izquierda → derecha; Número de excitaciones: 2 (tiempo adquisición aprox. 6:34); Receive bandwidth= 16 KHz; TR=8200; TE= 185; TI= 2000

Secuencia spín-eco transversal potenciada en T1

El objetivo es utilizar cortes de 5 mm. de grosor contiguos que cubran todo el parénquima cerebral.

- 30 cortes;5 mm. Grosor. Cortes intercalados ("interleaved").

Orientación transversal siguiendo el margen inferior de la rodilla y esplenio del cuerpo calloso.

- FOV: 250 mm; Matriz: 256x256; Codificación de fases: izquierda →derecha; Número de excitaciones: 1; Banda de saturación inferior (sí lo permite el TR); TR= 600-650; TE= 10-15.

Secuencia spín-eco transversal potenciada en T1 con Gadolinio

Secuencia idéntica a la anterior tras la administración de Gadolinio. Inyectar el contraste **SIN** modificar la posición del paciente. **Dosis de contraste: 0.2cc/Kg de peso.** Tiempo de espera tras la inyección de contraste: 5-10 minutos.

Secuencia difusión isotrópica

El objetivo es utilizar cortes contiguos que cubran todo el parénquima cerebral. En todos los casos se calculará el coeficiente de difusión aparente (ADC)

20-25 cortes,, 5mm de grosor

Orientación transversal siguiendo el margen inferior de la rodilla y esplenio del cuerpo calloso.

- FOV: 250 mm; Matriz: 94x94; Codificación de fases: izquierda →derecha; Coeficiente de difusión b 1000

Trasferencia de magnetización

El objetivo es utilizar cortes de 5 mm. de grosor, con un número máximo de 20 cortes. Se realizará una adquisición sin y otra con pulso de transferencia de magnetización. Los ratios de ambas adquisiciones se usarán para cuantificar la integridad de la sustancia blanca mielinizada en diferentes zonas cerebrales.

Orientación transversal siguiendo el margen inferior de la rodilla y esplenio del cuerpo calloso.

- FOV: 250 mm; Matriz: 256x256; Codificación de fases: izquierda →derecha; Número de excitaciones: 1; Banda de saturación inferior (sí lo permite el TR); TR= 600 TE= 12.

Espectroscopía de protón de Hidrógeno

El espectro por RM ofrece valiosos datos bioquímicos del volumen de tejido cerebral estudiado. Se realizará espectro de vóxel único de 2cm en las lesiones que muestren cambios inflamatorios agudos y en la sustancia blanca aparentemente normal. Se pretende estudiar el comportamiento de los metabolitos cerebrales, especialmente del NAA, Creatina y Colina como marcadores de daño axonal, cambios inflamatorios y procesos de desmielinización y remielinización.

ANEXO IV: PROTOCOLO NEUROFISIOLÓGICO

Objetivos:
Seguimiento del tratamiento.

Estudio analítico observacional: estudio longitudinal de seguimiento:

Los pacientes con EM incluidos en el estudio serán evaluados de forma longitudinal para todos los potenciales evocados, al inicio del estudio antes del comienzo del tratamiento y después a los 6 meses al primer y segundo año post-tratamiento.

Variables neurofisiológicas (potenciales evocados)

- Potenciales evocados visuales: latencia P100: variable cuantitativa (ms); amplitud N75-P100: variable cuantitativa (μV)
- Potenciales evocados acústicos: intervalo I-V: variable cuantitativa (ms); amplitud I/V variable cuantitativa (μV))
- Potenciales evocados somatosensitivos n. mediano: latencia N20: variable cuantitativa (ms); amplitud p14-N20 variable cuantitativa (μV); tiempo de conducción central N13-N20 : variable cuantitativa (ms)
- Potenciales evocados somatosensitivos n.tibial: latencia P39: variable cuantitativa (ms); amplitud P30-P39 variable cuantitativa (μV); tiempo de conducción central N22-P39: variable cuantitativa (ms)
- Potenciales evocados motores de miembros superiores e inferiores: tiempo de conducción central al músculo abductor del primer dedo y al tibial anterior: variable cuantitativa (ms)
- Potencial evocado cognitivo P300: latencia: variable cuantitativa (ms) y amplitud: variable cuantitativa (μV)
- Escala global paramétricas de puntuación generadas de la combinación de varios potenciales evocados: EPAS (del inglés, "Evoked Potentials Abnormality Score): para cada modalidad se generará una variable cuantitativa ordinal (0=normal, 1=latencia incrementada, 2=latencia incrementada con cambios morfológicos/disminución de amplitud, 3=ausencia de algún componente principal del potencial evocado) generada con la suma de ambos lados para los PEV y los PEAT (0-6), y para cada lado para los PES y PEM (0-12). La escala general combinada va de 0 a 36, donde los valores más elevados representan mayor afectación de los potenciales evocados.(Leocani L et al, 2006)

Métodos

Potenciales evocados

- **Potenciales evocados visuales (PEV)**
- **Potenciales evocados acústicos de tronco (PEAT)**
- **Potenciales evocados somatosensitivos del nervio mediano (PES N. Mediano)**
- **Potenciales evocados somatosensitivos del nervio tibial (PES N. tibial)**
- **Potenciales evocados motores (PEM) de miembros superiores**
- **PEM de miembros inferiores**

Los potenciales evocados se obtendrán de acuerdo a las guías publicadas *(Deuschl G, Eisen A. Recommendations for the practice of clinical neurophysiology: Guidelines of the International Federation of Clinical Neurophysiology. Electroencephalogr Clin Neurophysiol 1999; 52(Suppl):192-211)* y se compararán con los valores de normalidad obtenidos en nuestro laboratorio.

ESTUDIOS ESTADÍSTICOS

Los datos clínicos y de HLA serán analizados con un programa estadístico

Se utilizarán test univariantes paramétricos y no paramétricos o multivariantes (regresión logística o lineal) en función de las variables en comparación. Para cada comparación se considerarán diferencias estadísticamente significativas cuando se obtengan unos valores de p corregida < 0.05 y se usará el método de Bonferroni para la corrección de comparaciones múltiples cuando sea preciso.

ANEXO V: PROTOCOLO NEUROPSICOLÓGICO

Evaluación Neuropsicológica en Esclerosis Múltiple

Introducción.
Sobre el 50 % de las personas con EM desarrollan algún tipo de disfunción cognitiva, sólo entre el 5 y 10 % desarrollan déficit severos, que hacen muy difícil la vida diaria. Estos déficit pueden aparecer al principio de la enfermedad o tras mucho tiempo de evolución.

La enfermedad puede producir déficit cognitivos por dos vías:

Vía directa: La extensión de la dismielinización se relaciona con la severidad del daño cognitivo.

Vía Indirecta: La fatiga, la ansiedad o la depresión que suele acompañar a la enfermedad pueden provocar estos déficit cognitivos.

Los principales déficits cognitivos encontrados en la EM son:

- Velocidad de procesamiento de información.
- Funciones ejecutivas (planificación y priorización).
- Razonamiento abstracto.
- Resolución de problemas.
- Problemas en atención/concentración.
- Problemas de evocación.
- Problemas de memoria.

Alteraciones Neuropsicológica en Esclerosis Múltiple

- CAPACIDAD INTELECTUAL

Los pacientes con EM suelen presentar alteraciones en pruebas estandarizadas que valoran la capacidad intelectual (CI), con en la escala de inteligencia de Wechsler para adultos WAIS.

En general las diferencias con respecto a los grupos controles se ponen de manifiesto tanto en el CI verbal como manipulativo. En algunos estudios longitudinales se ha descrito un ligero declive en el coeficiente intelectual, con una mayor conservación del CI verbal a lo largo del tiempo.

- RAZONAMIENTO ABSTRACTO

Por lo que respecta a las tareas de resolución de problemas y razonamiento abstracto/conceptual (test de clasificación de cartas de Wisconsin, test de matrices progresivas de Raven) la mayoría de los estudios observan rendimientos inferiores (menor número de categorías y más errores de perseveración) en el grupo de pacientes.

- LENGUAJE

La presencia de alteraciones del lenguaje no es frecuente, excepto cuando se trabaja con pacientes con una mayor afectación, en los que aparecen descritos trastornos en denominación y comprensión en pruebas como el Test de Denominación de Boston.

- MEMORIA

El recuerdo está más afectado que el reconocimiento. Con respecto a la memoria no todos los tipos de ésta se afectan por igual, así se afectan sobre todo la memoria a la largo plazo y la memoria de trabajo, por lo que en muchas ocasiones lo que se observa es una dificultad para el "rescate" de la información, más que un problema de almacenamiento.

Escalas para la valoración neuropsicológica.

The Brief Repeatable Battery of Neuropsicological Test for MS BRB-N

Esta prueba es sensible al daño cognitivo temprano y se puede administrar en media hora.

La batería consta de 5 pruebas:

- Buschke Selective Reminder Test (mide memoria selectiva)
- 10/36 Spatial Recall Test (memoria espacial-visual)
- Paced Auditory Serial Addition Task (PASAT) (mide velocidad procesamiento de información)
- Symbol Digit Modalities Test (velocidad procesamiento de información)
- Controlled Oral Word Association Test (mide lenguaje, memoria semántica)

Su especificidad en EM es del 94% y su sensibilidad del 71% (Rao, 2004), aunque otros autores sugieren que no sirve para monitorizar la respuesta terapéutica y que no detecta bien los déficits de memoria.

El problema del uso de las pruebas de evaluación breve es que es frecuente la obtención de porcentajes altos de falsos negativos (pacientes que aún presentado déficit cognitivos son calificados como normales).

Escala de Inteligencia de Wechsler para Adultos. WAIS-III

La idea de utilizar el WAIS es a su vez, la idea de utilizar un concepto, el del medir la capacidad intelectual frente a las funciones cognitivas básicas para las que se emplea la BRB-N. Con esta prueba obtendremos el CI total como medición de la capacidad intelectual, al tiempo de poder ver las diferencias entre el CI verbal y el CI manipulativo.

Además el WAIS-III permite realizar otras agrupaciones basadas en unos aspectos más precisos de funcionamiento cognitivo, que dan lugar a índices de Comprensión verbal, Organización perceptiva, Memoria de trabajo y Velocidad de procesamiento.

ANEXO VI: PROTOCOLO DE CALIDAD DE VIDA

CUESTIONARIO SF-36

Se adjunta

CUESTIONARIO EQ-5D

Se adjunta

CUESTIONARIO MusiQoL

Se adjunta

ANEXO VII: TABLA DE LA OMS PARA GRADACIÓN DE LA TOXICIDAD

	Grade: 0	Grade: 1	Grade: 2	Grade: 3	Grade: 4
Hematologic (Adults)					
Hemoglobin	>= 6.8 mmol/l<br>>110 g/l	5.6 - 6.7 mmol/l<br>95 - 109 g/l	4.95 - 5.8 mmol/l<br>80 - 94 g/l	4.0 - 4.9 mmol/l<br>65 - 79 g/l	< 4.0 mmol/l<br><65 g/l
Leukocytes (10^9/l)	>= 4.0	3.0 - 3.9	2.0 - 1.9	1.0 - 1.9	< 1.0
Granulocytes (10^9/l)	>= 2.0	1.5 - 1.9	1.0 - 1.4	0.5 - 0.9	< 0.5
Platelets (10^9/l)	> 100	75 – 99	50 - 74	25 - 49	< 25
Hemorrhage	None	Mild, petechiae, no transfusion	Mild blood loss, 1-2 units transfusion per episode	Gross blood loss, 3-4 units transfusion per episode	Debilitating massive blood loss, > 4 units transfusion per episode
Hepatic					
Bilirubin	=<1.25 x N(a)	1.26 - 2.5 x N(a)	2.6 - 5 x N(a)	5.1 - 10 x N(a)	> 10 x N(a)
Transaminases (ASAT/ALAT)	=<1.25 x N(a)	1.26 - 2.5 x N(a)	2.6 - 5 x N(a)	5.1 - 10 x N(a)	> 10 x N(a)
Alkaline phosphatase	=<1.25 x N(a)	1.26 - 2.5 x N(a)	2.6 - 5 x N(a)	5.1 - 10 x N(a)	> 10 x N(a)
Clinical	Not included	Not included	Not included	Precoma	Hepatic coma
Gastrointestinal					
Oral (mucositis; stomatitis)	No change	Soreness/erythema	Erythema, ulcers, can eat solids	Ulcers, requires liquid diet only	Ulcers with hemorrhage & necrosis, alimentation not possible
Esophagus	None	Mild fibrosis, no pain, slight difficulty with solid food	Fibrosis, mild pain, semi-solid food only	Severe fibrosis, severe pain, liquid only, dilatation req'd	Necrosis, fistula, perforation
Anorexia/weight loss	None	Anorexia without weight loss	Weight loss <5%	Weight loss 5-10%	Weight loss >10%
Nausea/vomiting	None	Nausea without vomiting	Transient vomiting; 1-5 emetic episodes/day	Vomiting requiring therapy; 6-10 emetic episodes/day	Intractable vomiting
Diarrhea	None	Transient (< 2	Tolerable, but >	Intolerable,	Hemorrhagic;

	Grade: 0	Grade: 1	Grade: 2	Grade: 3	Grade: 4
		days); 3-4 loose stools/day	2 days; 5-7 loose stools/day	requiring therapy; > 7 loose stools/day	dehydration & electrolyte imbalance
Renal, bladder					
Blood urea (urea-nitrogen) or creatinine	=<1.25 x N(a)	1.26 - 2.5 x N(a)	2.6 - 5 x N(a)	5.1 - 10 x N(a)	> 10 x N(a)
Creatinine clearance	Normal range	0.75-0.99L(a)	0.5-0.74L(a)	0.25-0.49L(a)	<0.25L(a)
Proteinuria	No change	1 +, < 3 g/l	2 - 3+, 3 - 10 g/l	4+, > 10 g/l	Nephrotic syndrome
Hematuria	No change	Microscopic	Gross	Gross + clots	Obstructive uropathy
Pulmonary					
Respiratory symptoms	None	Mild or transient dyspnea	Exertional dyspnea	Dyspnea at rest, req's intermittent oxygen	Complete bed rest, continuous oxygen or assisted ventilation req'd
X-ray	Normal	Linear streaking	Bilateral, opacification < 50% lung volume	Opacification 50 - 75%	Opacification > 75%
Function	Normal	2550% decrease in DCD or VC	> 50% decrease in DCD or VC	Not included	Not included
Allergic	None	Transient rash, edema	Urticaria, bronchospasm; no parenteral therapy needed	Serum sickness, bronchospasm; parenteral therapy required	Anaphylaxis
Cutaneous	No change	Erythema, macules, papules, pigmentation, slight atrophy, nail changes	Dry desquamation, vesiculation, pruritus	Moist desquamation, ulceration	Exfoliative dermatitis, necrosis requiring surgical intervention
Hair	No loss	Minimal hair loss	Moderate, patchy alopecia	Complete alopecia, but reversible	Complete & non-reversible alopecia
Infection, specify site	None	Minor infection, no active Rx	Moderate infection, req's active Rx	Major infection, hospitalization	Major, life threatening, infection with

	Grade: 0	***Grade: 1***	***Grade: 2***	***Grade: 3***	***Grade: 4***
					hypotension
Cardiac					
Rhythm	Normal	ST-T changes, sinus tachycardia > 110 at rest	Unifocal PVC, atrial arrhythmia	Multifocal PVC	Ventricular tachycardia, intractable CHF
Function	No change	Asymptomatic, but abnormal cardiac sign	Transient asymptomatic dysfunction; no therapy required	Symptomatic dysfunction responsive to therapy	Symptomatic dysfunction non-responsive to therapy
Left Ventricular Ejection Fraction (LVEF)	No drop in LEV, as compared with initial value	LVEF drops 1-5%	LVEF drops 6-10%	LVEF drops 11-15%	LVEF drops > 15%
Ischemia	Not scored	Not scored	Not scored	Transient angina or ischemic changes on EKG	Myocardial infarction
Pericarditis	No change	Asymptomatic effusion and/or ECG suggestive of pericarditis	Symptomatic, no tap required	Effusion req'g taps or constrictive pericarditis not req'g surgery	Tamponade or constrictive pericarditis req'g pericardectomy
Neurologic					
State of consciousness, cortical	Alert	Transient lethargy or agitation	Somnolence < 50% of waking hours or moderate agitation	Severe somnolence > 50% of waking hours; agitation or confusion; disorientation, hallucination	Coma; seizures or toxic psychosis
Mood	No change	Mild, only mentioned after questioning	Moderate, easily in tears	Severe, (anxiety, depression) visibly depressed/anxious, pharmacotherapy indicated	Suicidal ideation, clinical treatment indicated
Cerebellar	None	Slight incoordination	Intention tremor, dysmetria, slurred speech, nystagmus	Locomotor ataxia	Cerebellar necrosis
Peripheral, sensory	Normal	Mild paresthesias and/or decreased tendon reflexes	Moderate paresthesias, absent DTR's	Severe paresthesias and/or disabling objective sensory	Intolerable paresthesias and/or function

	**Grade: 0**	**Grade: 1**	**Grade: 2**	**Grade: 3**	**Grade: 4**
				loss interfering with function	loss
Peripheral, motor	Normal	Subjective weakness	Mild objective weakness without significant impairment of function	Objective weakness with impairment of function	Paralysis
Constipation (b)	None	Mild	Moderate	Abdominal distension	Constipation, distension and vomiting req'g surgery
Bladder function	Normal	Mild dysfunction	Moderate dysfunction	Severe bladder dysfunction	Dysfunction & distension req'g surgery
Pain (c)	None	Mild	Moderate	Severe	Intractable
Other toxicities					
	Grade : 0	***Grade: 1***	***Grade: 2***	***Grade: 3***	***Grade: 4***
Fever with drug	None	Fever < 38 C	Fever 38 C - 40 C	Fever > 40 C, or with chills	Fever with hypotension
Fatigue	None	Mild; fatigue without decrease in daily activities	Moderate, periodic interfering with performance; in bed < 50% of the day	Severe, clearly interfering with performance status; in bed > 50% of the day	State of exhaustion with very poor performance status; unable to take care of self
Flu-like syndrome	None	Mild	Moderate	Severe	Intractable
Headache	None	Mild	Moderate	Severe	Intractable
Flushing	None	Mild	Moderate	Severe	Intractable
Myalgia/arthralgia	None	Mild	Moderate	Severe	Disabling
Local reaction (injection site)	None	Pain	Pain + inflammation	Ulceration	Surgery indicated
Vasculitis	None	Restricted cutaneous	Generalized cutaneous	Hemorrhagic	Systemic
Other	None	Mild	Moderate	Severe	Life-threatening

N(a) = Upper limit of normal value of population under study. L(a) = lower normal value of the local institution b = This does not include constipation resultant from narcotics. c = Only treatment related pain is considered, not disease related pain (the usage of narcotics may be helpful in grading pain, depending upon the tolerance level of the patient)

ANEXO VIII: IDENTIFICACIÓN DEL PRODUCTO EN INVESTIGACIÓN

ETIQUETADO

1.- Las jeringas de suspensión celular/ placebo llevarán en su etiqueta los siguientes datos:

- "Suspensión celular conteniendo: ....
- "Promotor del ensayo: ....
- Código de referencia del ensayo: ....
- Número de lote: ....
- Código del paciente: ....
- Fecha y hora de caducidad: ....

2.- Etiqueta del embalaje refrigerado:

- Suspensión celular conteniendo: ....
- "Exclusivamente para ensayos clínicos"
- "Exclusivamente para uso autólogo"
- "Promotor del ensayo: ....
- Código de referencia del ensayo: ....
- Número de lote: ....
- Código del paciente: ....
- Conservar refrigerado de 2 – 8ºC: ....

Irá dirigida: "A la atención del Dr. .... (nombre del investigador responsable de la implantación de las MSC).

Lista de contenido del producto

Cada lote de producto llevará un formulario impreso, llamado lista de contenido del producto, que deberá rellenar la persona que prepara dicho lote. El contenido de este formulario es el siguiente:

Este producto es exclusivamente para uso en ensayos clínicos.

Este producto es exclusivamente para uso autólogo.

- Promotor del ensayo: ....
- Código de referencia del ensayo: ....
- Número de lote: ....
- Código del paciente: ....
- Número de viales: ....
- Número de células por jeringa: ....
- Viabilidad celular en el momento del envasado: ....
- Fecha y hora de caducidad: ....

Manual de uso del producto

El "Manual de uso del producto" es un documento impreso que acompaña a cada lote de producto en el que se describen las características del mismo, su identificación, y su método de utilización (implantación en el paciente). El contenido de este documento es el siguiente:

- Suspensión celular conteniendo: ....
- Este producto es "Exclusivamente para ensayos clínicos"
- Este producto es "Exclusivamente para uso autólogo"

- El producto es una suspensión celular en solución tamponada estéril/placebo conteniendo ……………………….. de origen autólogo. Dicha solución se presenta en viales de un solo uso y está indicada para la investigación local tras su suspensión. Cada vial contiene aproximadamente ……………

ANEXO IX: PERFIL DE CITOCINAS y ANALISIS FUNCIONAL DE LA RESPUESTA INMUNE

Dentro de Objetivos:

Valorar el efecto inmunomodulador de la terapia celular sobre la activación de células mononucleares periféricas de los pacientes con EM mediante el estudio de sus perfiles de citoquinas: Porcentajes de células T, NK y monocitos productores de citoquinas proinflamatorias (TNF-α, IFN-γ, IL-2, IL-12) y antiinflamatorias (IL-4, IL-5, IL-10 y TGF-beta), así como de citoquinas críticas para el desarrollo de enfermedades autoinmunes (IL-17A, IL-6 e IL-23) en los pacientes con EM, antes de comenzar la terapia celular y a los 3, 6 y 12 meses de la infusión celular.

Material y métodos :
Variables de laboratorio

Determinación de niveles de citoquinas intracelulares en células mononucleares periféricas tras activación policlonal: variable cuantitativa continua expresada como porcentaje de una determinada subpoblación celular (linfocitos T CD4+, Linfocitos T CD8+, Linfocitos NK y monocitos)

Toma de muestras.-

A cada paciente se le extraerán 10 ml de sangre venosa periférica basalmente (inmediatamente antes de ser infundidos) y en los meses 3, 6, y 12, de tratamiento para el análisis del perfil de citocinas y 10 ml de sangre venosa periférica que se deteminará en la visita antes de la infusión de células mesenquimales, y en los meses 2, 6 y 12..

a) Determinación del perfil de citoquinas por citometría de flujo,

Aislamiento de células mononucleares de sangre periferica.

Las células mononucleares de sangre periférica (PBMC) serán aisladas a partir de sangre venosa heparinizada por centrifugación en gradiente de densidad sobre Ficoll. Las PBMC se cultivarán a una concentración de 2 x 10^6 células/ml en RPMI 1640 suplementado con 10% suero bovino fetal y L-glutamina 2mM en presencia de activadores policlonales de la expresión de citoquinas: Forbol Miristato Acetato (PMA) 25 ng/ml e Ionomicina (I) 1 μg/ml durante 4-6 horas a 37ºC en atmósfera con 5% CO2. Durante las dos últimas horas se añade un inhibidor del transporte proteico, Brefeldina A (Sigma) a 10 μg/ml. Para evaluar el grado de síntesis espontáneo de citoquinas procedentes de la activación in vivo se cultivarán las células a la misma concentración pero sin activarlas policlonalmente, sometiéndolas exclusivamente a la acción de la Brefeldina A. Como control de activación se utilizará la expresión de CD69 PE en superficie celular.

Tras la incubación, las células se lavarán, y se procederá al marcaje exterior mediante marcadores fenotípicos de membrana de las diferentes subpoblaciones celulares (CD3, CD4, CD8, CD14, CD56), conjugados a PerCP, o APC. Posteriormente se permeabilizarán las células para el marcaje interno con anticuerpos frente a las diferentes citoquinas intracelulares que queremos determinar: IFN-γ, TNF-α, IL-2, IL-12, IL-4, IL-5, IL-10, IL-17A, IL-23 e IL-6 conjugados a FITC o PE.

Cada tinción se acompañará de una tinción control con anticuerpos monoclonales de especificidad irrelevante del mismo isotipo.

Terminada la incubación, se lavarán las células y se analizarán en un citómetro de flujo FACScalibur usando el programa Cell Quest. Se adquieren 15.000 eventos por tinción y se realiza un análisis de 6 parámetros: la dispersión frontal y lateral de la luz (FSC, SSC) de cada grupo celular y las 4 fluorescencias con FITC, PE, PerCP y APC, dependiendo de cada tinción. Los resultados se expresaránn como porcentajes de expresión de cada citoquina en una determinada subpoblación celular y como medias de intensidad de fluorescencia para cada citoquina en cada subpoblación celular.

b) Análisis funcional de la respuesta inmune

Tiempos de recolección (a estudiar por el equipo clínico la idoneidad)

1. Antes de la infusión de células mesenquimales autólogas de tejido adiposo.
2. 2 meses desde la infusión de células mesenquimales autólogas de tejido adiposo.
3. 6 meses desde la infusión de células mesenquimales autólogas de tejido adiposo.
4. 12 meses desde la infusión de células mesenquimales autólogas de tejido adiposo.

Preparación de las muestras

1. Preparar suero
2. Separación por Ficoll de células.. **Tomar células para A y B.**
3. Selección positiva para células dendríticas (DCs) (MACs, Miltenyi). **Tomar células para C.**
4. Selección positive para células B (MACs, Miltenyi). **Tomar células para D.**
5. Selección negative para células CD4+ T (MACs, Miltenyi). **Tomar células para E.**
6. FACS sorting de células $CD4^{+}CD25^{+}CD62L^{high}$ Treg y $CD4^{+}CD25^{-}CD62L^{high}$ Teff.**Tomar células para F.**

Experimentos a realizar

A. Backup de las muestras. Congelación de células para futuros análisis (gene expression, wide-genome scan, etc..).

B. Proliferación de PBMCs en respuesta a la estimulación con anti-CD3 y copaxone (el copaxone se incluye como un surrogate antigen por su reactividad cruzada con antígenos de mielina). Recoger sobrenadantes para análisis mediante CBA (Cytometric Bead Array, CBA, Pharmingen).

C. DCs. Activar DCs con un panel de ligandos para TLR (Toll-like receptor, incluimos ligandos endógenos como HSP70 y HSP60), y análisis de la expresion de citocinas/quimoquinas por CBA y qRT-PCR. Realización de experimentos con FACS para el estudio de la expresión en superficie de MHC-I y MHC-II, así como de moléculas co-estimulatorias (CD80, PD1, etc).

D. Células B. Activación de células B con anti-BCR o ligandos de TLR y seguimiento de la respuesta proliferativa y la secreción de citocinas, mediante incorporación de timidina, CBA y FACS, respectivamente.

E. Proliferación de células T . Activación de células T CD4 con anti-CD3/CD28 y copaxone y seguimiento de la respuesta proliferativa y secreción de citocinas mediante mediante incorporación de timidina, CBA y FACS, respectivamente.

F. Actividad de las células Treg. Coincubación de células $CD4^{+}CD25^{+}CD62L^{high}$ (T. reg) y $CD4^{+}CD25^{-}$ $CD62L^{low}$ (T. Eff) tras sorting para ensayar la actividad supresora en respuesta a la activación con anti-CD3/CD28. Determinación de la proliferación y la liberacin de citocinas mediante CBA.

c) Perfiles de expresión génica, metabolómica y análisis con arrays frente a antígenos

ANEXO X: PROTOCOLO DE PROCESAMIENTO Y ANÁLISIS DE LÍQUIDO CEFALORRAQUÍDEO

Protocolo para el procesamiento del líquido cefalorraquídeo

Como parte del estudio previo para la inclusión del paciente y de su posterior seguimiento en el ensayo clínico se propone el estudio del LCR.

Pasos a seguir:

1. Registro del paciente y seguimiento clínico

Para la inclusión en el protocolo de investigación, el paciente debe ser informado de la posibilidad de que su muestra sea almacenada para un posible uso posterior si fuera necesario en el desarrollo del proyecto.

Una vez el paciente accede, quedará registrado en una base de datos e incluido en un protocolo de diagnóstico y seguimiento clínico por parte del neurólogo, el cual habrá sido adoptado de forma consensuada por los profesionales clínicos que intervienen en el proyecto.

2. Consentimiento Informado y obligaciones legales

El paciente quedará vinculado al ensayo a través de un documento firmado que recoge sus datos personales y su firma.

Se dispondrá de protocolos de consentimiento informado para las pruebas específicas de diagnóstico que lo requieran, así como para determinaciones adicionales que sean necesarias para el seguimiento clínico del paciente.

Tanto el paciente como sus familiares podrán conocer en todo momento, si así lo solicitan, qué tipo de estudios se llevará a cabo con el material biológico que se le ha extraído.

3. Bases de datos: estructura y confidencialidad.

La base de datos de líquido cefalorraquídeo creada a tal efecto para el ensayo clínico, recogerá exhaustivamente los datos personales del paciente y de personas de contacto además de datos sobre los siguientes aspectos:

- Información sobre la procedencia de la muestra y condiciones de recogida
-Características de la muestra obtenida (calidad, volumen, celularidad)
- Diagnóstico y seguimiento clínico (Neurólogo responsable, identificación del Centro, Tf de contacto)
- Resultados de todos los estudios realizados con la muestra
-Datos sobre el tipo de muestras disponibles (volumen, nº de alícuotas)
-Información sobre el destino de las muestras (etiquetado y localización)

La base de datos será manejada por un único administrador responsable de la introducción y veracidad de los datos que serán utilizados en absoluta confidencialidad y estarán sometidos a los correspondientes controles informáticos de seguridad, siendo el acceso a ellos limitado a un número determinado de personas.

4. Obtención de la muestra

Para el estudio del LCR se necesitan tener muestras pareadas de suero y líquido del paciente.

Las muestras de líquido cefalorraquídeo serán extraídas al paciente por el profesional correspondiente utilizando siempre un recipiente adecuado a tal efecto (tubos esterilizados) y una vez firmado el consentimiento para la realización de la punción lumbar.

La muestra de suero se obtendrá mediante extracción de sangre normal en el mismo momento en que se realiza la punción lumbar.

Este procedimiento se realizará en las visitas de selección y en la del mes 1. Dependiendo de los resultados obtenidos en la analítica del LCR realizada en la visita del mes 1 se podrán realizar hasta un máximo de dos analíticas de LCR adicionales a lo largo del estudio.

5. Recepción de la muestra

La muestra será recogida en el laboratorio y será perfectamente identificada mediante:

- El número de identificación del paciente
- Código del estudio
- Fecha y hora de la extracción
- Cantidad total de muestra extraída

Las muestras recibidas del Hospital Carlos Haya deben ser enviadas en kits especiales preparados a tal efecto que serán enviados por el Hospital Virgen Macarena. Estas muestras se transportarán en cajas térmicas, manteniendo el frío mediante acumuladores pero nunca congelando las muestras.

Tratamiento previo al envío de muestras desde el Hospital Carlos Haya (Málaga).

LCR

Una vez obtenido el LCR se centrifugará a 4ºC durante 10 min a 3000 rpm (poner en g lo correspondiente). Seguidamente el sobrenadante se pasará a un tubo estéril especial que se enviara en el kit de muestra. El pellet de células será utilizado para los estudios de citometría de flujo que serán realizados para las muestras de los pacientes de Málaga en el Hospital Virgen Macarena

Bioquímica

La sangre se extraerá en un tubo de bioquímica normal que también se encontrará en el kit . Una vez obtenida la muestra de sangre, se centrifugará en frío (4ºC) a 2500 rpm durante 10 min. El sobrenadante (suero), se recoge con cuidado mediante una pipeta automática de 1ml con puntas estériles y se pasa a los tubos que a tal efecto se mandan en el kit.

Las muestras deberán ser entregadas en el Hospital Virgen Macarena antes que transcurran las 24 h desde su extracción. Las muestras del Hospital Virgen Macarena deben de ser recepcionadas en un tiempo inferior a las tres horas desde su extracción, condición absolutamente necesaria para llevar a cabo las determinaciones de las subpoblaciones linfocitarias de las muestras

A la recepción de la muestra en el laboratorio del HU Virgen Macarena se deberán comprobar los siguientes datos:

- Identificación correcta de la muestra

- El envase exterior está adecuadamente cerrado y no presenta daños o deterioro.
- La preparación del envío es correcta, en el envase especial autorizado para su transporte y manteniendo las condiciones de frío adecuadas.
- La muestra va acompañada de la documentación correspondiente del Hospital Universitario Carlos Haya u Hospital Universitario Virgen Macarena.

6. Procesamiento de muestras.

El procesamiento de las muestras biológicas se llevará a cabo de manera semiaséptica y con limpieza absoluta de los instrumentos y superficies de trabajo así como con la protección adecuada para el personal de laboratorio.

Una vez recibidas en el laboratorio, las muestras serán procesadas por especialistas que seguirán las siguientes pautas:

6.1. El LCR se centrifugará durante 10 min a 3000 rpm a 4ºC y el sobrenadante será recogido en tubos estériles. Las células serán utilizadas para el estudio de citometría de flujo.

6.2. La sangre se centrifugará a 2500 rpm a 4ºC durante 10 min. El sobrenadante se recogerá en un tubo estéril.

6.2. El LCR libre de células y el suero, se alicuotarán en volúmenes de 300 microlitros en tubos de congelación y se conservarán a -80ºC convenientemente etiquetadas y almacenadas en cajas identificadas con el registro del caso. Se registrará el número de alícuotas y la localización de cada una de las muestras con las correspondientes etiquetas identificativas del sujeto de ensayo.

Los frigoríficos congeladores serán monitorizados para controlar las posibles fluctuaciones de temperatura y se dispondrá de un sistema electrógeno de utilización en caso de caída del sistema eléctrico.

Según el protocolo del ensayo se llevará a cabo la recepción de dos muestras pareadas por paciente de estudio. Una al comienzo del ensayo clínico y otra al final del mismo (un año tras el comienzo del estudio).

Para cada una de las muestras se realizarán los siguientes análisis:

1. Contaje de la celularidad
2. Citometría de flujo
3. Determinación de BOCG y BOCM
4. Morfometría de las BOCG
5. Estudio de marcadores indicativos y que cuantifican la degeneración axonal en el LCR.
6. Estudio de los perfiles metabolómicos, de antigen arrays y de expresión génica.
7. En el mes 6 y con el fin de obtener datos de seguridad se determinará (Hospital Virgen Macarena) la carga del virus JC.

1.Contaje de la celularidad

2.Citometría de flujo

Para la determinación en el laboratorio de los apartados 1 y 2 se parte del pellet obtenido tras la centrifugación del LCR.

3. Estudio metabolómico mediante espectroscopia NMR

Se realizará con un NMR equipado con un imán de 9.4 T. Los espectros se procesan mediante software XWINNMR (Bruker) y transformación de Fourier. Los espectros se integran para obtener los patrones de reconocimiento mediante el uso del paquete SIMCA-P (Umetrics, Sweeden).

4. Perfiles de expresión génica mediante y de *antigen arrays*

5. Nefelometría

La cuantificación de las inmunoglobulinas G ,M, y A presentes en el LCR y suero de los pacientes se llevará a cabo en el laboratorio de inmunología del Hospital Universitario Virgen Macarena, mediante técnicas nefelométricas.

6. Determinación de BOCG y BOCM

Determinación de BOCG

La técnica desarrollada en el Hospital Virgen Macarena es la que más alta sensibilidad y especificidad ha demostrado hasta el momento. Basada en la técnica de Sádaba MC *et al* (Sádaba MC, González Porqué P, Masjuan J, Álvarez-Cermeño JC, Bootello A, Villar LM. An ultrasensitive method for the detection of oligoclonal IgG bands. Journal of Inmunological Methods 284 (2004) 141-145). Se han mejorado algunos aspectos de la misma para obtener un protocolo propio del laboratorio.

La detección de BOC de IgG se realiza mediante el siguiente protocolo:

a) Preparación del gel de agarosa. Diluir 2,5mL de Glicerol en 30mL de agua destilada en erlenmeyer de 250mL. Pesar 3,6g de Sorbitol y añadirlo a la mezcla, mezclar con suave agitación hasta su disolución. Pesar 0,3g de Agarosa IEF y añadirlo a la mezcla. Calentar hasta disolver y preparar el gel con cuidado para que no polimerice antes de añadirlo al soporte donde se realizará el isoeletroenfoque.

b) Isoelectroenfoque: mediante una cubeta de electroforesis con placa refrigerante (Multiphor II system; Pharmacia Biotech), acoplada a una fuente de alimentación (Electrophoresis Power Supply-EPS 3501; Amersham Pharmacia Biotech) y a un compresor de refrigeración (LKB DROMMA 2219 MULTITEMP II Thermostatic circulator). Se carga la misma concentración de ambas muestras a partir de los datos de cuantificación obtenidos mediante Nefelometría en el laboratorio de Inmunología del Hospital Virgen Macarena.

c) Tras la separación, las proteínas se transfieren por capilaridad a una membrana de nitrocelulosa (PALL). El bloqueo de los sitios de unión que permanecen libres, se realiza mediante incubación de la membrana al menos 1 hora a temperatura ambiente, con agitación, en una solución de leche en polvo (SVELTESSE) en suero fisiológico, al 2 % (p/v).

d) La inmunodetección de las BOC se realiza mediante la incubación de la membrana en una solución de suero fisiológico y leche en polvo al 0.02% que contiene anticuerpos de cabra específicos frente a las dos cadenas de IgG humana conjugado con fosfatasa alcalina, durante toda la noche a 4 ºC y en agitación.

e) Seguidamente se realiza la detección colorimétrica de la fosfatasa alcalina, usando como sustrato NBT y BCIP (SIGMA-ALDRICH) que da lugar a la aparición de un precipitado de color morado.

Determinación de BOCM.

La determinación de las BOCM se realizan con el mismo sistema utilizado para la determinación de las BOCG, tan sólo varía el tratamiento previo de la muestra así como el programa para el desarrollo de las muestras guante el isoqlectroenfoque.

a) Tratamiento de las muestras: Tanto los LCR como las diluciones de suero han de digerirse con una mezcla de DTT 500 mM y Tris/HCl 1M, pH: 9,5. Se tratan 20µL de LCR y 20 µL de dilución de suero.

DTT: 0,077g DTT/ 10mL de H2O

Tris/HCl: 1,21g Tris-base + 8mL de H2O; ajustar con HCl hasta pH: 9,5 y después enrasar con H2O hasta 10mL.

Preparar estas dos soluciones el mismo día que van a ser utilizadas ya que se obtienen mejores resultados.

b) Isoelectroenfoque: Cargamos 3,8µL de dilución de suero y 10µL de LCR (los dos tratados ya con DTT y Tris/HCl), para realizar la electroforesis con la misma cantidad de IgM de cada muestra.

c) Inmunodetección. El anticuerpo utilizado es anti-IgM humana de cabra marcado con fosfatasa alcalina.

5. Morfometría de las BOCG

Para el estudio el paciente firmará un consentimiento en el que conocerá la utilización de los resultados por parte del investigador para el análisis morfométrico del mismo.

Tras la obtención de la membrana se llevará a cabo su digitalización y posterior almacenamiento informático. Este sistema informatizado de imágenes permitirá realizar estudios tanto prospectivos como retrospectivos, pues una vez escaneadas o fotografiadas, las membranas de nitrocelulosa permanecen almacenadas en ficheros individuales utilizables en cualquier momento y sin pérdida de nitidez ni deterioro de las imágenes.

Ingeniería biomédica: análisis de las BOG mediante software de morfometría

Una vez digitalizadas las membranas, los resultados obtenidos para cada uno de los pacientes se analizarán mediante un software desarrollado por el Grupo de Investigación "*Física Interdisciplinar. Fundamentos y Aplicaciones*" (GFI) de la E.S. Ingenieros de la Universidad de Sevilla, dirigido por el Dr. Emilio Gómez González, como especialistas en técnicas de visualización y procesado de imágenes en aplicaciones médicas. El programa permite procesar las imágenes de BOC con una mínima o nula intervención del usuario, de manera que los resultados del análisis óptico digital y morfometría de las bandas son reproducibles y objetivos.

De los resultados obtenidos se extrae información de la posición y la intensidad de las BOCG pudiéndose comparar perfiles entre pacientes que pudiéramos relacionar con la evolución del apaciente a lo largo del ensayo clínico.

Método

Se considera *caso de estudio* a la imagen (captada como fotografía digital o scaner) de bandas de cada paciente en cada instante de tiempo. Partiendo de la imagen completa (*membrana*) se selecciona una pareja de bandas de cada caso, mediante un módulo estándar de recortado, generándose una imagen (*imagen del caso*) que consta de dos imágenes parciales, una *banda de suero* ("*banda control*") y una *banda de análisis* ("*banda problema*").

La imagen del conjunto de las bandas (*membrana*) es registrada una vez digitalizada, en escala de grises, con una profundidad mínima de color de 8 bpp. En los extremos iniciales de las bandas se marca, previamente, una línea transversal (de la misma longitud en cada banda) que define la anchura de las mismas. Las diferentes bandas pueden tener así distinta longitud pero siempre tienen todas la misma anchura (*anchura de referencia*, A_o). La longitud máxima alcanzable por una banda se define como *longitud de referencia* (L_o).

De cada caso, mediante el programa específico de análisis, se procesan las dos imágenes parciales (*banda de suero* y *banda de análisis*) por separado, aplicándose algoritmos de morfometría fundamentados en análisis estadístico y morfológico para la detección, localización y caracterización de los *hallazgos o franjas* presentes en las bandas. En la *banda de suero* se determinan su longitud (L_1), y un total de 10 parámetros estimadores de imagen de cada franja (incluyendo área, densidad óptica media, desviación estándar, mediana y otros). En la *banda problema* se determinan asimismo su longitud (L_2), la posición (centro y anchura) y 10 estimadores ópticos de cada franja presente en la banda. Los parámetros geométricos de ambas bandas se normalizan respecto a la longitud de referencia y los relacionados con la densidad óptica se normalizan respecto a la densidad óptica media de la *banda de suero*, que se toma como referencia (propia de cada caso), para obtener valores (relativos) que puedan compararse entre los diferentes casos.

El análisis estadístico (mediante tests estándar de Student, Cohen y otros) se utilizará para caracterizar el agrupamiento o dispersión de los valores de cada estimador para los casos de una misma patología y cuantificar su esperada diferenciación con los casos correspondientes a otras patologías. Este análisis puede llevarnos, asimismo, a definir nuevos parámetros (o nuevas combinaciones de los anteriores) para la caracterización de las franjas. Los parámetros iniciales del sistema son así la resolución de la imagen digital de las bandas y la longitud y anchura de referencia de las mismas.

El software ha sido validado por nuestro grupo mediante el estudio de un grupo de entrenamiento (training set) y un grupo posterior de prueba (test set) comprobando la fiabilidad y sensibilidad del programa para detectar la posición de cada una de las franjas (bandas) correspondientes a los resultados de la determinación de BOCG.

6. Estudio de marcadores indicativos de la degeneración axonal en el LCR.

Los sujetos que van a formar parte de este ensayo clínico, presentan una clara degeneración axonal que han ido adquiriendo a medida que la enfermedad ha ido evolucionando. Durante el ensayo clínico, la RM será la prueba paraclínica utilizada para observar en qué medida evoluciona favorablemente la degeneración axonal de partida tras el tratamiento con las células madre.

A parte de la RM, en el LCR se encuentran otra serie de marcadores indicativos de este hecho y que serán determinados en las muestras como apoyo al estudio evolutivo del paciente desde el comienzo del ensayo clínico hasta su final, de manera que se pueda evaluar de la manera más específica posible la factibilidad del tratamiento mediante células madres.

Se realizará el estudio de:

La proteína TAU y TAU fosforilada: La cuantificación se llevará a cabo mediante inmunoensayos con anticuerpos monoclonales con reconocimiento específico de proteínas Tau (anti-Tau), mediante técnicas de ELISA (Innotest hTAU Ag).

Determinación de β-amiloide: La cuantificación se llevará a cabo mediante inmunoensayos con anticuerpos monoclonales con reconocimiento específico del fragmento β1-42 de amiloide, mediante técnicas de ELISA (Innotest Aβ1-42).

ANEXO XI: CUADERNO DE RECOGIDA DE DATOS

Se adjunta

Normas para la cumplimentación de losCRD

Cada investigador se compromete a:

- Registrar al paciente tan pronto como se incluya al enfermo en el estudio.
- Rellenar las hojas de recogida de datos de cada paciente.

Para la correcta cumplimentación de las hojas se debe tener en cuenta:

- Las hojas en que se indica, deberán ir fechadas y firmadas por el investigador principal autorizado.
- Cumplimentará todas las casillas. Si no dispone del dato que se le pide, deberá poner ND (no disponible).
- Los resultados poco usuales o extremos, o las que no concuerden con la secuencia esperada, deberán comprobarlos. Serán corregidos poniendo sus iniciales, la firma y la explicación correspondiente.
- Los resultados de laboratorio que excedan los rangos de normalidad establecidos por el laboratorio del centro, deberán ser comprobados por el investigador y su significado se anotará al lado del dato, poniendo sus iniciales y la firma.
- Procurará escribir las preguntas abiertas con letra legible, pues en caso contrario se considerará como información perdida.
- El investigador realizará una comprobación o revisión final de todos y cada uno de los CRD.
- Los datos deben ser recogidos con bolígrafo de color negro.

ANEXO XII: DECLARACIÓN DE HELSINKI DE LA ASOCIACIÓN MÉDICA MUNDIAL (WMA)

Principios éticos para las investigaciones médicas en seres humanos
Adoptada por la 18ª Asamblea general de la WMA, Helsinki, Finlandia, Junio 1964
y enmendada por la
29ª Asamblea general de la WMA, Tokio, Japón, Octubre 1975
35ª Asamblea general de la WMA, Venecia, Italia, Octubre 1983
41ª Asamblea general de la WMA, Hong Kong, Septiembre 1989
48ª Asamblea general de la WMA, Somerset West, República de Sudáfrica, Octubre 1996
y la
52ª Asamblea general de la WMA, Edimburgo, Escocia, Octubre 2000
Nota de clarificación del Párrafo 29 agregada por la Asamblea general de la WMA, Washington 2002
Nota de clarificación del Párrafo 30 agregada por la Asamblea general de la WMA, Tokio 2004

A. INTRODUCCIÓN

1. La Asociación Médica Mundial ha promulgado la Declaración de Helsinki como una propuesta de principios éticos que sirvan para orientar a los médicos y otros integrantes que realizan investigación médica en seres humanos. La investigación médica en seres humanos incluye la investigación de material humano identificable o de datos identificables.

2. El deber del médico es promover y velar por la salud de las personas. Los conocimientos y laconciencia del médico han de subordinarse al cumplimiento de ese deber.

3. La Declaración de Ginebra de la Asociación Médica Mundial vincula al médico con las palabras "La salud de mi paciente será mi principal prioridad ", y el Código Internacional de Ética Médica afirma que: "El médico debe actuar solamente en el interés del paciente al proporcionar atención médica que pueda tener efectos de debilitantes sobre la condición mental y física del paciente".

4. El progreso de la medicina se basa en la investigación, la cual, en último término, tiene que recurrir muchas veces a la experimentación en seres humanos.

5. En investigación médica en seres humanos, la preocupación por el bienestar de los seres humanos debe tener siempre primacía sobre los intereses de la ciencia y de la sociedad.

6. El propósito principal de la investigación médica en seres humanos es mejorar los procedimientos preventivos, diagnósticos y terapéuticos, y también comprender la etiología y patogenia de las enfermedades. Incluso, los mejores métodos preventivos, diagnósticos y terapéuticos disponibles deben ponerse a prueba continuamente a través de la investigación para que sean eficaces, efectivos, accesibles y de calidad.

7. En la práctica de la medicina y de la investigación médica del presente, la mayoría de los procedimientos preventivos, diagnósticos y terapéuticos implican algunos riesgos y costos.

8. La investigación médica está sujeta a normas éticas que promueven el respeto a todos los seres humanos y protegen su salud y sus derechos. Algunas poblaciones sometidas a la investigación son vulnerables y necesitan protección especial. Se deben reconocer las necesidades particulares de los que tienen inferioridad económica y médica. También se debe prestar atención especial a los que no pueden otorgar o rechazar el consentimiento por sí mismos, a los que pueden otorgar el consentimiento bajo presión, a los que no se beneficiarán personalmente con la investigación y a los que tienen la investigación combinada con la atención médica.

9. Los investigadores deben conocer los requisitos éticos, legales y jurídicos para la investigación en seres humanos en sus propios países, así como los requisitos internacionales vigentes. No se debe permitir que un requisito ético, legal o jurídico disminuya o elimine cualquier medida de protección para los seres humanos establecida en esta Declaración.

B. PRINCIPIOS BÁSICOS PARA TODA INVESTIGACIÓN MÉDICA

10. En la investigación médica, es deber del médico proteger la vida, la salud, la intimidad y la dignidad del ser humano.

11. La investigación médica en seres humanos debe conformarse con los principios científicos generalmente aceptados, y debe apoyarse en un profundo conocimiento de la bibliografía científica, en otras fuentes de información pertinentes, así como en experimentos de laboratorio adecuados, incluso en animales, cuando se considere oportuno.

12. En las investigaciones, hay que prestar atención adecuada a los factores que puedan perjudicar el medio ambiente y el bienestar de los animales utilizados en los experimentos.

13. El diseño y el método de todo procedimiento experimental en el que haya implicación de seres humanos deberá formularse claramente en un protocolo experimental. Este debe enviarse, para consideración, comentario, consejo, y cuando sea oportuno, aprobación, a un comité de evaluación ética especialmente designado, que debe ser independiente del investigador, del patrocinador o de cualquier otro tipo de influencia impropia. Este comité independiente deberá actuar en conformidad con las leyes y reglamentos vigentes en el país donde se realiza la investigación experimental. El comité tiene el derecho de controlar los ensayos en curso. El investigador tiene la obligación de proporcionar la información de control al comité, especialmente cualquier evento adverso grave. El investigador también debe presentar al comité, para que la revise, la información sobre financiación, patrocinadores, afiliaciones institucionales, otros posibles conflictos de interés e incentivos para los participantes.

14. El protocolo de la investigación deberá hacer referencia siempre a las consideraciones éticas que pueda entrañar el estudio, y deberá indicar la conformidad con los principios enunciados en esta Declaración.

15. La investigación médica en seres humanos deberá ser llevada a cabo sólo por personas científicamente calificadas y bajo la supervisión de un médico clínicamente competente. La responsabilidad de los seres humanos deberá recaer siempre en una persona con capacitación médica, y nunca en los sujetos de la investigación, aunque éstos hayan otorgado su consentimiento.

16. Todo proyecto de investigación médica en seres humanos deberá ser precedido de una minuciosa valoración de los riesgos previstos con los beneficios predecibles para el individuo o para otros. Esto no impide la participación de voluntarios sanos en la investigación médica. El diseño de todos los estudios deberá ser de disponibilidad pública.

17. Los médicos deberán abstenerse de participar en proyectos de investigación en seres humanos a menos que estén seguros de que los riesgos inherentes hayan sido adecuadamente evaluados y de que es posible afrontarlos de manera satisfactoria. Los facultativos deberán suspender la investigación en curso si observan que los riesgos que implican rebasan los beneficios esperados o si existen pruebas concluyentes de resultados positivos o beneficiosos.

18. La investigación médica en seres humanos únicamente deberá realizarse cuando la importancia de su objetivo supera el riesgo inherente y los costos para el individuo. Esto es especialmente importante cuando los seres humanos son voluntarios sanos.

19. La investigación médica sólo se justifica si existen posibilidades razonables de que la población sobre la que se realiza la investigación vaya a beneficiarse de sus resultados.

20. Para tomar parte en un proyecto de investigación, los individuos deberán ser voluntarios o participantes informados.

21. Siempre deberá respetarse el derecho de los sujetos de la investigación a proteger su integridad. Deben tomarse toda clase de precauciones para respetar la intimidad de los

individuos, la confidencialidad de la información del paciente y para reducir al mínimo las consecuencias del estudio sobre su integridad física y mental y su personalidad.

22. En toda investigación en seres humanos, cada sujeto potencial deberá estar debidamente informado acerca de los objetivos, métodos, fuentes de financiación, posibles conflictos de intereses, afiliaciones institucionales del investigador, beneficios esperados y riesgos previsibles del estudio y las molestias que pueda conllevar. El sujeto deberá ser informado del derecho de participar o no en la investigación y de retirar su consentimiento en cualquier momento, sin sufrir represalias. Después de asegurarse de que el individuo ha comprendido la información, el médico deberá obtener entonces el consentimiento informado y voluntario de la persona preferiblemente por escrito. Si el consentimiento no pudiera obtenerse por escrito, el proceso para lograrlo deberá estar formalmente documentado y atestiguado.

23. Al obtener el consentimiento informado para el proyecto de investigación, el médico deberá poner especial cuidado si el individuo está vinculado a él por una relación de dependencia o si consiente bajo presión. En tal caso, el consentimiento informado deberá ser obtenido por un médico bien informado que no participe en la investigación y que nada tenga que ver con dicha relación.

24. Cuando el sujeto sea legalmente incompetente, incapaz física o mentalmente de otorgar su consentimiento, o legalmente menor de edad, el investigador deberá obtener el consentimiento informado del representante legalmente autorizado y conforme a la legislación vigente. Estos grupos no deberán ser incluidos en la investigación a menos que el estudio sea necesario para mejorar la salud de la población representada y no sea posible realizar la investigación en personas legalmente capaces.

25. Si una persona considerada legalmente incompetente, como es el caso de un menor de edad, es capaz de dar su conformidad a participar o no en la investigación, el investigador deberá obtenerlo, además del consentimiento de su representante legal.

26. La investigación en individuos de los que no se puede obtener consentimiento, incluso por representante o con anterioridad, se deberá realizar únicamente si la condición física/mental que impide obtener el consentimiento informado es una característica necesaria de la población investigada. Las razones concretas por las que se utilizan sujetos en la investigación con una dolencia que les impide otorgar su consentimiento informado deben ser concretadas en el protocolo experimental que se presenta para la consideración y aprobación del comité de evaluación. El protocolo deberá establecer que el consentimiento para permanecer en la investigación deberá obtenerse con la mayor brevedad posible del individuo o de un representante legal.

27. Tanto los autores como los editores tienen obligaciones éticas. Con la publicación de los resultados de la investigación, los investigadores están obligados a mantener la exactitud de los resultados. Se deberán publicar tanto los resultados negativos como los positivos o estar a disposición pública en modo alguno. En la publicación se deberán citar las fuentes de financiación, afiliaciones institucionales y cualquier posible conflicto de intereses. Los informes sobre cualquier tipo de experimentación no conforme a los principios descritos en la presente Declaración no deberán ser aceptados para su publicación.

C. PRINCIPIOS ADICIONALES SI LA INVESTIGACIÓN MÉDICA SE COMBINA CON LA ATENCIÓN MÉDICA

28. El médico puede combinar la investigación médica con la atención médica, sólo en la medida en que tal investigación acredite un justificado valor potencial preventivo, diagnóstico o terapéutico. Cuando la investigación médica se combina con la atención médica, se aplican normas adicionales para proteger a los pacientes que participan en la investigación.

29. Los beneficios, riesgos, costos y efectividad de todo procedimiento nuevo deberán ser evaluados mediante su comparación con los mejores métodos preventivos, diagnósticos y terapéuticos existentes. Esto no excluye el empleo de un placebo o ausencia de tratamiento, en estudios para los que no existen métodos preventivos, diagnósticos o terapéuticos probados. [1]

30. Al final del estudio, todos los pacientes que participen en el mismo deberán tener garantizado el acceso a los mejores métodos preventivos, diagnósticos y terapéuticos identificados por el estudio. [2]

31. El médico deberá informar puntualmente al paciente sobre los aspectos de la asistencia que tengan relación con la investigación. La negativa del paciente a participar en un estudio nunca deberá perturbar la relación médico-paciente.

32. Cuando en el tratamiento de un paciente los métodos preventivos, diagnósticos o terapéuticos probados no existen o han resultado ineficaces, el médico, con el consentimiento informado del paciente, deberá tener la libertad de emplear procedimientos preventivos, diagnósticos y terapéuticos nuevos o no contrastados, si, a su juicio, ello ofrece esperanza alguna de salvar la vida, restituir la salud o mitigar el sufrimiento. Siempre que sea posible, tales medidas deberán ser el objeto de la investigación, que estará diseñada con el fin de evaluar su seguridad y factibilidad. En todos los casos, cualquier información nueva deberá ser registrada y, cuando sea oportuno, publicada. Se deberán observar todas las demás normas pertinentes de la presente Declaración.

[1] Nota de Clarificación del Párrafo 29 de la Declaración de Helsinki de la WMA

Por la presente, la WMA reafirma que se deberá extremar el cuidado al emplear ensayos controlados por placebo y, en general, esta metodología se deberá emplear únicamente en la ausencia de una terapia probada. Sin embargo, los ensayos con placebo pueden ser aceptables éticamente, incluso si se dispone de una terapia probada, en las siguientes circunstancias:

- Cuando por razones metodológicas, científicas y apremiantes, su uso es necesario para determinar la eficacia o la seguridad de un método preventivo, diagnóstico o terapéutico o;

- Cuando se prueba un método preventivo, diagnóstico o terapéutico para un trastorno leve, siempre que no suponga un riesgo adicional de daños graves o irreversibles para los pacientes a los que se administra el placebo.

Se deberán observar todas las demás disposiciones de la Declaración de Helsinki, en especial la necesidad de una revisión científica y ética apropiada.

[2] Nota de Clarificación del Párrafo 30 de la Declaración de Helsinki de la WMA

Por la presente, la AMM reafirma su postura de que durante el proceso de planificación del estudio será necesario identificar el acceso después del ensayo de los participantes del estudio a procedimientos preventivos, diagnósticos y terapéuticos que han demostrado ser beneficiosos en el estudio o el acceso a una atención alternativa adecuada. Los arreglos para el acceso después del ensayo o la atención alternativa deberán estar descritos en el protocolo del estudio, de manera que el comité de revisión ética pueda considerar dichos arreglos durante su revisión.

ANEXO XIII: HOJA DE INFORMACIÓN Y CONSENTIMIENTO INFORMADO PARA EL PACIENTE

Se adjunta

ANEXO XIV: CERTIFICADO DE LA PÓLIZA DE SEGURO

Se adjunta
