# Supplementary material for: Adipose-derived mesenchymal stem cells (AdMSC) for the treatment of secondary-progressive multiple sclerosis: A triple blinded, placebo controlled, randomized phase I/II safety and feasibility study
Source: PLoS One. 2018 May 16;13(5):e0195891. doi: 10.1371/journal.pone.0195891 (PMC5955528; doi:10.1371/journal.pone.0195891)
Supplement: S4 File — (DOCX) [file pone.0195891.s004.docx]

**Autologous Mesenchymal Stem Cells From Adipose Tissue in Patients With Secondary Progressive**

**Multiple Sclerosis**

**ClinicalTrials.gov Identifier:NCT01056471**

**EudraCT Number: 2008-004015-35**

# CLINICAL TRIAL: TYPE AND DESIGN

## DEVELOPMENT PHASE

This is a multicenter, randomized, double-blind, placebo-controlled clinical trial phase I / II of therapy with autologous adipose tissue-derived mesenchymal stem cells (AdMSC). The goal of the study is to evaluate the safety, feasibility and possible clinical benefits of the intravenous administration of two doses of AdMSC in patients with Secondary Progressive Multiple Sclerosis (MS) who do not respond to conventional therapies.

## GLOBAL STUDY DESIGN

## Design

A prospective, multicenter, double-blinded, randomized, placebo-controlled study with two experimental doses of autologous mesenchymal stem cells from adipose tissue.

## Dosage and treatment regimen:

Study population will consist of 30 clinically evaluable patients with Secondary Progressive MS with treatment failure. Distribution of the patients was divided in 3 arms:

- **Arm 1 - Placebo Group:** 10 patients that will receive Placebo (exclusively the excipients of the cell suspension) in the same conditions as the experimental groups. The placebo will be administered at the basal visit.
- Composition of the placebo will be:
  - - Ringer's Lactate solution:
      - - Calcium chloride dihydrate (2,7 mg/100ml)
        - Sodium lactate (320 mg/100ml)
        - Potassium chloride (40 mg/100ml)
        - Sodium chloride (600 mg/100ml)
    - 2,5% glucose
    - 1% Human serum albumin.
- **Arm 2 - Experimental Group 1:** 10 patients that will receive a suspension of 1·10^6^ autologous mesenchymal stem cells/kg of weight (in the excipients described for the placebo group), in a single dose by a slow intravenous infusion, at the basal visit of the study.
- **Arm 3 - Experimental Group 2:** 10 patients that will receive a suspension of 4·10^6^ autologous mesenchymal stem cells/kg of weight (in the excipients described for the placebo group), in a single dose by a slow intravenous infusion, at the basal visit of the study.

The inclusion in each of the 3 arms of the study will be random until reaching the size of the sample (30 patients clinically evaluable), maintaining a balanced distribution of patients in each group.

On the other hand, the study will be discontinued if some of the circumstances contemplated as a reason for interrupting the trial are presented.

## Expected duration of participation of subjects. Sequence and periods of the trial.

The period of inclusion of patients ranges from 12 to 24 months. The follow-up period of the patients will be 12 months and will be carried out in 8 visits according to the proposed scheme (see Annex I), therefore the 30 patients will be evaluated longitudinally for all clinical and paraclinical parameters.

The study ends at 12 months of follow-up of the last treated patient but, since these patients are seen routinely in the Neurology Departments of the participating centers in the study, data will be collected at 24 months to obtain additional safety and feasibility information in the medium term, with a specific protocol.

**During the 12-month visit (or the routine visit at 24 months) and, provided that the feasibility and safety results allow it, infusion of adipose-derived mesenchymal stem cells to those patients previously randomized in the control group will be performed, requesting an extension of the study for such procedure.**

## EVALUATION METHODS

## Clinical evaluation

A neurologist will carry out the preinclusion visit of each patient and will verify the suitability of the candidate who must meet the inclusion and exclusion criteria in the project. The appropriate patient will be properly informed and will have to sign the written consent. A clinical history will be performed to each patient, as well as a general physical and neurological examination, and a specific evaluation of MS, with the Expanded Disability Scale Score of Kurtzke (EDSS) and Multiple Sclerosis Functional Composite (MSFC) (See Annex II). The history of relapses and the progression of the disease in the previous years will be evaluated.

## Adipose tissue collection

If the patient is included, in the preinclusion period, a minimum of 30 grams of clean fatty tissue from the abdominal wall will be collected by aseptic plastic surgery techniques, and will be sent at 2-8ºC in a transport kit with a data logger recording temperature continuously, to the GMP laboratory for further processing.

## Vasculo Stromal Fraction Isolation and Expansion of adipose tissue-derived mesenchymal stem cells (AdMSC)

# It will be performed at the GMP facility at the CABIMER Institute, Seville.

**Tecnical Director responsible of the Unit:**

Particia Gálvez Martín

Centro Andaluz de Biología Molecular y Medicina Regenerativa (CABIMER)

Avda, Américo Vespucio s/n – Edif. CABIMER

Parque Científico y Tecnológico Cartuja 93

41092 Sevilla

España

Tel/Phone:+34 954 468 004

Fax:+34 954 461 664

www.cabimer.es

## Functional analysis of the Immune Response

# It will be performed according to the procedure described in Annex IXb by:

Dr. David Pozo Perez

Centro Andaluz de Biología Molecular y Medicina Regenerativa - CABIMER

Avda, Américo Vespucio s/n – Edif. CABIMER

Parque Científico y Tecnológico Cartuja 93

41092 Sevilla

España

Tlf: 95-4467841 / 95-4467403; Fax: 95-4461664; Email.: [david.pozo@cabimer.es](mailto:david.pozo@cabimer.es)

## Feasibility parameters

- Clinical variables
  - **Number of relapses** (defined according to McDonald's criteria)
  - **Progression** (scores in EDSS and MSFC)
- Paraclinical variables
  - **Magneting Resonance Imaging:** the MRI study will be performed in a in a high field 1.5T equipment, using the following different sequences for the analysis of lesions (see Annex III):

- Localization sequences

- Transverse sequence in proton density / T2 (DP / T2)

- FLAIR cross-sectional

- T1-weighted transverse spin-echo sequence

- Transverse spin-echo sequence enhanced in T1 with Gadolinium

- Isotropic diffusion sequence

- Magnetization Transfer

- Proton Hydrogen Spectroscopy

- **Evoked potentials** (visual, acoustic, somatosensitive, cognitive, motor, EPAS scale). The evoked potentials will be obtained with an electromyograph with software for evoked potentials with a four-channel amplifier. Surface electrodes shall be used for collecting the potentials. The methodology applied will be that of the published guidelines (Deuschl G, Eisen A. Recommendations for the practice of clinical neurophysiology: Guidelines of the International Federation of Clinical Neurophysiology, Electroencephalog. Clin Neurophysiol 1999, 52 (Suppl): 192-211) and will be compared with the normal values obtained in our laboratories (see Annex IV).
- **Optic Coherence Tomography**. The test will be performed using a OCT device, making two 5 mm-cuts in each patient, one on the vertical axis and the other on the horizontal, focused on the fovea. The patient will be instructed to fix the sight in the internal light in case of low visual acuity. The "scan" locates on the foveal area, visualized through the monitor assisted by the auxiliary external fixation of the contralateral eye. The images will be normalized and aligned using the software of the device. Measurement of intraocular pressure and pharmacological pupil dilation will be performed prior to the test.
- **Neuropsychological tests,** (BRB batterie and Wechsler intelligence scale for adults). Neuropsychological tests will be administered written to patients who will perform them in the presence of a neuropsychologist who will explain the test and will use the essential support tools, such as a tape recorder in PASAT (See Annex V).
- **Quality of Life Scles** (scales SF-36, Euroqol-5D, MusiQol). They will be completed by patients, under the supervision of a neuropsychologist (see Annex VI), or by a relative or a person related to the patient who literally conveys the patient's impressions in the questionnaire
  - Relapses of patients will be assessed at all visits except at the baseline visit.
  - The EDSS scale will be evaluated at the pre-inclusion visit, during the visit of the first month and in all subsequent visits.
  - The MSFC scale will be evaluated in the preinclusion visit, during the 90-day visit and in all successive visits.
  - MRI will be performed at the preinclusion visit, at 6 months and at 12 months.
  - The evoked potentials will be evaluated at the preinclusion visit, at 6 months and at 12 months.
  - The tomography will be performed at the preinclusion visit, at 6 months and at 12 months.
  - Neuropsychological tests will be evaluated at the preinclusion visit, at 6 months and at 12 months.
  - The quality of life tests will be completed at the preinclusion visit, at 6 months and at 12 months.

## Safety parameters:

The occurrence, intensity, and relationship to the study drug of adverse events and serious adverse events will be assessed at all study visits. In addition to pharmacovigilance, the following parameters shall be taken into account as safety measures:

- Laboratory analysis (haematological & biochemical)

- Immunological analysis

- EKG

- Chest x-ray

- Functional Respiratory Test

- CSF analysis

All of these variables will be measured at the preinclusion visit, the laboratory analysis will be repeated at all study visits except for the visit at day 1 and the visit of the first week. Immunological analysis will be repeated at the 90-day visit and at the first year of follow-up.

The EKG, chest X-ray and FRT will be performed in addition to the pre-inclusion visit, at 90 days and at 12 months. The CSF analysis will be carried out at the preinclusion visit or visit 1, at the visit of month 1 and at 12 months of follow-up.

## MASKING TECHNIQUES

This is a double blind study, regarding the treatment administered to the patients, where neither the patient nor the investigators know whether the patient receives placebo or treatment, nor the dose administered. The person in charge of analyzing the statistical data does not know the dose of each result either.

To allow for the unbiased evaluation of the medication, it will be evaluated through a three-arm, double-blind, randomized, placebo-controlled study.

The following methods will be used to maintain the blindness for patients, researchers, and for persons in charge of the statistical analysis of results:

- Removal of adipose tissue to both the control group and the experimental groups.
- Concealed coding of transplanted autologous cells or placebo, for investigators, patients and persons in charge of the statistical analysis of results.
- Randomization of the patients will be done centrally in the CABIMER according to the treatment to be administered to each patient (control, experimental group 1 and experimental group 2). The allocation of each patient to each arm of the study will be stored in a perfectly closed envelope and identified for each patient where, apart from coding, instructions will be included with the procedure to be followed in case it is necessary to reveal the blind. These envelopes will be attached to the study medication and will be stored in the researcher's file in the study medication section.
- Restricted access to knowledge regarding study medication (autologous mesenchymal cells or placebo) prescribed to a specific patient. Researchers will have access, to the name of the masked medications administered only to the patients under their responsibility. Researchers and other authorized persons will not open the treatment code unless it is absolutely necessary to distinguish the type of treatment received by a given patient in order to choose between crucial therapeutic options.
- None of the supervisory committees will have access to the list of treatment codes assigned to patients, except authorized persons, who will be responsible for overseeing all safety aspects of the study. The unmasked documentation will remain confidential and will not be available to anyone outside the trial.

Study treatments will be assigned using a balanced, sequential central randomization with a stratification factor: center. To achieve an approximate treatment balance within each stratum, a block randomization pattern of 3 patients with a 2: 1 allocation will be used. Each patient entering the study will have a patient number and according to this list will belong to the control group or to each of the two experimental groups of the study.

# SUBJECT SELECTION

## STUDY POPULATION

Secondary progressive Multiple Sclerosis Patients that do not respond to conventional registered therapies.

## INCLUSION CRITERIA

1. 18 Years and older, both gender
2. Patients diagnosed with clinically definite Multiple Sclerosis (Poser and McDonald criteria).
3. Secondary progressive MS patients with EDSS ≥ 5.5 and ≤ 9.
4. Patients with treatment failure defined by: no response to immunomodulators / immunosuppressants, and showing activity in the form of 1 relapse in the last year or progression of ≥ 0.5 points in EDSS scale.
5. Patients with no MS relapse and no steroid treatment within the month prior to inclusion.
6. Patients who give written consent to participate in the study. -

## EXCLUSION CRITERIA

1. History of current pathology or current laboratory results indicative of any severe disease.
2. Pacemaker or metallic implants that prevent MR imaging.
3. Refusal to give informed consent.
4. Predicted impossibility for a biopsy of at least 30 grams of fat tissue.
5. Positive screening test for HIV, Hepatitis B or Hepatitis C.
6. History of malignancy or current neoplasia.
7. Having been in treatment with any investigational drug or have undergone any experimental procedure in the 3 months prior to baseline.
8. Body mass index> 40 kg/m2.
9. Patients who have been treated with prohibited concomitant medication during the month prior to inclusion in the study.
10. Pregnancy or lactation
11. Inability to complete questionnaires.

## WITHDRAWAL CRITERIA AND THE PLANNED STATISTICAL ANALYSIS OF THE WITHDRAWALS AND DROPOUTS

Patients will be discontinued from the trial in any of the following situations:

1. An insufficient amount of adipose tissue is obtained (<30 gr).
2. In the event of not achieving the optimal final concentration of the cellular suspension, the investigator will decide on its administration, although the patient will be excluded from the feasibility/safety analysis, after informing the patient and obtaining the authorization for such procedure.
3. Serious adverse event previous to the mesenchymal cells infusion.
4. Clinical conditions of the patient that prevent its continuity.
5. Grade IV toxicity on the WHO adverse event scale (see Annex VII).
6. Any criteria from the Safety Committee that indicates the end of the study due to safety issues.

Furthermore, patients can be withdrawal in case of:

- Patient fails to cooperate or does not fulfil the study requirements.
- Investigator considers that the patient health is compromised due to adverse events, concomitant diseases or any other circumstances that arise during the study*.
- Adverse event(s)*.
- Abnormal laboratory finding(s)*.
- Abnormal result(s) of the test procedures*.
- Violation of the protocol.
- Patient withdraws his/her consent.
- Loss of patient´s follow up

*Once the investigational product has been administered (a single dose will be administered) to the patient, patient withdrawing from the trial will not grant any benefit regarding safety, so the patient will be advised to remain in the trial.

If the investigator stops hearing about a patient, all the efforts have to be made to get in touch with him/her (unless the patients had expressed that he/she does not wish to be contacted) in order to obtain the date on which the patient´s treatment was withdrawn, identify the reason of withdrawal, ask the patient to resume the study procedures or ask the patient to attend a last visit and recommend him/her to provide the contact details of the doctor who will follow his/her case.

If all the attempts to get in touch with the patient fail, the investigator will be asked to document all the actions implemented in the clinical record and, if the key study data cannot be obtained before the final visit, the patient will be considered as “follow-up lost” by the investigator.

In case of early trial discontinuation, the reason(s) for discontinuation must be included in the CRF. If more than one reason, the investigator will inform only about the main reason.

The patient will be asked to attend the programmed visits until the end of the study is announced, even if the study treatment is permanently discontinued, in order to allow the investigator to collect as much data as possible, including safety parameters.

Patients have the right to withdraw from the trial at any time, and any patient can be removed from the trial for any reason beneficial for his/her health.

According to the Good Clinical Practice, all the patients who early discontinued from the study will be offered an alternative treatment. If the withdrawal is due to a significant adverse event, the investigator will monitor the patients until the adequate finalization, that is, until the adverse event disappears or it is determined that the event is permanent.

## CLINICAL TRIAL DISCONTINUATION

The study could be interrupted in case of any Serious Adverse Event related to the treatment or in any of the following situations:

1. Serious toxicity related to the infusion in 1/3 or 2/30 patients.
2. Serious infections in > 2/30 patients.
3. Mortality related to the procedure in ≥ 1 patients.
4. Less than 3 patients included during the first 12 months since the trial approval and distribution.

## EXPECTED NUMBER OF PATIENTS

A total of 30 clinically assessable patients (10 patients in each arm) for evaluation of security and feasibility.

## IDENTIFICATION OF PATIENTS

The patients will be identified by a numerical code of sequential identification, which will be assigned to every patient according to correlative order of incorporation when they give the informed consent. In case a patient is included in the study, meaning singed the informed consent, and once the procedures of the visit of selection are performed and the patient is considered to be not suitable to continue in the study or the patient withdraws his consent, the number that this patient had been assigned will not be able to be re-used for another patient.

The sponsor only will be able to identify the subjects with the code that has been assigned to them, its date of birth and its sex.

The investigator is required to have a record list with the names of the patients and the number of identification assigned.

# ETHICAL ASPECTS

## GENERAL CONSIDERATIONS

This clinical trial will be conducted in compliance with the protocol, according to the Standard Operating Procedure (SOPs) of the sponsor or the designated organization.

The clinical trial will be conducted following the recommendations for clinical trials and assessment of medicinal products for human beings in research phase, as contained in the Declaration of Helsinki, revised at the successive Worldwide General Assemblies (WMA, 2008) (see Annex XII), and the current Spanish legislation on clinical trials (RD 223/2004). The ICG-GCP guidelines (CPMP/ICH/135/95) will be followed.

The investigational product is in line with the definition of “Somatic cell therapy medicinal product” as described at the Order SCO/3461/2003 from November 26^th.^

The trial protocol, including the patient information and informed consent to be used, must be reviewed and approved by the appropriate Ethic Committee. Prior to performing any trial-related procedures describe in the protocol, the informed consent approved by the Ethic Committee shall be signed and dated by the patient.

## INFORMATION TO BE PROVIDED TO THE PATIENTS AND PATIENT INFORMED CONSENT TO BE REQUESTED AT THE TRIAL

The patient informed consent will always be handed in writing. The informed consent form will be signed and dated by the subject before being included in the trial, that is, before performing any trial-related procedures. The investigator is responsible for giving the patient complete information about the nature, purpose, related procedures, planned duration, possible risks and benefits and any discomfort that the trial may involve. A copy of the Patient Information and the Patient Informed Consent Form will be given to the patient by the investigator. Patients taking part in the trial must also be notified that the trial participation is voluntary and that they have the right to withdraw from the trial at any time without giving any explanations and without responsibility of any prejudge, and without affecting any further medical treatment or his/her relation with the medical doctor treating her/him. The subjects must have reasonable time to read and digest the information before signing or withdrawing.

If the final protocol is amended in a way that may affect directly the participation of the subject in the study (for example, a change in any procedure), the Patient Information and the Patient Informed Consent Form must be changed in order to include this modification and the subject must sign the amended form to indicate that he/she still consents to participate in the trial.

The investigator is in charge of handing a copy of the EC approved Patient Information and the Patient Informed Consent Form to the subject or his/her legal representative.

The subject will consent in writing. The subject must fulfil the Informed Consent form by himself/herself. Once the form is signed and dated by the participant subject (or legal representative) and by the investigator, a copy will be given to the patient. The original document will be kept by the investigator, in the Investigator Study File. No patient will be able to participate in the trial without previously signing the Patient Informed Consent.

## CLINICAL TRIAL DATA ACCESS

In order to guarantee clinical trial data confidentiality, the original data will be kept at the hospital and will be made accessible only to the investigator, the collaboration team, the clinical trial monitor and the appropriate Ethics Committee. The investigator will allow the Spanish or European Health Authorities to perform audits and inspections.

## PROTECTION OF STUDY DATA OBTAINED DURING THE STUDY

The content of the Case Report Form (CRF), as well as the data confidentiality of each subject will be protected at all times. Relevant procedures will be followed to ensure the compliance with the Spanish Data Protection Act 15/99 of 13^th^ December.

The documents generated during the study will be protected against not permitted use by individuals outside the investigation and, therefore, it will be considered strictly confidential and it will not be disclosed to third parties, except for what is specified in the previous section.

The investigator will inform patients that the results will be stored and analyse in a computer according to the Spanish regulations on computerized data management.

The investigator accepts the sponsor's right to use the results of the clinical trial including CRF pages or copies. In order to allow the use of the information obtained from the clinical trial, the investigator must provide the sponsor with the complete procedure results and all the information developed during the study.

Participant subject confidentiality will be maintained at any time. The clinical trial results or conclusions will be published primarily in scientific journals before being disclosed to the general public. Procedures with feasibility which had not yet been established will not be disclosed in a premature or sensationalist way.

## CLINICAL TRIAL INSURANCE

Fundación Progreso y Salud, as sponsor of the trial, in accordance with the Spanish legislation has a liability insurance. This policy covers all the potential damages caused by the administration of the study product, in accordance with the current legislation (RD 223/2004, article 8).

## STATISTICAL ANALYSIS

Given that this was an exploratory study and that previous efficacy and safety studies with AdMSCs were lacking, no formal sample size calculations were performed. In order to provide an indication of safety and dose-effect and to support future larger efficacy studies, 30 evaluable patients were considered sufficient.

For the purposes of this study, the safety analysis population was defined as all patients who underwent a lipectomy procedure. The intention-to-treat population comprised all patients who were randomized and received an infusion and the per protocol population comprised patients which accomplished the inclusion criteria with at least 1 post baseline visit.

## DEMOGRAPHIC AND BASAL DATA

A descriptive analysis of all demographic variables collected as well as clinical data prior to initial treatment will be carried out. Qualitative variables will be expressed by absolute frequencies and percentages, while quantitative variables will be presented through the mean, median, standard deviation, maximum, minimum and number of observations.

## FEASIBILITY ANALYSIS

All p-values and confidence intervals will be calculated and evaluated using a bilateral confidence level of 95%. The main analysis will be accomplished by means of:

DEPENDENT VARIABLES: Clinical Variables: neurological outbreak and disfunction: EDSS, MSFC.

INDEPENDENT VARIABLES: General and immunological analytics variables,CSF, evoked potentials (EP), OCD, magnetic resonance, quality of life. Correlation coefficient will be calculated in order to evaluate whether there is correlation between outbreaks and clinical progression of the disease (EDSS and MSFC scales) or not. An exploratory analysis will be carried out to test normality of each variable (EP and clinical disease progression) through a Shapiro - Wilk test. In the case of both variables showing a normal distribution, Pearson correlation coefficient will be used; on the contrary, Spearman correlation coefficient will be used. A 95% confidence level will be used in order to determine if the obtained correlation coefficients are statistically significant. The intraclass correlation coefficient (calculated from the variance analysis results) will be used in order to evaluate concordance between the two quantitative variables corresponding to measurements of 1) functional alterations detected through EP and 2) alterations observed through other possible techniques (general and immunological analysis, CSF, imaging techniques, OCD, neuropsychological tests, quality of life scales...). Concordance strength between the measures of the same attributes will be considered as good if the coefficient value is above 0.71. (Classification used by Fleiss JL. The design and analysis of clinical experiments. New York: Wiley, 1986). A lower value would suggest that the concordance between one measurement and another would be unacceptable. We will also use an alternative graphical procedure proposed by Bland JM and Altman DG (Bland JM, Altman DG (1986) Statistical methods for assessing agreement between two methods of clinical measurement, Lancet i: 307-310), consisting in representing the difference between each pair values ​​versus the mean values from each pair. This method will give us concordance limits from confidence interval calculation for difference of two measurements, which will help us to conclude whether the observed differences are clinically relevant or not. Multivariate analysis techniques will be applied to determinate the association modifying factors. Results comparison between groups will be done between experimental group and control group and between groups according to the dose received. As a result, the treatment effect of the two doses of treatment vs placebo was analyzed by t-test for paired measures, non-parametric tests and ANOVA of repeated measures for the clinical and paraclinical test performed at baseline, 6 and 12 months.

## SAFETY ANALYSIS.

For safety analysis, it will be calculated the number and percentage of patients who give up on the study due to adverse events, patients who have suffered at least one adverse event, most frequent adverse events, and patients who have suffered at least one serious adverse event. In addition, the 95% confidence interval will be calculated.

## STUDY CHALLENGES AND LIMITATIONS.

The most remarkable clinical feature of Multiple Sclerosis is its great variability, since the location of demyelinating lesions that occur throughout the axon determine symptoms and signs. The main limitation is the difficulty to determine the presence/absence of outbreaks and the variability in the clinical outcome measures of disease progression with EDSS and MSFC scales. To minimize this problem, it is established the presence of two neurologists with extensive experience in the diagnosis and follow-up of MS patients.

One of the limitations of this project is its short-term, because of in secondary progressive multiple sclerosis (SPMS) few outbreaks are expected and the disability progression is very slow measured by current clinical scales. For this reason, we will use paraclinical methods for feasibility evaluation (MNR, evoked potential, etc.). Another difficulty is the low number of patients susceptible to be treated due to the slowness to obtain cells in the available laboratory.
