# Supplementary material for: Adipose-derived mesenchymal stem cells (AdMSC) for the treatment of secondary-progressive multiple sclerosis: A triple blinded, placebo controlled, randomized phase I/II safety and feasibility study
Source: PLoS One. 2018 May 16;13(5):e0195891. doi: 10.1371/journal.pone.0195891 (PMC5955528; doi:10.1371/journal.pone.0195891)
Supplement: S5 File — (PDF) [file pone.0195891.s005.pdf]

ClinicalTrials.gov is getting an update. Our new design arrives on June 19th. Learn more.

Preview our new design at ClinicalTrials.gov/beta/

Show less

ClinicalTrials.gov

IMPORTANT: Listing of a study on this site does not reflect endorsement by the National Institutes of Health. Talk with a trusted healthcare professional before volunteering for a study. Read more...

Autologous Mesenchymal Stem Cells From Adipose Tissue in Patients With Secondary Progressive Multiple Sclerosis

This study has been completed.

Sponsor:
Andalusian Initiative for Advanced Therapies - Fundación Pública Andaluza Progreso y Salud

Collaborator:
Carlos III Health Institute

Information provided by (Responsible Party):
Andalusian Initiative for Advanced Therapies - Fundación Pública Andaluza Progreso y Salud

ClinicalTrials.gov Identifier:
NCT01056471

First received: January 25, 2010
Last updated: August 4, 2015
Last verified: February 2015
History of Changes

Purpose

The main purpose of this study is to evaluate the safety and feasibility of regenerative therapy with mesenchymal stem cells from adipose tissue, administered intravenously in patients with secondary progressive multiple sclerosis who do not respond to treatment.

Condition	Intervention	Phase
Autoimmune Diseases<br>Immune System Diseases<br>Demyelinating Diseases<br>Nervous System Diseases<br>Demyelinating Autoimmune Diseases, CNS<br>Autoimmune Diseases of the Nervous System	Other: Autologous mesenchymal stem cells from adipose tissue.	Phase 1<br>Phase 2

Study Type: Interventional
Study Design: Allocation: Randomized
Intervention Model: Parallel Assignment
Masking: Double Blind (Participant, Care Provider, Investigator, Outcomes Assessor)
Primary Purpose: Treatment

Official Title: Multicenter Clinical Trial Phase I / II Randomized, Placebo-controlled Study to Evaluate Safety and Feasibility of Therapy With Two Different Doses of Autologous Mesenchymal Stem Cells in Patients With Secondary Progressive Multiple Sclerosis Who do Not Respond to Treatment

Resource links provided by NLM:

Genetics Home Reference related topics: multiple sclerosis

MedlinePlus related topics: Multiple Sclerosis

U.S. FDA Resources

Further study details as provided by Andalusian Initiative for Advanced Therapies - Fundación Pública Andaluza Progreso y Salud:

Primary Outcome Measures:

- To evaluate safety and tolerability related to the intravenous infusion of autologous mesenchymal stem cells [Time Frame: 12 months.]

Secondary Outcome Measures:

- To evaluate effects on MS disease activity measured by: clinical variables, imaging variables, immunological and neurophysiologic analysis, neuropsychological and quality of life scales. [Time Frame: 12 months]

Enrollment:	30
Study Start Date:	January 2010
Study Completion Date:	June 2015
Primary Completion Date:	June 2012 (Final data collection date for primary outcome measure)

Arms	Assigned Interventions
Experimental: Low dose autologous mesenchymal cells<br>The dose of infused cells is 10e6 cells/Kg	Other: Autologous mesenchymal stem cells from adipose tissue.<br>Intravenous infusion of autologous mesenchymal stem cells. Dose: 10e6 cells/Kg.
Experimental: High dose<br>The dose of infused cells is 4*10e6 cells/Kg	Other: Autologous mesenchymal stem cells from adipose tissue.<br>Intravenous infusion of autologous mesenchymal stem cells.Dose:4*10e6 cells/Kg.
No Intervention: Placebo Control	

Eligibility

Ages Eligible for Study:	18 Years and older (Adult, Senior)
Sexes Eligible for Study:	All
Accepts Healthy Volunteers:	No

Criteria

Inclusion Criteria:

1. Patients diagnosed with Multiple Sclerosis (Poser and McDonald criteria).
2. Secondary progressive MS patients with EDSS ≥ 5.5 and ≤ 9.
3. Patients with treatment failure defined by: no response to immunomodulators / immunosuppressants, and showing activity in the form of 1 relapse in the last year or 0.5 points in EDSS progression.
4. Patients with no MS relapse and no steroid treatment within the month prior to inclusion.
5. Patients who give written consent to participate in the study. -

Exclusion Criteria:

1. History of current pathology or current laboratory results indicative of any severe disease.
2. Pacemaker or metallic implants that prevent MR imaging.
3. Inability to complete questionnaires.
4. Refusal to give informed consent.
5. Predicted impossibility for a biopsy of at least 30 grams of fat tissue.
6. Positive screening test for HIV, Hepatitis B or Hepatitis C.
7. History of malignancy.
8. Having been in treatment with any investigational drug or have undergone any experimental procedure in the 3 months prior to baseline.
9. Body mass index> 40 kg/m2.
10. Patients who have been treated with prohibited concomitant medication during the month prior to inclusion in the study.
11. Pregnancy or lactation

Contacts and Locations

Choosing to participate in a study is an important personal decision. Talk with your doctor and family members or friends about deciding to join a study. To learn more about this study, you or your doctor may contact the study research staff using the Contacts provided below. For general information, see Learn About Clinical Studies.

Please refer to this study by its ClinicalTrials.gov identifier: NCT01056471

Locations

Spain

Hospital Regional Universitario de Málaga
Málaga, Spain, 29010

Hospital Universitario Virgen Macarena
Sevilla, Spain, 41004

Sponsors and Collaborators

Andalusian Initiative for Advanced Therapies - Fundación Pública Andaluza Progreso y Salud

Carlos III Health Institute

Investigators

Study Director:	Oscar Fernandez Fernandez, MD, PhD	Hospital Regional Universitario Carlos Haya, Málaga, Spain.
Principal Investigator:	Guillermo Izquierdo Ayuso, MD, PhD	Hospital Universitario Virgen Macarena, Sevilla, Spain

More Information

Additional Information:

Andalusian Molecular Biology and Regenerative Medicine Centre

Responsible Party:	Andalusian Initiative for Advanced Therapies - Fundación Pública Andaluza Progreso y Salud
ClinicalTrials.gov Identifier:	NCT01056471 History of Changes
Other Study ID Numbers:	CMM/EM/2008
Study First Received:	January 25, 2010
Last Updated:	August 4, 2015

Keywords provided by Andalusian Initiative for Advanced Therapies - Fundación Pública Andaluza Progreso y Salud:
Multiple Sclerosis
Mesenchymal Stem Cells
Autologous

Additional relevant MeSH terms:
Sclerosis
Multiple Sclerosis
Nervous System Diseases
Autoimmune Diseases
Immune System Diseases
Multiple Sclerosis, Chronic Progressive
Demyelinating Autoimmune Diseases, CNS
Autoimmune Diseases of the Nervous System
Demyelinating Diseases
Pathologic Processes
Leukoencephalopathies
Brain Diseases
Central Nervous System Diseases

ClinicalTrials.gov processed this record on June 02, 2017
