# Supplementary material for: Adipose-derived mesenchymal stem cells (AdMSC) for the treatment of secondary-progressive multiple sclerosis: A triple blinded, placebo controlled, randomized phase I/II safety and feasibility study
Source: PLoS One. 2018 May 16;13(5):e0195891. doi: 10.1371/journal.pone.0195891 (PMC5955528; doi:10.1371/journal.pone.0195891)
Supplement: S6 File — (PDF) [file pone.0195891.s006.pdf]

ClinicalTrials.gov is getting an update. Our new design arrives on June 19th. Learn more.

Preview our new design at ClinicalTrials.gov/beta/

Show less

ClinicalTrials.gov

IMPORTANT: Listing of a study on this site does not reflect endorsement by the National Institutes of Health. Talk with a trusted healthcare professional before volunteering for a study. Read more...

Autologous Mesenchymal Stem Cells From Adipose Tissue in Patients With Secondary Progressive Multiple Sclerosis

This study has been completed.

Sponsor:
Andalusian Initiative for Advanced Therapies - Fundación Pública Andaluza Progreso y Salud

Collaborator:
Carlos III Health Institute

Information provided by (Responsible Party):
Andalusian Initiative for Advanced Therapies - Fundación Pública Andaluza Progreso y Salud

ClinicalTrials.gov Identifier:
NCT01056471

First received: January 25, 2010
Last updated: August 4, 2015
Last verified: February 2015
History of Changes

Tracking Information

**First Received Date** ICMJE	January 25, 2010
**Last Updated Date**	August 4, 2015
**Start Date** ICMJE	January 2010
**Primary Completion Date**	June 2012 (Final data collection date for primary outcome measure)
**Current Primary Outcome Measures** ICMJE **(submitted: April 23, 2010)**	To evaluate safety and tolerability related to the intravenous infusion of autologous mesenchymal stem cells [Time Frame: 12 months.]
**Original Primary Outcome Measures** ICMJE **(submitted: January 25, 2010)**	To evaluate safety and tolerability related to the intravenous infusion of autologous mesenchymal stem cells [Time Frame: 24 hr, 8 days, 1, 2, 3, 6, 9 and 12 months.]
**Change History**	Complete list of historical versions of study NCT01056471 on ClinicalTrials.gov Archive Site
**Current Secondary Outcome Measures** ICMJE **(submitted: April 23, 2010)**	To evaluate effects on MS disease activity measured by: clinical variables, imaging variables, immunological and neurophysiologic analysis, neuropsychological and quality of life scales. [Time Frame: 12 months]
**Original Secondary Outcome Measures** ICMJE **(submitted: January 25, 2010)**	To evaluate effects on MS disease activity measured by: clinical variables, imaging variables, immunological and neurophysiologic analysis, neuropsychological and quality of life scales. [Time Frame: 24 hr, 8 days, 1, 2, 3, 6, 9 and 12 months]
**Current Other Outcome Measures** ICMJE	*Not Provided*
**Original Other Outcome Measures** ICMJE	*Not Provided*

Descriptive Information

**Brief Title** ICMJE	Autologous Mesenchymal Stem Cells From Adipose Tissue in Patients With Secondary Progressive Multiple Sclerosis
**Official Title** ICMJE	Multicenter Clinical Trial Phase I / II Randomized, Placebo-controlled Study to Evaluate Safety and Feasibility of Therapy With Two Different Doses of Autologous Mesenchymal Stem Cells in Patients With Secondary Progressive Multiple Sclerosis Who do Not Respond to Treatment
**Brief Summary**	The main purpose of this study is to evaluate the safety and feasibility of regenerative therapy with mesenchymal stem cells from adipose tissue, administered intravenously in patients with secondary progressive multiple sclerosis who do not respond to treatment.
**Detailed Description**	*Not Provided*
**Study Type** ICMJE	Interventional
**Study Phase**	Phase 1<br>Phase 2
**Study Design** ICMJE	Allocation: Randomized<br>Intervention Model: Parallel Assignment<br>Masking: Double Blind (Participant, Care Provider, Investigator, Outcomes Assessor)<br>Primary Purpose: Treatment
**Condition** ICMJE	• Autoimmune Diseases<br>• Immune System Diseases<br>• Demyelinating Diseases<br>• Nervous System Diseases<br>• Demyelinating Autoimmune Diseases, CNS<br>• Autoimmune Diseases of the Nervous System
**Intervention** ICMJE	• Other: Autologous mesenchymal stem cells from adipose tissue.<br>Intravenous infusion of autologous mesenchymal stem cells.Dose:4*10e6 cells/Kg.<br>• Other: Autologous mesenchymal stem cells from adipose tissue.<br>Intravenous infusion of autologous mesenchymal stem cells. Dose: 10e6 cells/Kg.
**Study Arms**	• Experimental: Low dose autologous mesenchymal cells<br>The dose of infused cells is 10e6 cells/Kg<br>Intervention: Other: Autologous mesenchymal stem cells from adipose tissue.<br>• Experimental: High dose<br>The dose of infused cells is 4*10e6 cells/Kg<br>Intervention: Other: Autologous mesenchymal stem cells from adipose tissue.<br>• No Intervention: Placebo Control
**Publications** *	*Not Provided*

* **Includes publications given by the data provider as well as publications identified by ClinicalTrials.gov Identifier (NCT Number) in Medline.**

Recruitment Information

**Recruitment Status** ICMJE	Completed
**Enrollment** ICMJE	30
**Completion Date**	June 2015
**Primary Completion Date**	June 2012 (Final data collection date for primary outcome measure)
**Eligibility Criteria** ICMJE	Inclusion Criteria:<br>1. Patients diagnosed with Multiple Sclerosis (Poser and McDonald criteria).<br>2. Secondary progressive MS patients with EDSS ≥ 5.5 and ≤ 9.

	3. Patients with treatment failure defined by: no response to immunomodulators / immunosuppressants, and showing activity in the form of 1 relapse in the last year or 0.5 points in EDSS progression.<br>4. Patients with no MS relapse and no steroid treatment within the month prior to inclusion.<br>5. Patients who give written consent to participate in the study. -<br><br>Exclusion Criteria:<br><br>1. History of current pathology or current laboratory results indicative of any severe disease.<br>2. Pacemaker or metallic implants that prevent MR imaging.<br>3. Inability to complete questionnaires.<br>4. Refusal to give informed consent.<br>5. Predicted impossibility for a biopsy of at least 30 grams of fat tissue.<br>6. Positive screening test for HIV, Hepatitis B or Hepatitis C.<br>7. History of malignancy.<br>8. Having been in treatment with any investigational drug or have undergone any experimental procedure in the 3 months prior to baseline.<br>9. Body mass index> 40 kg/m2.<br>10. Patients who have been treated with prohibited concomitant medication during the month prior to inclusion in the study.<br>11. Pregnancy or lactation
**Sex/Gender**	Sexes Eligible for Study: All
**Ages**	18 Years and older (Adult, Senior)
**Accepts Healthy Volunteers**	No
**Contacts** ICMJE	*Contact information is only displayed when the study is recruiting subjects*
**Listed Location Countries** ICMJE	Spain
**Removed Location Countries**	

Administrative Information

**NCT Number** ICMJE	NCT01056471
**Other Study ID Numbers** ICMJE	CMM/EM/2008
**Has Data Monitoring Committee**	No
**U.S. FDA-regulated Product**	*Not Provided*
**Plan to Share Data**	*Not Provided*
**IPD Description**	*Not Provided*
**Responsible Party**	Andalusian Initiative for Advanced Therapies - Fundación Pública Andaluza Progreso y Salud
**Study Sponsor** ICMJE	Andalusian Initiative for Advanced Therapies - Fundación Pública Andaluza Progreso y Salud
**Collaborators** ICMJE	Carlos III Health Institute
**Investigators** ICMJE	Study Director: Oscar Fernandez Fernandez, MD, PhD — Hospital Regional Universitario Carlos Haya, Málaga, Spain.<br>Principal Investigator: Guillermo Izquierdo Ayuso, MD, PhD — Hospital Universitario Virgen Macarena, Sevilla, Spain
**Information Provided By**	Andalusian Initiative for Advanced Therapies - Fundación Pública Andaluza Progreso y Salud
**Verification Date**	February 2015

ICMJE **Data element required by the International Committee of Medical Journal Editors and the World Health Organization ICTRP**
