# Supplementary material for: Adipose-derived mesenchymal stem cells (AdMSC) for the treatment of secondary-progressive multiple sclerosis: A triple blinded, placebo controlled, randomized phase I/II safety and feasibility study
Source: PLoS One. 2018 May 16;13(5):e0195891. doi: 10.1371/journal.pone.0195891 (PMC5955528; doi:10.1371/journal.pone.0195891)
Supplement: S7 File — (DOCX) [file pone.0195891.s007.docx]

Summary

EudraCT Number: 2008-004015-35

Sponsor's Protocol Code Number: CMM/EM/2008

National Competent Authority: Spain - AEMPS

Clinical Trial Type: EEA CTA

Trial Status: Ongoing

Date on which this record was first entered in the EudraCT database: 2008-07-30

Link: https://www.clinicaltrialsregister.eu/ctr-search/trial/2008-004015-35/ES/

A. Protocol Information

A.1 Member State Concerned: Spain - AEMPS

A.2 EudraCT number: 2008-004015-35

A.3 Full title of the trial: ENSAYO CLÍNICO MULTICÉNTRICO FASE I/II ALEATORIZADO Y CONTROLADO CON PLACEBO, PARA EVALUACIÓN DE SEGURIDAD Y FACTIBILIDAD DE LA TERAPIA CON DOS DOSIS DISTINTAS DE CÉLULAS TRONCALES MESENQUIMALES AUTÓLOGAS DE TEJIDO ADIPOSO (CETMAD) EN PACIENTES CON ESCLEROSIS MÚLTIPLE SECUNDARIAMENTE PROGRESIVA, QUE NO RESPONDEN ADECUADAMENTE A LOS TRATAMIENTOS REGISTRADOS.

A.4.1 Sponsor's protocol code number: CMM/EM/2008

A.7 Trial is part of a Paediatric Investigation Plan: Information not present in EudraCT

A.8 EMA Decision number of Paediatric Investigation Plan:

B. Sponsor Information

Sponsor 1

B.1.1 Name of Sponsor: Fundación Progreso y Salud

B.1.3.4 Country: Spain

B.3.1 and B.3.2 Status of the sponsor: Non-Commercial

B.4 Source(s) of Monetary or Material Support for the clinical trial:

B.4.1 Name of organisation providing support:

B.4.2 Country:

B.5 Contact point designated by the sponsor for further information on the trial

B.5.1 Name of organisation:

B.5.2 Functional name of contact point:

D. IMP Identification

D.IMP: 1

D.1.2 and D.1.3 IMP Role: Test

D.2 Status of the IMP to be used in the clinical trial

D.2.1 IMP to be used in the trial has a marketing authorisation: No

D.2.5 The IMP has been designated in this indication as an orphan drug in the Community: No

D.2.5.1 Orphan drug designation number:

D.3 Description of the IMP

D.3.1 Product name: Células troncales mesenquimales autólogas de tejido adiposo

D.3.2 Product code: CETMAD

D.3.4 Pharmaceutical form: Suspension for injection

D.3.4.1 Specific paediatric formulation: Information not present in EudraCT

D.3.7 Routes of administration for this IMP:

Intravenous use

D.3.8 to D.3.10 IMP Identification Details (Active Substances)

D.3.8 INN - Proposed INN: N/A

D.3.9.1 CAS number: N/A

D.3.9.2 Current sponsor code: Placebo de CETMAD

D.3.9.3 Other descriptive name: Células troncales mesenquimales autólogas de tejido adiposo

D.3.10 Strength

D.3.10.1 Concentration unit: IU/kg international unit(s)/kilogram

D.3.10.2 Concentration type: range

D.3.10.3 Concentration number: 1000000 CeTMAd/kg to 4000000 CeTMAd/kg

D.3.11 The IMP contains an

D.3.11.1 Active substance of chemical origin: No

D.3.11.2 Active substance of biological/ biotechnological origin (other than Advanced Therapy IMP (ATIMP): Yes

D.3.11.3 Advanced Therapy IMP (ATIMP): Information not present in EudraCT

D.3.11.3.1 Somatic cell therapy medicinal product: Yes

D.3.11.3.2 Gene therapy medical product: No

D.3.11.3.3 Tissue Engineered Product: Information not present in EudraCT

D.3.11.3.4 Combination ATIMP (i.e. one involving a medical device): Information not present in EudraCT

D.3.11.3.5 Committee on Advanced therapies (CAT) has issued a classification for this product: Information not present in EudraCT

D.3.11.4 Combination product that includes a device, but does not involve an Advanced Therapy: Information not present in EudraCT

D.3.11.5 Radiopharmaceutical medicinal product: No

D.3.11.6 Immunological medicinal product (such as vaccine, allergen, immune serum): No

D.3.11.7 Plasma derived medicinal product: No

D.3.11.8 Extractive medicinal product: No

D.3.11.9 Recombinant medicinal product: Information not present in EudraCT

D.3.11.10 Medicinal product containing genetically modified organisms: No

D.3.11.11 Herbal medicinal product: No

D.3.11.12 Homeopathic medicinal product: No

D.3.11.13 Another type of medicinal product: No

D.8 Information on Placebo

D.8 Placebo: 1

D.8.1 Is a Placebo used in this Trial? Yes

D.8.3 Pharmaceutical form of the placebo: Suspension for injection

D.8.4 Route of administration of the placebo: Intravenous use

E. General Information on the Trial

E.1 Medical condition or disease under investigation

E.1.1 Medical condition(s) being investigated: Pacientes con esclerosis múltiple secundariamente progresiva, que no responden adecuadamente a los tratamientos registrados.

MedDRA Classification

E.1.3 Condition being studied is a rare disease: No

E.2 Objective of the trial

E.2.1 Main objective of the trial: Evaluar la seguridad y factibilidad de dos dosis de células troncales mesenquimales autólogas administradas por vía intravenosa en pacientes con EM de curso clínico secundariamente progresivo. Se analizarán las complicaciones derivadas de la terapia regenerativa y/o procedimientos del estudio.

E.2.2 Secondary objectives of the trial: Evaluar la seguridad medida por escala de efectos adversos y análisis hematológicos y bioquímicos generales.

Evaluar la factibilidad medida por variables clínicas y paraclínicas.

Evaluar el efecto inmunomodulador.

Evaluar el perfil de expresión génica en células PBMCs y células de LCR y la evaluación de perfiles metabólicos con el objetivo de identificar nuevos biomarcadores con interés diagnóstico, pronóstico o de seguimiento.

E.2.3 Trial contains a sub-study: No

E.3 Principal inclusion criteria: 1. Pacientes de ambos sexos de edad ≥ 18 años.

2. Pacientes diagnosticados de Esclerosis Múltiple (EM) clínicamente definida (criterios de Poser y McDonald)

3. Pacientes con EM secundariamente progresiva, con EDSS ≥ 5,5 y ≤ 9

4. Pacientes con fracaso terapéutico definido por:

4.1 No respondedor a las terapias empleadas (inmunomoduladores-inmunosupresores) y

4.2. que presenten actividad en forma de 1 brote en el último año o progresión de 0,5 puntos en la escala EDSS

5. Pacientes que no hayan presentado brotes en el mes previo a la inclusión, ni recibido tratamiento esteroideo en el mes previo a la inclusión.

6. Pacientes que otorguen su consentimiento por escrito para la participación en el estudio.

E.4 Principal exclusion criteria: 1. Existencia de procesos intercurrentes que puedan previsiblemente alterar las medidas de las variables previstas: Patología hepática, renal, cardíaca, etc y patología psiquiátrica u ocular (glaucoma-se realizará medición de presión intraocular previa a la realización de la TCO, cataratas).

2. Portador de marcapasos o implantes metálicos (p.ej. prótesis) que impida realización de RM.

3. Negativa a firmar el consentimiento informado por escrito.

4. Imposibilidad prevista para obtener una biopsia de al menos 30 g de tejido adiposo limpio.

5. Infección por VIH, Hepatitis B o Hepatitis C.

6. Haber padecido o sufrir actualmente una neoplasia maligna.

7. Haber estado en tratamiento con cualquier fármaco en investigación o haberse sometido a cualquier procedimiento experimental en los 3 meses previos al inicio del estudio.

8. Índice de masa corporal > 40 Kg/m2.

9. Paciente que hayan sido tratados con medicación concomitante prohibida durante el mes anterior a la inclusión en el estudio.

10. Embarazo o lactancia

11. Paciente que haya participado en los últimos 3 meses en otro ensayo clínico.

E.5 End points

E.5.1 Primary end point(s): Se evaluará la seguridad por escala de efectos adversos y análisis hematológicos y bioquímicos generales en las primeras 24h de la administración de las CeTMAd, 1 semana, 1 mes, 2 meses, 3 meses, 6 meses, 9 meses y 12 meses.

Se evaluará la factibilidad por variables clínicas (brotes, progresión medida con las escalas EDSS y MSFC) y paraclínicas (resonancia magnética, potenciales evocados, tomografía óptica de coherencia, pruebas neuropsicológicas y escalas de calidad de

vida)

Se evaluará el efecto inmunomodulador. Para ello se procede a realizar mediciones de subpoblaciones celulares y citocinas de perfil pro y antiinflamatorio en suero y líquido cefalorraquídeo. Asimismo, se procede al análisis funcional de la respuesta inmune en PBMCs, células B, células dendríticas y la actividad funcional de células CD4+CD25+CD62Lhigh y CD4+CD25-CD62Llow.

Se evaluará el perfil de expresión génica en células PBMCs y células de LCR mediante “arrays”, así como el estudio en paneles de “antigen arrays” y la evaluación de perfiles metabolómicos mediante NMR en suero y LCR con el objetivo de identificar nuevos biomarcadores con interés diagnóstico, pronóstico o de seguimiento.

Se estudiará la factibilidad en pacientes de EM que no hayan respondido a otros tratamientos. Estará controlado con placebo.

Aunque el período de seguimiento para la obtención de resultados de seguridad y factibilidad es de 12 meses, dado que estos pacientes se ven de forma rutinaria en consulta de los centros participantes, en el estudio se recopilarán datos a los 24 meses para obtener información adicional de seguridad y factibilidad a medio plazo con un protocolo determinado y previa autorización de las autoridades sanitarias pertinentes.

E.6 and E.7 Scope of the trial

E.6 Scope of the Trial

E.6.1 Diagnosis: No

E.6.2 Prophylaxis: No

E.6.3 Therapy: Yes

E.6.4 Safety: Yes

E.6.5 Efficacy: Yes

E.6.6 Pharmacokinetic: No

E.6.7 Pharmacodynamic: No

E.6.8 Bioequivalence: No

E.6.9 Dose response: Yes

E.6.10 Pharmacogenetic: No

E.6.11 Pharmacogenomic: No

E.6.12 Pharmacoeconomic: No

E.6.13 Others: Yes

E.6.13.1 Other scope of the trial description: Factibilidad

E.7 Trial type and phase

E.7.1 Human pharmacology (Phase I): Yes

E.7.1.1 First administration to humans: No

E.7.1.2 Bioequivalence study: No

E.7.1.3 Other: Yes

E.7.1.3.1 Other trial type description: Aplicación de CETMAD en paciente con EMSP

E.7.2 Therapeutic exploratory (Phase II): Yes

E.7.3 Therapeutic confirmatory (Phase III): Information not present in EudraCT

E.7.4 Therapeutic use (Phase IV): Information not present in EudraCT

E.8 Design of the trial

E.8.1 Controlled: Yes

E.8.1.1 Randomised: Yes

E.8.1.2 Open: No

E.8.1.3 Single blind: No

E.8.1.4 Double blind: No

E.8.1.5 Parallel group: Yes

E.8.1.6 Cross over: No

E.8.1.7 Other: Yes

E.8.1.7.1 Other trial design description: Triple ciego

E.8.2 Comparator of controlled trial

E.8.2.1 Other medicinal product(s): No

E.8.2.2 Placebo: Yes

E.8.2.3 Other: No

E.8.3 The trial involves single site in the Member State concerned: No

E.8.4 The trial involves multiple sites in the Member State concerned: Yes

E.8.4.1 Number of sites anticipated in Member State concerned: 2

E.8.5 The trial involves multiple Member States: No

E.8.6 Trial involving sites outside the EEA

E.8.6.1 Trial being conducted both within and outside the EEA: No

E.8.6.2 Trial being conducted completely outside of the EEA: Information not present in EudraCT

E.8.7 Trial has a data monitoring committee: No

E.8.8 Definition of the end of the trial and justification where it is not the last visit of the last subject undergoing the trial: Desde la infusión de las CETMAD al paciente hasta el fin del seguimiento serán 12 meses.

E.8.9 Initial estimate of the duration of the trial

E.8.9.1 In the Member State concerned years:

E.8.9.1 In the Member State concerned months: 48

E.8.9.1 In the Member State concerned days:

F. Population of Trial Subjects

F.1 Age Range

F.1.1 Trial has subjects under 18: No

F.1.1.1 In Utero: No

F.1.1.2 Preterm newborn infants (up to gestational age < 37 weeks): No

F.1.1.3 Newborns (0-27 days): No

F.1.1.4 Infants and toddlers (28 days-23 months): No

F.1.1.5 Children (2-11years): No

F.1.1.6 Adolescents (12-17 years): No

F.1.2 Adults (18-64 years): Yes

F.1.3 Elderly (>=65 years): Yes

F.2 Gender

F.2.1 Female: Yes

F.2.2 Male: Yes

F.3 Group of trial subjects

F.3.1 Healthy volunteers: No

F.3.2 Patients: Yes

F.3.3 Specific vulnerable populations: Yes

F.3.3.1 Women of childbearing potential not using contraception : No

F.3.3.2 Women of child-bearing potential using contraception: Yes

F.3.3.3 Pregnant women: No

F.3.3.4 Nursing women: No

F.3.3.5 Emergency situation: No

F.3.3.6 Subjects incapable of giving consent personally: No

F.3.3.7 Others: No

F.4 Planned number of subjects to be included

F.4.1 In the member state: 30

F.4.2 For a multinational trial

G. Investigator Networks to be involved in the Trial

N. Review by the Competent Authority or Ethics Committee in the country concerned

N. Competent Authority Decision: Authorised

N. Date of Competent Authority Decision: 2009-08-25

N. Ethics Committee Opinion of the trial application: Favourable

N. Ethics Committee Opinion: Reason(s) for unfavourable opinion:

N. Date of Ethics Committee Opinion: 2008-07-29

P. End of Trial

P. End of Trial Status: Ongoing
