# Supplementary material for: Adipose-derived mesenchymal stem cells (AdMSC) for the treatment of secondary-progressive multiple sclerosis: A triple blinded, placebo controlled, randomized phase I/II safety and feasibility study
Source: PLoS One. 2018 May 16;13(5):e0195891. doi: 10.1371/journal.pone.0195891 (PMC5955528; doi:10.1371/journal.pone.0195891)
Supplement: S9 File — (PDF) [file pone.0195891.s009.pdf]

		Placebo				Low dose				High dose					
		Baseline	Final	Outcome (12 months)	p.within	Baseline	Final	Outcome (12 months)	p.within	Baseline	Final	Outcome (12 months)	p.within	p.F	p.kw
EDSS	EDSS	7.64±0.31	7.55±0.35	-0.09±0.17	0.606	7.50±0.24	7.75±0.24	0.25±0.09	0.019*	7.78±0.16	8.06±0.41	0.28±0.52	0.610	0.577	0.209
MRI	MRI T1 number of lesions	62.18±7.18	63.78±8.44	1.22±0.68	0.110	46.40±9.45	46.60±9.46	0.20±0.81	0.811	40.56±6.70	39.89±6.74	-0.67±0.71	0.373	0.195	0.104
	MRI T1 area (cm^2)	197.17±71.85	97.61±18.16	0.34±1.08	0.763	111.51±35.26	119.25±34.89	7.74±5.17	0.169	94.78±34.99	93.85±33.76	-0.93±1.43	0.535	0.144	0.333
	MRI T1GAD number of lesions	0.82±0.37	1.40±0.63	0.50±0.63	0.450	0.70±0.52	0.60±0.42	-0.10±0.25	0.694	2.00±1.09	1.25±0.87	-0.75±0.72	0.333	0.269	0.623
	MRI T1GAD area (cm^2)	0.01±0.00	0.01±0.01	0.00±0.01	0.488	0.01±0.01	0.02±0.01	0.01±0.02	0.551	0.02±0.01	0.01±0.01	-0.01±0.01	0.299	0.479	0.918
	MRI T2 number of lesions	159.00±24.79	166.90±26.40	-1.30±2.79	0.653	152.40±16.62	154.90±15.37	2.50±2.67	0.373	140.75±13.34	143.25±13.27	2.50±1.82	0.212	0.441	0.635
	MRI T2 area (cm^2)	440.71±130.06	387.90±129.33	16.42±9.72	0.125	298.95±67.02	307.61±69.24	8.66±5.16	0.128	296.70±57.61	320.48±61.56	23.78±19.49	0.257	0.665	0.784
	MRI normalized cerebral volume (cm^3)	1420.59±24.17	1439.96±17.90	-5.10±7.09	0.492	1483.74±26.73	1460.21±34.06	-23.53±11.79	0.077.	1449.17±32.65	1437.78±30.59	-11.40±19.77	0.582	0.558	0.235
	MRI non normalized cerebral volume (cm^3)	1062.93±37.94	1090.30±35.63	-11.84±7.28	0.142	1112.66±28.93	1082.87±34.89	-29.79±11.92	0.034*	1036.82±58.53	1042.54±55.95	5.73±24.10	0.819	0.231	0.276
	MTR	18.10±0.51	16.47±0.51	-1.21±0.62	0.082.	18.30±0.49	16.29±0.41	-2.00±0.62	0.010*	19.06±0.78	17.04±0.34	-2.02±0.86	0.050.	0.599	0.504
Evoked potentials	VEP p100 Latency (ms)	134.45±11.91	143.72±6.70	18.00±15.21	0.271	164.82±12.11	164.23±9.64	-0.58±13.63	0.967	156.49±9.63	136.51±13.98	-13.48±14.77	0.397	0.310	0.163
	VEP amplitude (μV)	4.02±0.88	4.04±0.74	-0.71±0.71	0.350	3.26±0.78	2.82±1.02	-0.44±0.74	0.567	4.31±1.30	2.91±0.87	-2.41±0.78	0.021*	0.147	0.211
	BAEP I-V Interval (ms)	3.86±0.12	3.97±0.17	0.03±0.17	0.849	4.46±0.19	4.22±0.16	-0.24±0.25	0.363	4.26±0.15	4.16±0.11	0.04±0.15	0.785	0.505	0.871
	BAEP V/I amplitude	37.62±14.54	27.58±16.19	3.26±11.26	0.779	38.36±16.82	32.67±15.38	-5.69±17.83	0.757	25.50±12.49	10.51±10.63	-16.06±15.70	0.353	0.694	0.664
	Median nerve SEP (N13-N20) (ms)	12.82±1.14	12.42±0.97	-0.76±1.21	0.544	24.71±10.50	14.23±0.68	-10.49±10.64	0.350	17.57±5.85	11.35±1.65	-6.76±6.88	0.363	0.599	0.897
	Tibial nerve SEP (N22-P39) (ms)	43.62±2.64	34.84±1.57	-8.42±2.56	0.009*	44.38±1.61	35.61±2.09	-8.76±2.88	0.014*	45.74±1.60	34.06±1.95	-11.24±2.41	0.003*	0.732	0.602
	MEP superior CCT (ms)	48.25±11.26	26.19±5.35	-20.11±10.02	0.076.	44.48±12.70	28.33±6.08	-16.15±10.55	0.160	51.76±15.61	22.32±4.17	-30.90±16.72	0.114	0.670	0.736
	MEP inferior CCT (ms)	72.70±5.73	43.11±2.28	-28.68±6.96	0.003*	75.92±6.30	40.74±3.58	-35.18±4.82	<0.001*	75.07±7.25	45.48±1.21	-27.64±9.76	0.030*	0.675	0.901
	P300 Latency (ms)	380.14±32.04	404.40±30.35	42.24±46.39	0.404	332.20±21.48	369.18±27.13	22.13±11.44	0.125	373.92±21.61	374.65±23.10	-4.22±12.93	0.765	0.595	0.256
	EPAS	24.27±1.36	24.30±1.10	-0.10±1.57	0.951	23.70±1.99	25.60±1.37	1.90±1.07	0.110	22.78±1.32	23.43±1.22	1.43±1.00	0.201	0.467	0.457
OCT	Optic coherence tomography RNFL (μm)	70.95±6.11	74.50±3.40	-1.06±2.97	0.731	72.00±5.23	70.19±5.27	-1.94±3.56	0.603	67.56±5.27	70.64±3.05	-1.79±2.19	0.447	0.972	0.739
Cognition and QoL	PASAT	36.50±8.09	40.67±6.57	4.17±1.86	0.076.	30.17±4.37	29.20±4.22	-2.60±2.41	0.342	31.00±7.10	32.75±11.86	0.25±3.24	0.943	0.102	0.110
	QoL (EQ5D)	45.91±8.27	47.22±6.44	-4.44±7.78	0.583	47.03±9.25	44.50±6.96	-2.53±6.95	0.724	50.00±7.76	45.62±6.96	1.88±6.62	0.785	0.810	0.737
